# Silver(I)-NHC Complexes as Dual-Action Agents Against Pathogenic *Acanthamoeba* Trophozoites: Anti-Amoebic and Anti-Adhesion Activities

**DOI:** 10.3390/ijms26199393

**Published:** 2025-09-25

**Authors:** Shaima Hkiri, Neslihan Şahin, Zübeyda Akın-Polat, Elvan Üstün, Bui Minh Thu Ly, İsmail Özdemir, David Sémeril

**Affiliations:** 1 Synthèse Organométallique et Catalyse, UMR-CNRS 7177, Strasbourg University, 67008 Strasbourg, France; hkiri@unistra.fr (S.H.); bui-minh-thu.ly@etu.unistra.fr (B.M.T.L.); 2 Department of Science Education, Faculty of Education, Cumhuriyet University, Sivas 58040, Türkiye; 3 Department of Engineering Basic Sciences, Faculty of Engineering and Natural Sciences, Malatya Turgut Özal University, Malatya 44900, Türkiye; 4 Departments of Medical Parasitology, Cumhuriyet University School of Medicine, Sivas 58140, Türkiye; zakinpolat@cumhuriyet.edu.tr; 5 Department of Chemistry, Faculty of Art and Science, Ordu University, Ordu 52200, Türkiye; elvanustun77@gmail.com; 6 Drug Application and Research Center, İnönü University, Malatya 44280, Türkiye; iozdemir44@gmail.com

**Keywords:** silver complex, *N*-heterocyclic carbene, *Acanthamoeba*, in vitro evaluation, anti-amoebic activity, anti-adhesion activity, molecular docking

## Abstract

A series of six silver(I) complexes, namely bromo(1-benzyl-3-cinnamyl-benzimidazol-2-ylidene)silver (I) (**1a**), bromo[1-(4-methylbenzyl)-3-cinnamyl-benzimidazol-2-yliden]silver(I) (**1b**), bromo[1-(3-methoxylbenzyl)-3-cinnamyl-benzimidazol-2-yliden]silver(I) (**1c**), bromo[1-(3,5-dimethoxy-benzyl)-3-cinnamyl-benzimidazol-2-ylidene]silver(I) (**1d**), bromo[1-(naphthalen-1-ylmethyl)-3-cinnamyl-benzimidazol-2-ylidene]silver(I) (**1e**) and bromo[1-(pyren-1-ylmethyl)-3-cinnamyl-benzimidazol-2-yliden]silver(I) (**1f**), were synthetized and characterized by microanalyses and mass spectrometry and characterized by FT-IR and NMR spectroscopic techniques. The in vitro effects of silver(I) complexes on trophozoites of two *Acanthamoeba* isolates obtained from patients with keratitis were investigated. The parasites were exposed to concentrations of 10, 100 and 1000 µM for 24, 48 and 72 h. The complexes exhibited potent, dose- and time-dependent activity. Complete inhibition was observed within 24 h at a concentration of 1000 µM. At a concentration of 100 µM, complexes **1c**–**e** exhibited reduced viability to less than 10% within 48 to 72 h. At a concentration of 10 µM, partial inhibition was observed. Preliminary morphological changes included the loss of acanthopodia, rounding, and detachment. These effects were not observed in the presence of the pre-ligands or commercially available silver compounds. Furthermore, molecular docking was utilized to analyze the molecules against *Acanthamoeba castellanii* CYP51, *A. castellanii* profilin IA, IB, and II. The highest recorded interactions were identified as −9.85 and −11.26 kcal/mol for **1e** and **1f**, respectively, when evaluated against the *A. castellanii* CYP51 structure.

## 1. Introduction

*Acanthamoeba* is a genus of ubiquitous, single-celled, free-living amoebae. These organisms are commonly found in a variety of natural and man-made environments, including soil, freshwater, marine habitats, dust and air [1,2,3]. These amoebae exhibit a biphasic life cycle, alternating between an active trophozoite stage and a highly resilient cyst stage. The cyst form, characterized by a double-walled cellulose structure, enables *Acanthamoeba* to survive extreme environmental conditions, including desiccation, temperature fluctuations, pH extremes, and exposure to disinfectants and anti-amoebic agents [4,5]. This resilience poses significant challenges in both environmental management and clinical treatment.

While the majority of *Acanthamoeba* species fulfill beneficial ecological functions, several have been identified as opportunistic pathogens in humans and animals [2,6,7]. Clinically, *Acanthamoeba* is most notably associated with *Acanthamoeba keratitis* (AK), a painful and potentially blinding infection of the cornea, and granulomatous amoebic encephalitis, a rare but often fatal central nervous system infection [8,9,10,11]. The global increase in contact lens use over the past three decades has led to a corresponding rise in the incidence of AK. Presently, AK is regarded as a primary contributing factor to microbial keratitis in developed nations. Nonetheless, the initial clinical indications, including ocular discomfort, erythema, photophobia, and visual impairment, are generally non-specific and frequently result in delayed diagnoses, which negatively impact prognoses. [3,12,13,14]. In immunocompromised individuals, *Acanthamoeba* can also cause cutaneous and pulmonary infections, further highlighting its pathogenic versatility [15].

The treatment of AK is complex and prolonged, requiring agents that are effective against both trophozoites and cysts. Trophozoites exhibit a heightened sensitivity to antibiotics, antifungals, antiprotozoals, and antiseptics, while cysts demonstrate a marked resilience, necessitating the application of compounds such as polyhexamethylene biguanide, chlorhexidine, and diamidines, including propamidine and hexamidine [3,16,17]. The efficacy of therapeutic interventions is often contingent on the prompt initiation of treatment; however, treatment regimens may span weeks to months, and incomplete eradication can lead to recurrence or vision loss [10,12,18]. In cases of refractory disease, corneal transplantation may be necessary [1,6,19,20].

*N*-Heterocyclic carbenes (NHCs) have undergone a transition from the status of mere chemical curiosities to that of essential ligands in both organometallic and bioorganometallic chemistry. This transition is attributable to their exceptional σ-donating and π-accepting characteristics [21]. Their initial development by Öfele and Wanzlick, followed by Arduengo’s seminal isolation of a stable free carbene, marked a pivotal moment in carbene chemistry and opened new avenues for the synthesis of robust metal complexes [22,23,24].

A notable advantage of NHCs is their structural adaptability, which can be achieved through modifications, particularly at the nitrogen atoms, through substitution. This structural flexibility enables precise control over key properties such as steric hindrance, stability, and lipophilicity [25,26]. Complexes of NHCs with late transition metals, such as palladium, silver, copper, ruthenium, and gold, have demonstrated notable biological activities, positioning them as promising candidates for further therapeutic development [27,28,29]. Collectively, these advancements underscore the mounting importance of NHC-metal complexes in the domain of medicinal chemistry and their prospective role as multifunctional agents within the broader field of biomedical sciences [30,31].

The molecular docking method plays a significant role in the process of drug discovery. This method is instrumental in the identification and optimization of potential drug candidates. Moreover, it provides significant insights into the molecular interactions that underpin diverse biological processes. A fundamental aspect of docking studies is the judicious selection of target molecules, as this has been shown to significantly influence the accuracy and relevance of the ensuing findings [32]. CYP51, an abbreviation for “lanosterol 14α-demethylase,” is categorized as a cytochrome P450 enzyme, a superfamily of enzymes that are distributed throughout numerous eukaryotic organisms. In protozoa such as *Acanthamoeba*, it plays a crucial role in sterol biosynthesis and is vital for cell survival, highlighting its potential as a promising drug target against parasitic infections [33]. In protozoa, profilins have been shown to regulate actin dynamics by interacting with phosphoinositides and proline-rich proteins. This interaction serves to link cyto-skeletal remodeling to signaling pathways that are required for transitions between motile and invasive phases. *A. castellanii* expresses multiple profilin isoforms (IA, IB, and II), which represent potential drug targets, as inhibition of profilin-mediated actin regulation can impair motility, feeding, and pathogenicity [34]. Accordingly, in the present study, *A. castellanii* CYP51, *A. castellanii* profilin IA, IB, and II were utilized as target molecules.

**Figure 1:**
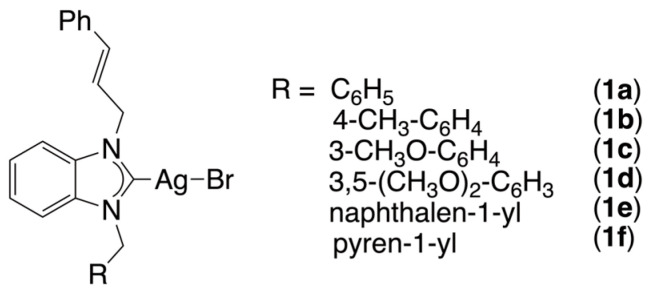
Silver complexes **1a**–**f** studied in the present study against *A. castellanii* and *A. hatchetti*.

A review of recent literature reveals studies exploring the activity of imidazolium salts or silver(I)-*N*-heterocyclic carbene complexes against *Acanthamoeba* [35,36]. This observation suggests the potential for activity of complexes such as those described in this study, specifically bromo(1-alkyl-3-cinnamyl-benzimidazol-2-ylidene)silver (I) (**1a**–**f**) with benzyl (**1a**), 4-methylbenzyl (**1b**), 3-methoxylbenzyl (**1c**), 3,5-dimethoxy-benzyl (**1d**), naphthalen-1-ylmethyl (**1e**) pyren-1-ylmethyl (**1f**) (Figure 1), against in vitro trophozoites of two *Acanthamoeba* isolates of clinical significance: *A. castellanii* (genotype T4) and *A. hatchetti* (genotype T6), both of which were obtained from cases of severe keratitis. In addition to assessing trophozoite viability, we investigated morphological alterations and adhesion capacity specifically, as these processes are critical steps in the pathogenesis of AK. Finally, the silver(I) complexes and their benzimidazolium salts precursors were evaluated in silico for their interactions against *A. castellanii* CYP51, *A. castellanii* profilin IA, IB, and II by the molecular docking method.

## 2. Results and Discussion

### 2.1. Synthesis and Characterization of the Silver(I) Complexes

**Scheme 1:**
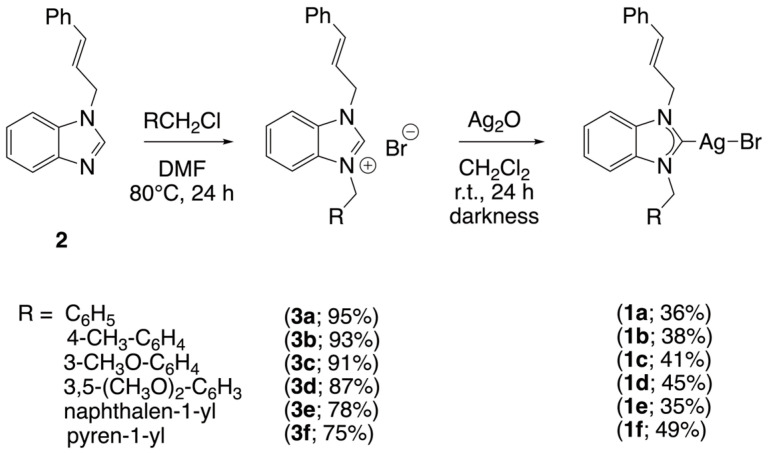
Synthesis of silver(I) complexes **1a**–**f** from 1-cinnamyl-benzimidazole (**2**).

The synthesis of six silver(I) complexes **1a**–**f** was accomplished in two steps from 1-cinnamyl-benzimidazole (**2**). Initially, the benzimidazolium salts **3a**–**f** were prepared by alkylation of 1-cinnamyl-benzimidazole (**2**) and aryl bromide at 80 °C in DMF for 24 h. Following the precipitation process, salts **3a**–**f** were isolated, with yields ranging from 35 to 49%. The silver complexes were subsequently obtained by reacting these salts with silver oxide (Ag_2_O) in dichloromethane for a period of 24 h at room temperature in the dark (Scheme 1).

The characterization of benzimidazolium salts **3a**–**f** and silver(I) complexes **1a**–**f** was accomplished through the utilization of Fourier transform infrared spectroscopy (FT-IR), nuclear magnetic resonance spectroscopy (^1^H and ^13^C NMR), elemental analysis and mass spectrometry (see Section 3 and Supplementary Materials). The silver(I) complexes exhibited sensitivity to light. However, in the absence of light, these samples demonstrated stability within a dimethyl sulfoxide (DMSO-*d*_6_) solution for a period of at least three days.

As previously observed during the synthesis of Ag(NHC) complexes [35,37], a downshift was detected for the ν(CN) bands in the FT-IR spectra between benzimidazolium salts **3a**–**f** (between 1551 and 1558 cm^−1^) and the silver(I) complexes **3a**–**f** (between 1386 and 1396 cm^−1^). The observed change in stretching frequency can be attributed to a weakening of the carbon-nitrogen bond, which becomes a partial double bond after coordination, as supported by the X-ray structure of complex **1b** (vide infra).

**Figure 2:**
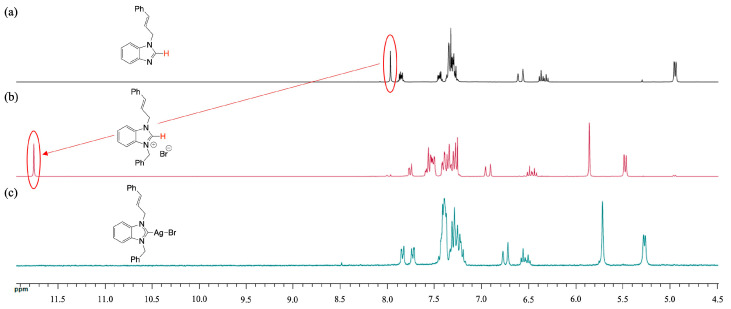
^1^H NMR spectra of (**a**) 1-cinnamyl-benzimidazole (**2**), (**b**) 1-benzyl-3-cinnamyl-benzimidazolium bromide (**3a**) and (**c**) bromo(1-benzyl-3-cinnamyl-benzimidazol-2-ylidene)silver (I) (**1a**).

An analysis of the ^1^H NMR spectra of the silver(I) complexes **1a**–**f** revealed the disappearance of the downfield-shifted signal corresponding to the acidic protons (NCHN) of the corresponding imidazolium salts **3a**–**f** (*δ =* 11.59–11.80 ppm). The latter underwent significant deshielding after alkylation of 1-cinnamyl-benzimidazole (**2**) (*δ =* 7.97 ppm; Figure 2).

When observed, analysis of the complex ^13^C{^1^H} NMR spectra revealed the presence of NC(Ag)N carbon peaks at 190.43 and 198.73 ppm for complexes **1c** and **1f**, respectively [38], compared to 143.34 and 143.50 ppm for benzimidazolium salts **3c** and **3f**, respectively, before coordination (NCHN). During the coordination step, the *trans* conformation of the carbon-carbon double bond of the cinnamyl substituent remains unchanged, with the coupling constant between the two protons of the double bond being approximately 16 Hz.

The mass spectra of silver(I) complexes exhibit a peak that corresponds to the cations [M − Br]^+^ at *m*/*z* = 491.09 for complex **1d** and [M − Br + CH_3_CN]^+^ at *m*/*z* = 472.10, 486.11, 502.11 and 522.11 for complexes **1a**, **1b**, **1c** and **1e**, respectively, with the expected isotopic profiles. Furthermore, the six spectra exhibited molecular ion peaks corresponding to the cations [Ag(NHC)_2_]^+^ at *m*/*z* = 755.23, 783.26, 815.24, 815.24, 855.26 and 1003.29 for complexes **1a**–**f**, respectively, with the expected isotopic profiles. The observation of these bis-carbene complexes [Ag(NHC)_2_]^+^, generated under the sampling conditions employed for ESI-TOF [39], demonstrates the lability (lower Ag-C bond) of the carbene fragments. This lability is responsible for the reported equilibrium between the complexes [AgX(NHC)] and [Ag(NHC)_2_]^+^ [AgX_2_]^−^ [40,41]. In solution, the formation of the latter complexes has not been observed.

**Figure 3:**
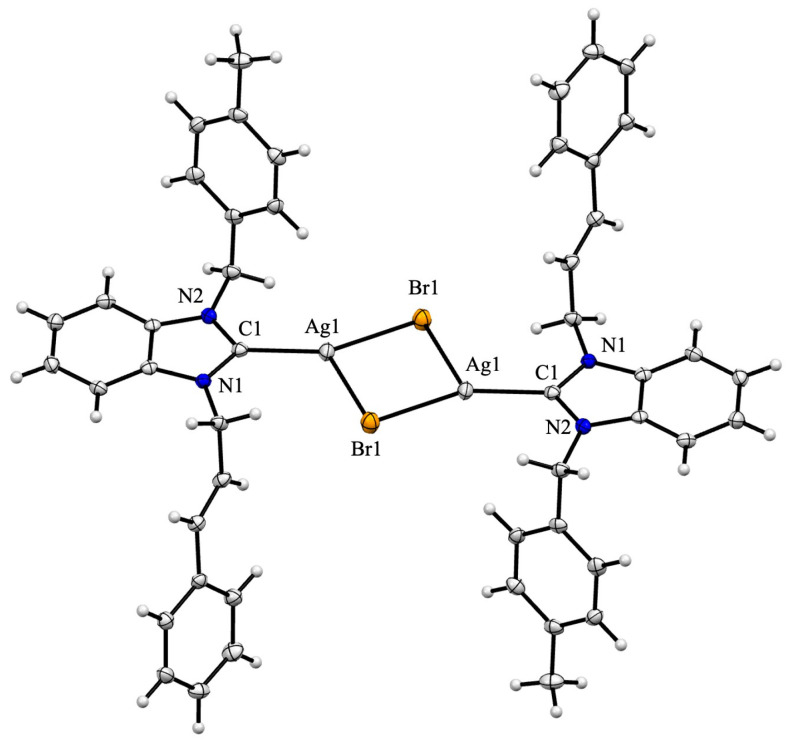
The ORTEP drawing of silver(I) complex **1b**, with 50% probability thermal ellipsoid, shows the atom labeling. Important bond lengths (Å) and angles (°): Ag1-Br1 2.5274(5), Ag1-Br1 2.8500(5), Ag1-C1 2.111(3), N1-C1 1.357(4), N1-C2, 1.359(4), C1-Ag1-Br1 153.40(9), Br1-Ag1-Br1 91.943(15), Br1-Ag1-C1 114.50(9), N1-C1-Ag1 127.9(2), Ag1-C1-N2 126.2(2) and N2-C1-N1 105.9(3).

The formation of silver(I) complexes was confirmed by an X-ray diffraction study on single crystals of complex **1b**, which crystallizes as a centrosymmetric dimer in the triclinic space group *P*$\bar{1}$, with the inversion center located at the midpoint of the [Ag_2_Br_2_] bridge core with Ag-Br bonds of 2.5274(5) and 2.8500(5) Å (Figure 3). Note that dimer formation is frequently observed during the crystallization of (NHC)Ag-X (X = Cl or Br) complexes [35,39]. The Ag1-Br1 and Ag1-C1 distances of 2.5274(5) and 2.111(3) Å, respectively, are within the usual range [42]. Due to dimer formation of the complex, the C-Ag-Br alignment is angled (C1-Ag1-Br1 = 153.40(9). The [Ag_2_Br_2_] moiety is also out of the plane of the parallel benzimidazole rings, resulting in a dihedral angle of 5.74°. The two aromatic rings corresponding to the cinnamyl and 4-methylbenzyl substituents are nearly perpendicular to each other, forming a dihedral angle of 83.46°, and are located on opposite sides of the plane containing the benzimidazole ring. The dihedral angles measured between the benzimidazole ring and the phenyl and the 4-methylphenyl rings are 72.04° and 73.99°, respectively.

### 2.2. In Vitro Effects on Acanthamoeba

#### 2.2.1. Effect of Benzimidazolium Salts and Silver(I) Complexes on *Acanthamoeba* Trophozoites

The in vitro activities of benzimidazolium salts **3a**–**f** and silver(I) complexes **1a**–**f** against trophozoites of *A*. *castellanii* and *A*. *hatchetti* at concentrations of 10, 100, and 1000 µM after 24, 48, and 72 h of exposure were evaluated.

**Figure 4:**
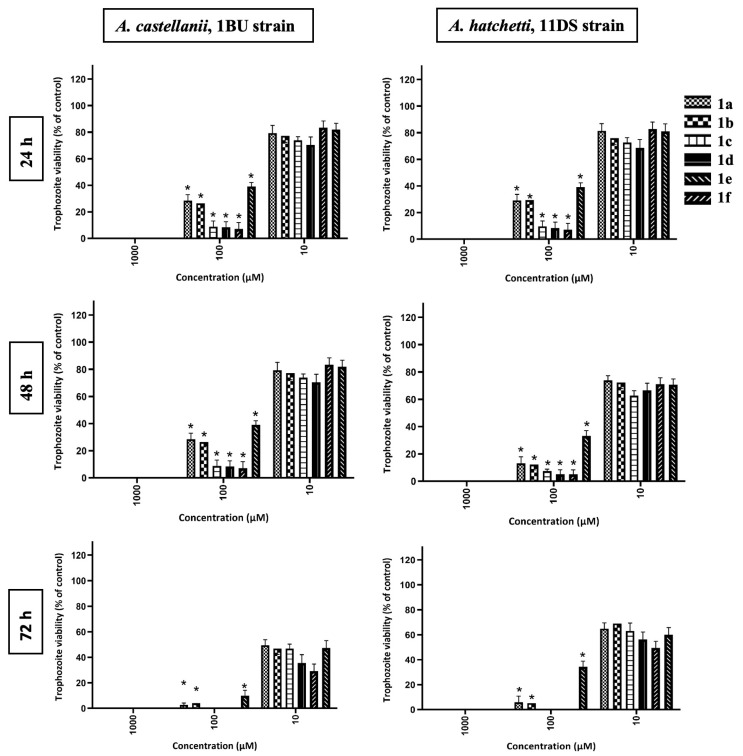
Viability of *A*. *castellanii* and *A*. *hatchetti* trophozoites following exposure to silver(I) complexes **1a**–**f** at concentrations of 1000 µM, 100 µM and 10 µM for 24, 48 and 72 h. Data are shown as the mean ± standard deviation from four independent experiments (* *p* < 0.05 vs. untreated control).

All inorganic complexes **1a**–**f** exhibited potent amoebicidal activity in a dose- and time-dependent manner. Complete inhibition of trophozoite growth was observed at 1000 µM within 24 h in both strains. At 100 µM, complexes **1c**, **3d**, and **3e** rapidly and markedly reduced viability, resulting in survival rates of less than 10% after 48 h for both *A*. *castellanii* and *A*. *hatchetti*. At the lowest concentration (10 µM), the complexes only partially inhibited growth; trophozoite viability remained above 50% in both bacteria (Figure 4).

The two strains exhibited analogous sensitivity patterns. *A*. *castellanii* exhibited a marginal tendency towards elevated susceptibility in comparison to *A*. *hatchetti*; however, this discrepancy did not attain statistical significance (*p* > 0.05). These results suggest that sequence type T4 may be slightly more vulnerable to silver(I) complexes than sequence type T6. The findings demonstrate that silver(I) complexes **1a**–**f** exhibit potent amoebicidal activity against pathogenic *Acanthamoeba* trophozoites. The efficacies of these complexes are determined by the structure, concentration, and exposure time of the inorganic drugs.

**Figure 5:**
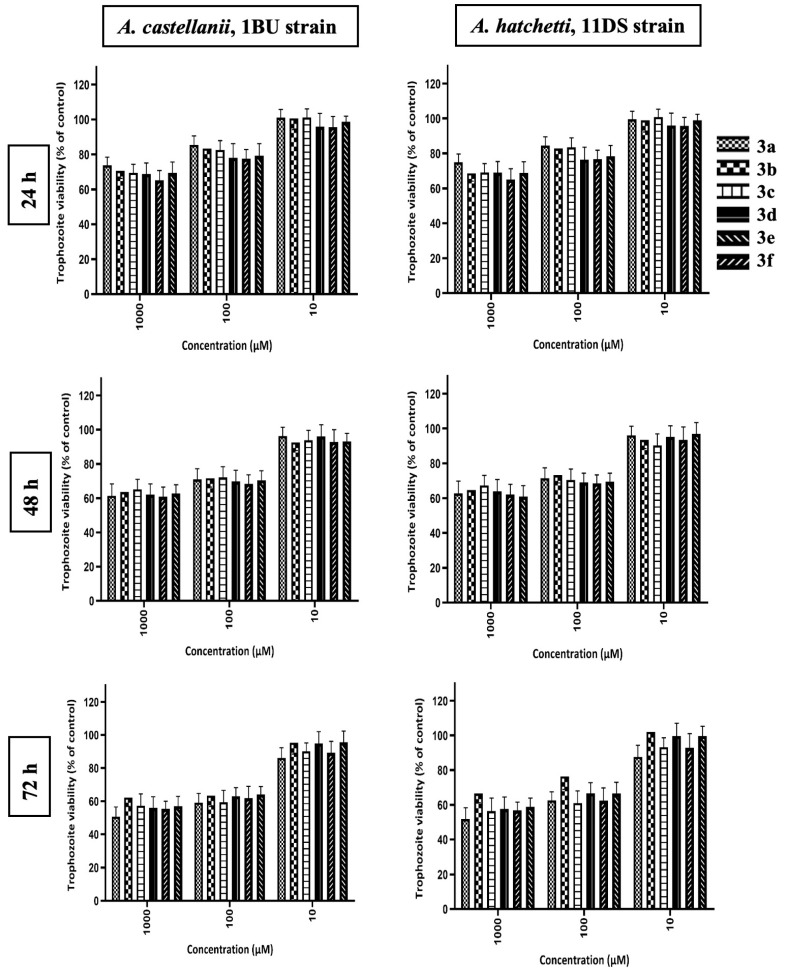
The viability of trophozoites of *A*. *castellanii* and *A*. *hatchetti* was assessed following exposure to benzimidazolium salts **3a**–**f** at concentrations of 1000, 100 and 10 µM for 24, 48 and 72 h. Data are shown as the mean ± standard deviation from four independent experiments. No significant differences were observed compared to the untreated controls (*p* > 0.05).

The benzimidazolium salts **3a**–**f** exhibited minimal to no inhibitory effect on *Acanthamoeba* trophozoites. As anticipated, the lowest trophozoite viability, approximately 60%, was observed after 72 h at concentrations of 1000 µM (Figure 5). These results suggest that the ligands alone possess minimal amoebicidal activity, indicating that the observed anti-amoebic effects are attributable to the addition of the silver atom. This finding underscores the critical role of silver coordination in mediating activity against the pathogenic *Acanthamoeba* trophozoites.

#### 2.2.2. Effect of Silver(I)-NHC Complexes on Trophozoite Morphology and Adhesion Capacity

**Figure 6:**
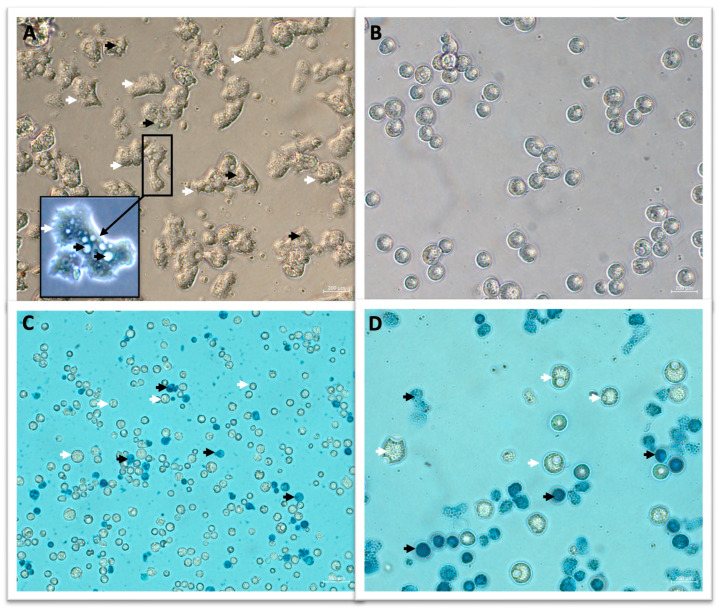
Representative micrographs showing *Acanthamoeba* trophozoites before and after exposure to silver(I) complexes. (**A**) Untreated trophozoites displaying an irregular shape, acanthopodia (white arrows) and contractile vacuoles (black arrows). (**B**) Trophozoites one hour after treatment, showing loss of acanthopodia, rounding and detachment from the substrate. (**C**,**D**) Methylene blue-stained trophozoites at 20× (**C**) and 40× (**D**) magnification. (Scale bar = 200 µm).

Within one hour of exposure to silver(I) complexes **1a**–**f**, *Acanthamoeba* trophozoites exhibited distinct morphological changes, including the loss of acanthopodia, rounding, and detachment from the culture surface (Figure 6A,B). Before treatment, the trophozoites exhibited their characteristic irregular shape, with prominent acanthopodia and visible contractile vacuoles (Figure 6A).

Following a one-hour incubation period with silver(I) complexes, the trophozoites exhibited a rounded morphology and became detached from the substrate (Figure 6B). The viability assessment by methylene blue staining corroborated these observations (Figure 6C,D). The presence of live trophozoites was identified through their unstained cell characteristics and subsequent differentiation from non-viable, blue-stained trophozoites. At one hour post-treatment, the majority of rounded and detached trophozoites remained viable, indicating that the morphological alterations represented an early, potentially reversible stress response rather than immediate cell death. These findings suggest that silver complexes **1a**–**f** initially impair adhesion and cytoskeletal integrity before exerting their full amoebicidal effect at later time points. These morphological alterations were observed in both *A*. *castellanii* and *A*. *hatchetti*, and were consistently induced by all silver(I) complexes **1a**–**f** that were tested.

Note that, in contrast to the pronounced effects observed with silver(I) complexes, neither AgNO_3_ nor Ag_2_O exhibited significant amoebicidal activity against *Acanthamoeba* trophozoites. In all concentrations and incubation times examined, the viability of trophozoites in cultures treated with AgNO_3_ and Ag_2_O was equivalent to that of the untreated control groups (*p* > 0.05). Microscopic examination confirmed that trophozoites in these groups retained their typical morphology, with intact acanthopodia and substrate adherence, throughout the observation period. The results of this study indicate that the amoebicidal and anti-adhesion effects observed are unique to silver(I) complexes **1a**–**f** and cannot be attributed solely to the silver atom.

The early loss of *Acanthopodia* and detachment are of critical clinical relevance. These structures are critical for *Acanthamoeba* to adhere to contact lenses and the corneal epithelium, which is an initial and pivotal step in the pathogenesis of contact-lens-associated AK [43,44]. It has been posited that by impairing adhesion before cell death, silver(I) complexes **1a**–**f** could reduce virulence and prevent the colonization of ocular surfaces. The distinguishing characteristics of silver(I) complexes **1a**–**f**, which set them apart from conventional silver compounds or ligands, are their dual mechanism, an early anti-adhesion phase followed by a delayed amoebicidal activity. This dual mechanism underscores the potential therapeutic advantage of silver(I) complexes **1a**–**f**. This phenomenon is of particular concern to contact lens users, as AK predominantly afflicts this demographic. Adhesion to lens surfaces serves as a portal for infection, and multipurpose solutions frequently prove ineffective in preventing *Acanthamoeba* attachment in its entirety [45,46]. Research on silver nanoparticles has already demonstrated a reduction in trophozoite adhesion to lens materials [47].

### 2.3. Molecular Docking

Cytochrome P450 enzymes (CYPs) are a large superfamily of heme-containing enzymes that are found in nearly all living organisms. CYPs are not only responsible for phase I drug metabolism but also metabolize hormones, fatty acids, cholesterol, steroids, and vitamins. CYP51, an enzyme classified as a lanosterol 14*α*-demethylase, belongs to the CYP enzyme superfamily, a diverse group of enzymes found in nearly all eukaryotes [48]. In humans and animals, CYP51 is imperative for the production of cholesterol, a vital component of cell membranes, hormones, and signaling molecules. It is also essential for the synthesis of ergosterol, the fungal equivalent of cholesterol. In protozoa such as *Acanthamoeba*, CYP51 plays an important role in sterol synthesis and is essential for survival, making it a potential drug target for parasitic infections [49]. Salem and co-workers conducted a study to analyze the inhibition activity of the compounds of Padina pavonica alcoholic extract against *A. castellanii* CYP51. They recorded the best binding affinity as −8.25 kcal/mol for 6,8-Di-C-á-Glucosylluteolin, which exhibited hydrophobic interactions with Met471 and Phe365 at the active site [50]. Soliman and co-workers evaluated the anti-amoebic activity of several triazole compounds by employing the molecular docking method against *A. castellanii* CYP51. The most stable tested compound, 4-(3-bromo-phenyl)-3-(4-isopropyl-phenyl)-5-(3-trifluoromethyl-benzylsulfanyl)-4H[1,2,4]triazole, exhibited an aromatic H-bond with Ala290 and π-interactions with Tyr114 and Phe116 [51]. In a separate study, Kurian and co-worker utilized molecular docking to analyze the binding potential of seven distinct heterocyclic compounds against a singular target molecule. Their findings indicated an estimated binding energy of −10.3 kcal/mol for anthrimide [52]. In a recent study, Tüzün and co-workers analyzed the amoebicidal activity of some palmitic acid esters and determined the optimal binding affinity for 9-octadecenoic acid with a hydrogen bond to Phe84 [53].

In this study, the benzimidazolium salts **3a**–**f** and the silver(I) complexes **1a**–**f** were evaluated by the molecular docking method against *A. castellanii* CYP51 (Table 1). As indicated by the reference isavuconazole molecule and previous studies, both the pre-ligands and the complexes interacted with the same residue of the biomacromolecule.

**Figure 7:**
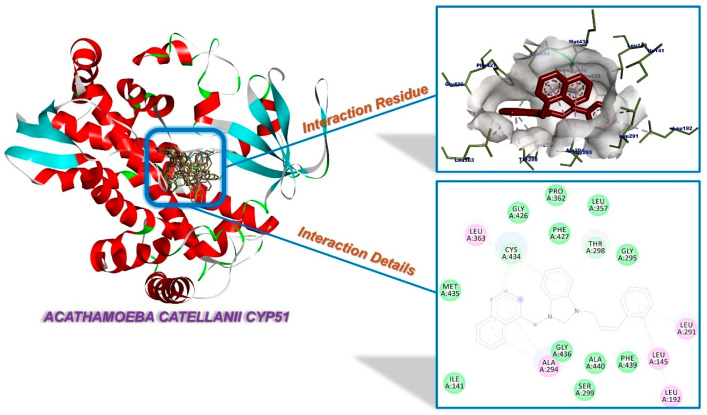
Interaction detail and interaction residue of complex **1f** against *A. castellanii* CYP51.

The silver (I) complexes **1a**–**f** exhibit stronger interactions in comparison to the benzimidazolium salts **3a**–**f** ligand molecule. The strongest binding affinity was observed for complex **1f**, with a recorded value of −11.26 kcal/mol (Figure 7). This molecule has been found to interact with Ala294 and Thr29 through π-interactions, as well as with Leu145 and Leu291 through alkylic interactions. Additionally, numerous van der Waals interactions have been identified. The optimal binding affinity among the pre-ligands was ascertained for **3f**, which exhibited a binding energy of −10.56 kcal/mol. The salt **3f** demonstrated H-bonding with Thr298 and Cys434, in addition to a π-interaction with Phe427, along with a multitude of alkylic and van der Waals interactions. Furthermore, the complex **1e** and its pre-ligand **3e** exhibit noteworthy binding affinities of −9.85 kcal/mol and −9.30 kcal/mol, respectively. The results of the study indicated that the methoxy-substituted ligand exhibited a higher degree of efficacy in comparison with the methyl-substituted ligand. Furthermore, the presence of bulky naphthalene and anthracene moieties led to an enhancement in the activity of both the ligands and the complexes, surpassing the activity of the methoxy-substituted ligand. The complete set of interaction details is presented in the Supplementary Materials.

Profilin, a small actin-binding protein, is found in all eukaryotes and plays a central role in regulating the actin cytoskeleton. This protein is essential for numerous cellular processes, including actin remodeling-dependent cell motility, endocytosis/exocytosis, cytokinesis and axon growth. Consequently, it is associated with various diseases, including cancer and neurodegenerative disorders [54]. Profilin is found not only in higher eukaryotes but also in protozoans such as *Acanthamoeba*. It plays a vital role in the regulation of the protozoan actin cytoskeleton, which is important for phagocytosis, endocytosis, and host invasion. Protozoan profilins couple actin dynamics to signaling cascades by interacting with phosphoinositides and proline-rich proteins. This signaling is required for the transition between motile and invasive phases. *A. castellanii* possesses multiple profilin isoforms, including IA, IB and II. These isoforms have been identified as potential drug targets due to their role in profilin-mediated actin regulation. Inhibition of profilin-mediated actin regulation has been shown to reduce amoeba motility, feeding, and pathogenicity [55]. In their investigation, Khairul, Hashim and co-workers utilized molecular docking to evaluate the interaction of two ethynyl-based chalcone molecules with *A. castellanii* profilin IB. Their findings revealed the formation of H-bonds between 3-(napthalen-1-yl)-1-[4-(4-(cyanophenyl)ethynyl)phenyl]-2-propen-1-one and Lys115, Tyr78 and Asp118, while the drug did not exhibit any H-bond interactions [56]. In their study, Asari and co-workers utilized molecular docking to explore the potential anti-amoebic properties of eugenol derivatives, ultimately identifying the 4-allyl-2-methoxyphenyl 3,4-dichlorobenzoate derivative as the most promising compound. This molecule demonstrated a binding affinity of −7.01 kcal/mol, exhibiting significant interactions with Leu70, Tyr78, Tyr100, Pro106, Gly107, Ala110, Asn111 and Lys115 residues [57]. In a recent study, Kikowska and co-workers evaluated the anti-*Acanthamoeba* activity of the chemical contents of Eryngium planum, Lychnis floscuculi, and Kickxia elatine, and obtained details for five active molecules by the molecular docking method. The binding affinity for chlorogenic acid and rosmarinic acid was determined to be −4.58 kcal/mol, with the interaction occurring through amino acids Leu70, Arg71, Tyr78, Tyr100, Asn111 and Pro106 [58].

In the present study, the molecules **1a**–**f** and **3a**–**f** were evaluated by the molecular docking method against *A. castellanii* profilin IA (Table 2). As indicated by the extant literature, the interaction of the pre-ligands and inorganic complexes with the same biomacromolecule residue was observed. It has been demonstrated that silver(I) complexes **1a**–**f** exhibit stronger interactions in comparison to the benzimidazolium salts **3a**–**f**.

**Figure 8:**
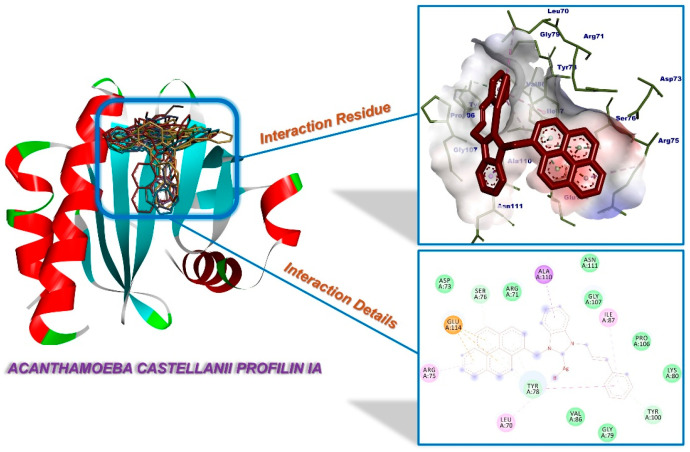
Interaction detail and interaction residue of complex **1f** against *A. castellanii* profilin IA.

The strongest binding affinity was observed for complex **1f**, with a recorded value of −7.48 kcal/mol. This molecule has been found to form H-bonds with Ser76, Tyr78 and Tyr100, as well as π-interactions with Ala110 and Glu114. Additionally, it has been observed to engage in alkylic interactions with Leu70, Arg75 and Ile87, along with numerous van der Waals interactions (Figure 8). The most optimal binding affinity among the pre-ligands was ascertained for salt **3e**, which exhibited a binding energy of −7.24 kcal/mol. The compound **3e** demonstrated the formation of H-bonds with Ser76 and Tyr78, π-interactions with Ala110 and Glu114, and a multitude of alkylic and van der Waals interactions. The results indicate that the incorporation of bulky naphthalene and anthracene moieties into the ligands and complexes enhances their activity compared to other moieties. The complete set of interaction details is presented in the Supplementary Materials.

**Figure 9:**
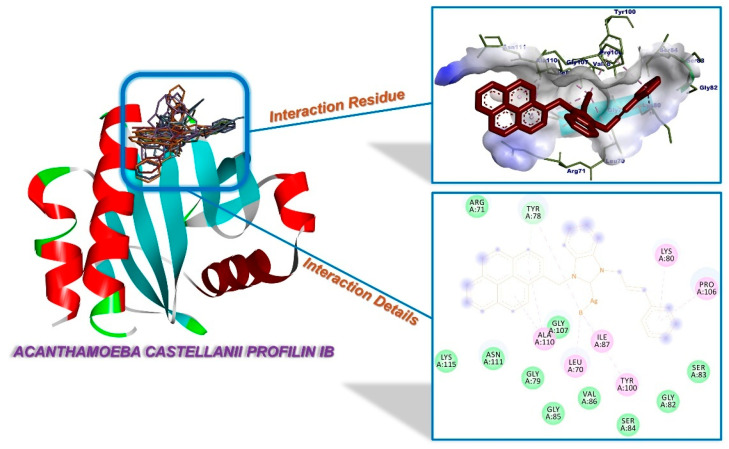
Interaction detail and interaction residue of complex **1f** against *A. castellanii* profilin IB.

**Figure 10:**
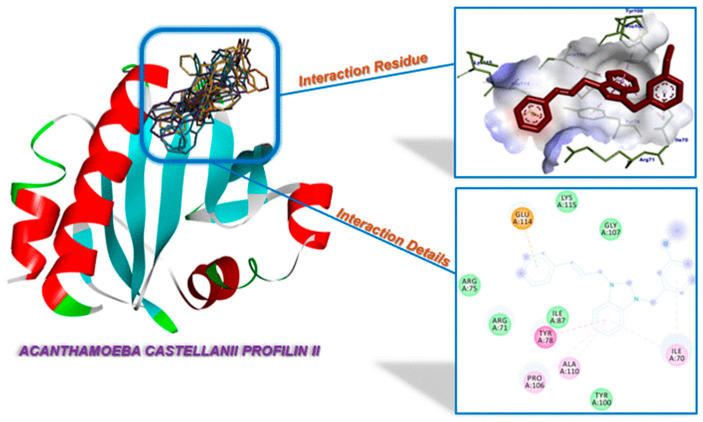
Interaction detail and interaction residue of benzimidazolium salt **3c** against *A. castellanii* profilin II.

Furthermore, the twelve molecules were analyzed against *A. castellanii* profilin IB (Table 3). Both the pre-ligands **3a**–**f** and the complexes **1a**–**f** interacted with the same residue of the biomacromolecule. The salt **3b** demonstrated the strongest binding affinity of the compounds under investigation, with a binding energy of −6.37 kcal/mol. This compound formed a hydrogen bond with Tyr78, a π-interaction with Ala110 and a multitude of alkylic and van der Waals interactions. The silver(I) complex **1f** also exhibited a notable binding affinity of −6.31 kcal/mol (Figure 9). It has been determined that both drugs **1d** and **3a** contain four H-bonds, while **1c** has been found to contain three H-bonds. Furthermore, the silver(I) complexes **1a–f** benzimidazolium salts **3a–f** were analyzed against *A. castellanii* profilin II (Table 4). The interaction of the pre-ligands **3a**–**f** and complexes **3a**–**f** with the residue of the biomacromolecule was found to be consistent. While salt **3c** displayed the strongest binding affinity of −5.22 kcal/mol (Figure 10), it was observed that all molecules exhibited weak interactions. The complete set of interaction details is presented in the Supplementary Materials.

### 2.4. Consideration of Effectiveness Against Bacteria A. castellanii

In vitro tests have unequivocally demonstrated the superiority of silver(I) complexes **1a**–**f** over benzimidazolium salts **3a**–**f** against *A. castellanii* and *A. hatchetti*. As illustrated in Figure 4, the efficacy of silver(I) complexes against the two bacterial strains exhibits a variation in the following sequence: **1e** > **1c**~**1d** >> **1b** > **1a** > **1f**. It is important to note that the alkyl substituent grafted onto the cinnamyl-benzimidazol-2-ylidene has no influence on the σ donor properties of the *N*-heterocyclic carbene ligands. A thorough examination of the ^1^H spectra of the five imidazolium salts **3a**–**f** was conducted, revealing no substantial disparities among the ^1^*J*_HC_ constants (219.4–220.0 Hz) [59]. Therefore, the discrepancies in activity among the five silver complexes are attributable to the nature of the alkyl substituent with the order naphthalen-1-ylmethyl > 3-methoxylbenzyl~3,5-dimethoxy-benzyl >> 4-methylbenzyl > benzyl > pyren-1-ylmethyl. Except for complex **1f**, which carries the pyren-1-ylmethyl substituent, this sequence can be superimposed on the affinity constants calculated in silico from the interactions between inorganic complexes **1a**–**e** and A. *castellanii* CYP51, profilin IA, IB, and II. With the exclusion of the molecular docking results obtained for A. *castellanii* profilin II, which has the lowest affinity constants, the results obtained in vitro and in silico are consistent with the lipophilicity of silver(I) complexes (Table 5) [60,61]. It is important to consider that lipophilicity is not the sole determining factor in assessing the relative efficacy of the pyrenyl complex **1f** against *Acanthamoeba* [62]. In order to interpret the in vitro results obtained with complex **1f**, it is necessary to take other parameters, such as molecular flexibility, which in this case makes diffusion within the bacterium more difficult, as well as solubility in water or topological polar surface area, into account.

## 3. Materials and Methods

### 3.1. General

All manipulations were carried out under dry argon. The ^1^H and ^13^C{^1^H} spectra were recorded using Bruker FT instruments (AC 300 and 500). The calibration of the NMR spectra was conducted in accordance with the residual protonated solvent for CDCl_3_ *δ* = 7.26 ppm and 77.16 ppm for ^1^H and ^13^C{^1^H} NMR, respectively, and for DMSO-*d*_6_ *δ* = 2.50 ppm and 39.52 ppm for ^1^H and ^13^C{^1^H} NMR, respectively. The chemical shifts and coupling constants are reported in ppm and Hz, respectively. ESI-TOF spectra were recorded on a Bruker MicroTOF spectrometer. The infrared spectra were recorded on a Bruker FT-IR Alpha-P spectrometer. The preparation of 1-cinnamyl-benzimidazole (**2**) was conducted in accordance with the established procedure delineated in the extant literature [64].

### 3.2. General Procedure for the Synthesis of Benzimidazolium Salts

The first step in the synthesis process was the dissolution of 1-cinnamyl-benzimidazole **1** (234 mg, 1 mmol) and aryl bromide (1 mmol) in DMF (5 mL). The resulting solution was subjected to a heating process at a temperature of 80 °C for a duration of 24 h. Following a period of cooling to room temperature, the salt underwent precipitation through the addition of Et_2_O (100 mL). The white solid was filtered, washed with Et_2_O (2 × 10 mL), and then dried under vacuum.

#### 3.2.1. 1-Benzyl-3-cinnamyl-benzimidazolium Bromide (**3a**)

Yield: 95%; FT-IR: *ν*(CN) 1558 cm^−1^; ^1^H NMR (300 MHz, CDCl_3_): *δ* = 11.76 (s, 1H, NCHN), 7.77–7.74 (m, 1H, arom CH), 7.60–7.50 (m, 5H, arom CH), 7.42–7.27 (m, 8H, arom CH), 6.93 (d, 1H, CH=C*H*Ph, ^3^*J*_HH_ = 15.9 Hz), 6.47 (dt, 1H, C*H*=CHPh, ^3^*J*_HH_ = 15.9 Hz, ^3^*J*_HH_ = 6.6 Hz), 5.86 (s, 2H, NCH_2_Ph), 5.48 (d, 2H, NC*H*_2_CH=CHPh, ^3^*J*_HH_ = 6.6 Hz); ^13^C{^1^H} NMR (126 MHz, CDCl_3_): *δ* = 143.18 (s, NCHN), 137.33 (s, CH=*C*HPh), 135.07, 132.65, 131.63, 131.50, 129.55, 129.45, 129.00, 128.87, 128.51, 127.34, 127.31, 127.13, 113.88, 113.80 (14 s, arom Cs), 120.09 (s, *C*H=CHPh), 51.69 (s, CH_2_Ph), 50.28 (s, *C*H_2_CH=CHPh) ppm. MS (ESI-TOF): *m*/*z* = 325.17 [M − Br]^+^ (expected isotopic profiles). Elemental analysis (%): calcd for C_23_H_21_N_2_Br (405.33): C: 68.15; H: 5.22; N: 6.91; found C: 67.98; H: 5.17; N: 6.84.

#### 3.2.2. 1-(4-Methylbenzyl)-3-cinnamyl-benzimidazolium Bromide (**3b**)

Yield: 93%; FT-IR: *ν*(CN) 1555 cm^−1^; ^1^H NMR (300 MHz, CDCl_3_): *δ* = 11.80 (s, 1H, NCHN), 7.76–7.71 (m, 1H, arom CH), 7.61–7.53 (m, 3H, arom CH), 7.43–7.39 (m, 4H, arom CH), 7.35–7.28 (m, 3H, arom CH), 7.18 (d, 2H, arom CH, ^3^*J*_HH_ = 7.8 Hz), 6.94 (d, 1H, CH=C*H*Ph, ^3^*J*_HH_ = 16.2 Hz), 6.46 (dt, 1H, C*H*=CHPh, ^3^*J*_HH_ = 16.2 Hz, ^3^*J*_HH_ = 6.7 Hz), 5.80 (s, 2H, C*H*_2_C_6_H_4_CH_3_), 5.48 (d, 2H, C*H*_2_CH=CHPh, ^3^*J*_HH_ = 6.7 Hz), 2.32 (s, 3H, C_6_H_4_C*H*_3_); ^13^C{^1^H} NMR (126 MHz, CDCl_3_): *δ* = 143.28 (s, NCHN), 139.58, 135.07, 131.69, 131.51, 130.24, 129.59, 129.05, 128.91, 128.54, 127.31, 127.29, 127.14, 113.92, 113.75 (14 s, arom Cs), 137.38 (s, CH=*C*HPh), 120.11 (s, *C*H=CHPh), 51.60 (s, N*C*H_2_C_6_H_4_CH_3_), 50.29 (s, NCH_2_CH=CHPh), 21.34 (s, C_6_H_4_*C*H_3_) ppm. MS (ESI-TOF): *m*/*z* = 339.18 [M − Br]^+^ (expected isotopic profiles). MS (ESI-TOF): *m*/*z* = 339.18 [M − Br]^+^ (expected isotopic profiles). Elemental analysis (%): calcd for C_24_H_23_N_2_Br (419.36): C: 68.74; H: 5.53; N: 6.68; found C: 68.66; H: 5.48; N: 6.64.

#### 3.2.3. 1-(3-Methoxylbenzyl)-3-cinnamyl-benzimidazolium Bromide (**3c**)

Yield: 91%; FT-IR: *ν*(CN) 1551 cm^−1^; ^1^H NMR (300 MHz, CDCl_3_): *δ* = 11.69 (s, 1H, NCHN), 7.75 (d, 1H, arom CH, ^3^*J*_HH_ = 10.2 Hz), 7.62–7.53 (m, 3H, arom CH), 7.43–7.39 (m, 2H, arom CH), 7.34–7.26 (m, 4H, arom CH), 7.11 (dd, 1H, arom CH, ^4^*J*_HH_ = 2.1 Hz, ^4^*J*_HH_ = 2.1 Hz), 7.05 (d, 1H, arom CH, ^3^*J*_HH_ = 7.5 Hz), 6.94 (d, 1H, CH=C*H*Ph, ^3^*J*_HH_ = 15.9 Hz), 6.89–6.85 (m, 1H, arom CH), 6.47 (dt, 1H, C*H*=CHPh, ^3^*J*_HH_ = 15.9 Hz, ^3^*J*_HH_ = 6.6 Hz), 5.82 (s, 2H, C*H*_2_C_6_H_4_OCH_3_), 5.48 (d, 2H, C*H*_2_CH=CHPh, ^3^*J*_HH_ = 6.6 Hz), 3.81 (s, 3H, C_6_H_4_OC*H*_3_); ^13^C{^1^H} NMR (126 MHz, CDCl_3_): *δ* = 160.48 (s, arom C_quat_, *C*OCH_3_), 143.34 (s, NCHN), 137.38 (s, CH=*C*HPh), 135.06, 134.07, 131.65, 131.57, 130.60, 129.05, 128.90, 127.38, 127.33, 127.15, 120.56, 125.18, 114.09, 113.91, 113.79 (15 s, arom Cs), 120.09 (s, *C*H=CHPh), 55.89 (s, C_6_H_4_O*C*H_3_), 51.72 (s, N*C*H_2_C_6_H_4_OCH_3_), 50.36 (s, N*C*H_2_CH=CHPh) ppm. MS (ESI-TOF): *m*/*z* = 355.19 [M − Br]^+^ (expected isotopic profiles). Elemental analysis (%): calcd for C_24_H_23_N_2_OBr (435.36): C: 66.21; H: 5.32; N: 6.43; found C: 66.15; H: 5.35; N: 6.34.

#### 3.2.4. 1-(3,5-Dimethoxy-benzyl)-3-cinnamyl-benzimidazolium Bromide (**3d**)

Yield: 87%; FT-IR: *ν*(CN) 1558 cm^−1^; ^1^H NMR (300 MHz, CDCl_3_): *δ* = 11.77 (s, 1H, NCHN), 7.76–7.72 (m, 1H, arom CH), 7.66–7.61 (m, 1H, arom CH), 7.61–7.53 (m, 2H, arom CH), 7.43–7.39 (m, 2H, arom CH), 7.35–7.28 (m, 3H, arom CH), 6.93 (d, 1H, CH=C*H*Ph, ^3^*J*_HH_ = 15.9 Hz), 6.68 (d, 2H, arom CH, ^4^*J*_HH_ = 2.4 Hz), 6.46 (dt, 1H, C*H*=CHPh, ^3^*J*_HH_ = 15.9 Hz, ^3^*J*_HH_ = 6.9 Hz), 6.41 (dd, 1H, arom CH, ^4^*J*_HH_ = 2.4 Hz, ^4^*J*_HH_ = 2.4 Hz), 5.74 (s, 2H, C*H*_2_C_6_H_3_(OCH_3_)_2_), 5.46 (d, 2H, C*H*_2_CH=CHPh, ^3^*J*_HH_ = 6.9 Hz), 3.79 (s, 6H, C_6_H_3_(OC*H*_3_)_2_); ^13^C{^1^H} NMR (126 MHz, CDCl_3_): *δ* = 161.65 (s, arom C_quat_, *C*OCH_3_), 143.31 (s, NCHN), 137.42 (s, CH=*C*HPh), 135.04, 134.74, 131.60, 131.59, 129.07, 128.91, 127.43, 127.35, 127.15, 113.89, 113.73, 106.63, 101.12 (13 s, arom Cs), 120.02 (s, *C*H=CHPh), 55.97 (s, C_6_H_3_(O*C*H_3_)_2_), 51.70 (s, N*C*H_2_C_6_H_3_(OCH_3_)_2_), 50.29 (s, N*C*H_2_CH=CHPh) ppm. MS (ESI-TOF): *m*/*z* = 385.19 [M − Br]^+^ (expected isotopic profiles). Elemental analysis (%): calcd for C_25_H_25_N_2_O_2_Br (465.38): C: 64.52; H: 5.41; N: 6.02; found C: 64.66; H: 5.51; N: 5.87.

#### 3.2.5. 1-(Naphthalen-1-ylmethyl)-3-cinnamyl-benzimidazolium Bromide (**3e**)

Yield: 78%; FT-IR: *ν*(CN) 1557 cm^−1^; ^1^H NMR (300 MHz, CDCl_3_): *δ* = 11.59 (s, 1H, NCHN), 8.18 (d, 1H, arom CH, ^3^*J*_HH_ = 8.4 Hz), 7.90–7.86 (m, 2H, arom CH), 7.76–7.73 (m, 1H, arom CH), 7.5–7.60 (m, 1H, arom CH), 7.57–7.42 (m, 6H, arom CH), 7.40–7.37 (m, 2H, arom CH), 7.33–7.28 (m, 3H, arom CH), 6.90 (d, 1H, CH=C*H*Ph, ^3^*J*_HH_ = 15.9 Hz), 6.47 (dt, 1H, CH=CHPh, ^3^*J*_HH_ = 15.9 Hz, ^3^*J*_HH_ = 6.6 Hz), 6.31 (s, 2H, *C*H_2_C_10_H_7_), 5.48 (d, 2H, *C*H_2_CH=CHPh, ^3^*J*_HH_ = 6.6 Hz); ^13^C{^1^H} NMR (126 MHz, CDCl_3_): *δ* = 143.62 (s, NCHN), 137.23, 134.03, 131.83, 131.64, 130.78, 130.45, 129.37, 128.98, 128.86, 127.94, 127.86, 127.71, 127.36, 127.27, 127.13, 126.73, 122.55, 120.16, 114.01, 113.82 (20 s, arom Cs), 135.08 (s, CH=*C*HPh), 125.52 (s, CH=CHPh), 50.32 (s, N*C*H_2_CH=CHPh), 49.67 (s, N*C*H_2_C_10_H_7_) ppm. MS (ESI-TOF): *m*/*z* = 375.19 [M − Br]^+^ (expected isotopic profiles). Elemental analysis (%): calcd for C_27_H_23_N_2_Br (455.39): C: 71.21; H: 5.09; N: 6.15; found C: 71.26; H: 5.12; N: 6.10.

#### 3.2.6. 1-(Pyren-1-ylmethyl)-3-cinnamyl-benzimidazolium Bromide (**3f**)

Yield: 75%; FT-IR: *ν*(CN) 1556 cm^−1^; ^1^H NMR (500 MHz, CDCl_3_): *δ* = 11.63 (s, 1H, NCHN), 8.41 (d, 1H, arom CH, ^3^*J*_HH_ = 9.0 Hz), 8.18–8.14 (m, 3H, arom CH), 8.07 (s, 2H, arom CH), 8.01–7.92 (m, 2H, arom CH), 7.91 (d, 1H, arom CH, ^3^*J*_HH_ = 9.0 Hz), 7.66 (d, 1H, arom CH, ^3^*J*_HH_ = 8.5 Hz), 7.45–7.41 (m, 1H, arom CH), 7.39–7.34 (m, 3H, arom CH), 7.33–7.30 (m, 1H, arom CH), 7.29–7.26 (m, 1H, arom CH), 7.26–7.23 (m, 2H, arom CH), 6.87 (d, 1H, CH=C*H*Ph, ^3^*J*_HH_ = 15.5 Hz), 6.56 (s, 2H, C*H*_2_C_16_H_9_), 6.48 (dt, 1H, C*H*=CHPh, ^3^*J*_HH_ = 15.5 Hz, ^3^*J*_HH_ = 6.5 Hz), 5.47 (d, 2H, C*H*_2_CH=CHPh, ^3^*J*_HH_ = 6.5 Hz); ^13^C{^1^H} NMR (126 MHz, CDCl_3_): *δ* = 143.50 (s, NCHN), 137.05 (s, CH=*C*HPh), 135.12, 132.13, 131.71, 131.65, 131.17, 130.51, 129.69, 128.93, 128.90, 128.82, 128.44, 127.24, 127.17, 127.11, 127.10, 126.51, 126.15, 126.05, 125.16, 124.92, 124.85, 124.43, 121.60, 114.11, 113.67 (25 s, arom Cs), 120.31 (s, *C*H=CHPh), 50.32 (s, N*C*H_2_CH=CHPh), 49.95 (s, N*C*H_2_C_16_H_9_) ppm. MS (ESI-TOF): *m*/*z* = 449.21 [M − Br]^+^ (expected isotopic profiles). Elemental analysis (%): calcd for C_33_H_25_N_2_Br (529.47): C: 74.86; H: 4.76; N: 5.29; found C: 74.95; H: 4.88; N: 5.18.

### 3.3. General Procedure for the Synthesis of Silver Complexes

In an argon-filled environment, a solution of benzimidazolium salt (0.5 mmol) in CH_2_Cl_2_ (25 mL) was prepared. Subsequent to the incorporation of silver(I) oxide (1.1 mmol), the resultant mixture was subjected to stirring in a dark environment at room temperature for a period of 24 h. Thereafter, the reaction mixture was filtered through a celite bed, and the volume of the resulting solution was reduced to approximately 2.5 mL. The complexes **1a**–**f** were precipitated by the addition of Et_2_O (50 mL), followed by filtration, washing with Et_2_O (2 × 5 mL), and drying under vacuum.

#### 3.3.1. Bromo(1-benzyl-3-cinnamyl-benzimidazol-2-ylidene)silver (I) (**1a**)

Yield: 36%; FT-IR: *ν*(CN) 1392 cm^−1^; ^1^H NMR (300 MHz, DMSO-*d*_6_): *δ* = 7.84 (dd, 1H, arom CH, ^3^*J*_HH_ = 6.9 Hz, ^4^*J*_HH_ = 1.8 Hz), 7.73 (dd, 1H, arom CH, ^3^*J*_HH_ = 6.6 Hz, ^4^*J*_HH_ = 1.8 Hz), 7.46–7.36 (m, 6H, arom CH), 7.34–7.17 (m, 6H, arom CH), 6.75 (d, 1H, CH=*C*HPh, ^3^*J*_HH_ = 16.2 Hz), 6.54 (dt, 1H, CH=CHPh, ^3^*J*_HH_ = 16.2 Hz, ^3^*J*_HH_ = 6.0 Hz), 5.72 (s, 2H, C*H*_2_Ph), 5.27 (d, 2H, C*H*_2_CH=CHPh, ^3^*J*_HH_ = 6.0 Hz); ^13^C{^1^H} NMR (126 MHz, DMSO-*d*_6_): *δ* = 136.33, 133.55, 133.43, 133.35, 128.78, 128.55, 128.03, 128.01, 127.46, 126.52, 124.12, 124.082, 112.38, 112.35 (14 s, arom Cs), 135.63 (s, CH=CHPh), 124.50 (s, CH=CHPh), 51.98 (s, N*C*H_2_Ph), 51.97 (s, N*C*H_2_CH=CHPh) ppm. MS (ESI-TOF): *m*/*z* = 472.10 [M − Br + CH_3_CN]^+^ and 755.23 [M − Br + C_23_H_20_N_2_]^+^ (expected isotopic profiles). Elemental analysis (%): calcd for C_23_H_20_N_2_AgBr (512.19): C: 53.93; H: 3.94; N: 5.47; found C: 54.07; H: 4.03; N: 5.34.Yield: 78%;

#### 3.3.2. Bromo[1-(4-methylbenzyl)-3-cinnamyl-benzimidazol-2-yliden]silver(I) (**1b**)

Yield: 38%; FT-IR: *ν*(CN) 1386 cm^−1^; ^1^H NMR (500 MHz, DMSO-*d*_6_): *δ* = 7.84 (d, 1H, arom CH, ^3^*J*_HH_ = 10.0 Hz), 7.73 (d, 1H, arom CH, ^3^*J*_HH_ = 10.0 Hz), 7.45–7.37 (m, 4H, arom CH), 7.29–7.19 (m, 5H, arom CH), 7.10 (d, 2H, arom CH, ^3^*J*_HH_ = 7.5 Hz), 6.72 (d, 1H, CH=C*H*Ph, ^3^*J*_HH_ = 16.0 Hz), 6.53 (dt, 1H, C*H*=CHPh, ^3^*J*_HH_ = 16.0 Hz, ^3^*J*_HH_ = 6.0 Hz), 5.66 (s, 2H, C*H*_2_C_6_H_4_CH_3_), 5.26 (d, 2H, C*H*_2_CH=CH, ^3^*J*_HH_ = 6.0 Hz), 2.21 (s, 3H, C_6_H_4_C*H*_3_); ^13^C{^1^H} NMR (126 MHz, DMSO-*d*_6_): *δ* = 138.52, 134.60, 134.14, 134.06, 132.11, 129.89, 128.81, 128.53, 127.44, 126.86, 124.35, 123.09, 112.29, 112.01 (14 s, arom Cs), 135.65 (s, CH=*C*HPh), 124.39 (s, *C*H=CHPh), 53.40 (s, N*C*H_2_C_6_H_4_CH_3_), 52.08 (s, N*C*H_2_CH=CHPh), 21.27 (s, C_6_H_4_*C*H_3_) ppm. MS (ESI-TOF): *m*/*z* = 486.11 [M − Br + CH_3_CN]^+^ and 783.26 [M − Br + C_24_H_22_N_2_]^+^ (expected isotopic profiles). Elemental analysis (%): calcd for C_24_H_22_N_2_AgBr (526.22): C: 54.78; H: 4.21; N: 5.32; found C: 54.72; H: 4.16; N: 5.28.

#### 3.3.3. Bromo[1-(3-methoxylbenzyl)-3-cinnamyl-benzimidazol-2-yliden]silver(I) (**1c**)

Yield: 41%; FT-IR: *ν*(CN) 1389 cm^−1^; ^1^H NMR (500 MHz, DMSO-*d*_6_): *δ* = 7.83 (d, 1H, arom CH, ^3^*J*_HH_ = 7.0 Hz), 7.75 (d, 1H, arom CH, ^3^*J*_HH_ = 8.5 Hz), 7.44–7.36 (m, 4H, arom CH), 7.26–7.20 (m, 4H, arom CH), 7.00 (s, 1H, arom CH), 6.92 (d, 1H, arom CH, ^3^*J*_HH_ = 7.5 Hz), 6.84 (d, 1H, arom CH, ^3^*J*_HH_ = 8.0 Hz), 6.71 (d, 1H, CH=C*H*Ph, ^3^*J*_HH_ = 16.0 Hz), 6.53 (dt, 1H, C*H*=CHPh, ^3^*J*_HH_ = 16.0 Hz, ^3^*J*_HH_ = 6.0 Hz), 5.69 (s, 2H, C*H*_2_C_6_H_4_OCH_3_), 5.27 (d, 2H, C*H*_2_CH=CHPh, ^3^*J*_HH_ = 6.0 Hz), 3.68 (s, 3H, C_6_H_4_OC*H*_3_); ^13^C{^1^H} NMR (126 MHz, DMSO-*d*_6_): δ = 190.43 (s, NC(Ag)N), 159.90 (s, arom C_quat_, *C*OCH_3_), 138.35, 133.98, 133.87, 133.82, 130.44, 129.04, 128.49, 126.98, 125.03, 124.60, 114.01, 113.63, 112.84, 112.80, 119.97 (15 s, arom Cs), 136.10 (s, CH=CHPh), 124.55, (s, CH=CHPh), 55.54 (s, C_6_H_4_O*C*H_3_), 52.27 (s, CH_2_CH=CHPh), 51.00 (s, N*C*H_2_C_6_H_4_OCH_3_) ppm. MS (ESI-TOF): *m*/*z* = 502.11 [M − Br + CH_3_CN]^+^ and 815.24 [M − Br + C_24_H_22_N_2_O]^+^ (expected isotopic profiles). Elemental analysis (%): calcd for C_24_H_22_N_2_OAgBr•0.5(C_4_H_10_O) (579.28): C: 52.93; H: 4.80; N: 4.94; found C: 52.95; H: 4.84; N: 4.88.

#### 3.3.4. Bromo[1-(3,5-dimethoxy-benzyl)-3-cinnamyl-benzimidazol-2-ylidene]silver(I) (**1d**)

Yield: 45%; FT-IR: *ν*(CN) 1392 cm^−1^; ^1^H NMR (300 MHz, DMSO-*d*_6_): *δ* = 7.86–7.83 (m, 1H, arom CH), 7.80–7.77 (m, 1H, arom CH), 7.45–7.42 (m, 2H, arom CH), 7.40 (d, 2H, arom CH, ^3^*J*_HH_ = 6.9 Hz), 7.27–7.17 (m, 3H, arom CH), 6.68 (d, 1H, CH=C*H*Ph, ^3^*J*_HH_ = 15.9 Hz), 6.54 (d, 2H, arom CH, ^4^*J*_HH_ = 2.1 Hz), 6.47 (dt, 1H, C*H*=CHPh, ^3^*J*_HH_ = 15.9 Hz, ^3^*J*_HH_ = 6.0 Hz), 6.41 (t, 1H, arom CH, ^4^*J*_HH_ = 2.1 Hz), 5.62 (s, 2H, C*H*_2_C_6_H_3_(OCH_3_)_2_), 5.25 (d, 2H, C*H*_2_CH=CHPh, ^3^*J*_HH_ = 6.0 Hz), 3.65 (s, 6H, C_6_H_3_O(C*H*_3_)_2_); ^13^C{^1^H} NMR (126 MHz, DMSO-*d*_6_): *δ* = 160.70 (s, arom C_quat_, *C*OCH_3_), 138.55, 133.43, 133.28, 128.55, 128.01, 126.47, 124.17, 124.11, 112.36, 112.34, 105.75, 99.16 (12 s, arom Cs), 135.59 (s, CH=CHPh), 124.50 (s, CH=CHPh), 55.16 (s, C_6_H_3_(OC*H*_3_)_2_), 51.80 (s, N*C*H_2_CH=CHPh), 50.52 (s, N*C*H_2_C_6_H_3_(OC*H*_3_)_2_) ppm. MS (ESI-TOF): *m*/*z* = 491.09 [M − Br]^+^ and 815.24 [M − Br + C_25_H_24_N_2_O_2_]^+^ (expected isotopic profiles). Elemental analysis (%): calcd for C_25_H_24_N_2_O_2_AgBr (572.24): C: 52.47; H: 4.23; N: 4.90; found C: 52.42; H: 4.20; N: 4.84.

#### 3.3.5. Bromo[1-(naphthalen-1-ylmethyl)-3-cinnamyl-benzimidazol-2 ylidene]silver(I) (**1e**)

Yield: 35%; FT-IR: *ν*(CN) 1396 cm^−1^; ^1^H NMR (300 MHz, DMSO-*d*_6_): *δ* = 8.05 (d, 1H, arom CH, ^3^*J*_HH_ = 7.8 Hz), 7.99–7.96 (m, 1H, arom CH), 7.86 (d, 2H, arom CH, ^3^*J*_HH_ = 8.4 Hz), 7.74 (d, 1H, arom CH, ^3^*J*_HH_ = 7.5 Hz), 7.58–7.51 (m, 2H, arom CH), 7.49–7.40 (m, 2H, arom CH), 7.34–7.19 (m, 6H, arom CH), 7.00 (d, 1H, arom CH, ^3^*J*_HH_ = 6.9 Hz), 6.54–6.35 (m, 2H, CH=CHPh), 6.05 (s, 2H, C*H*_2_C_10_H_7_), 5.06 (s br, 2H, C*H*_2_CH=CHPh); ^13^C{^1^H} NMR (126 MHz, DMSO-*d*_6_): *δ* = 135.53 (s, CH=*C*HPh), 133.86, 133.46, 133.41, 133.38, 131.85, 130.34, 128.82, 128.60, 128.54, 128.06, 126.78, 126.47, 126.31, 125.52, 124.30, 124.24, 123.17, 112.41, 112.28 (19 s, arom Cs), 124.35 (s, *C*H=CHPh), 50.59 (s, N*C*H_2_CH=CHPh), 49.53 (s, N*C*H_2_C_10_H_7_) ppm. MS (ESI-TOF): *m*/*z* = 522.11 [M − Br + CH_3_CN]^+^ and 855.26 [M − Br + C_27_H_22_N_2_]^+^ expected isotopic profiles). Elemental analysis (%): calcd for C_27_H_22_N_2_AgBr (562.25): C: 57.68; H: 3.94; N: 4.98; found C: 57.79; H: 4.05; N: 4.90.

#### 3.3.6. Bromo[1-(pyren-1-ylmethyl)-3-cinnamyl-benzimidazol-2-yliden]silver(I) (**1f**)

Yield: 49%; FT-IR: *ν*(CN) 1390 cm^−1^; ^1^H NMR (500 MHz, DMSO-*d*_6_): *δ* = 8.28–8.22 (m, 3H, arom CH), 8.18 (d, 1H, arom CH, ^3^*J*_HH_ = 9.5 Hz), 8.10–8.04 (m, 3H, arom CH), 7.99 (d, 1H, arom CH, ^3^*J*_HH_ = 8.5 Hz), 7.78 (d, 1H, arom CH, ^3^*J*_HH_ = 8.0 Hz), 7.69 (d, 1H, arom CH, ^3^*J*_HH_ = 8.0 Hz), 7.63 (d, 1H, arom CH, ^3^*J*_HH_ = 8.0 Hz), 7.46–7.38 (m, 2H, arom CH), 7.16–7.11 (m, 5H, arom CH), 6.37–6.25 (m, 2H, CH=CHPh), 6.12 (s, 2H, CH_2_C_16_H_9_), 4.86 (s br, 2H, C*H*_2_CH=CHPh); ^13^C{^1^H} NMR (126 MHz, DMSO-*d*_6_): *δ* = 198.73 (s, NC(Ag)N), 135.36 (s, CH=*C*HPh), 133.77, 133.25, 133.16, 130.71, 130.67, 130.08, 129.11, 128.41, 128.27, 128.01, 127.92, 127.58, 127.09, 126.50, 126.32, 126.12, 125.73, 125.48, 125.01, 124.19, 124.01, 123.63, 122.44, 112.29, 112.18 (25 s, arom Cs), 124.20 (s, *C*H=CHPh), 50.37 (s, N*C*H_2_CH=CHPh), 49.38 (s, N*C*H_2_C_16_H_9_) ppm. MS (ESI-TOF): *m*/*z* = 1003.29 [M − Br + C_33_H_24_N_2_]^+^ (expected isotopic profiles). Elemental analysis (%): calcd for C_33_H_24_N_2_AgBr (636.33): C: 62.29; H: 3.80; N: 4.40; found C: 62.41; H: 4.05; N: 4.25.

### 3.4. X-Ray Crystallography

The single crystals of silver(I) complex **1b**, suitable for X-ray analysis, were obtained through the slow diffusion of Et_2_O into a CH_2_Cl_2_ solution of the complex. The crystal structure of the complex was determined using Bruker APEX-II CCD and Bruker PHOTON-III CPAD with Mo-K*α* radiation (*λ* = 0.71073 Å). The final structure was elucidated through the utilization of SHELXT-2019/2 [65], a method that revealed the non-hydrogen atoms of the molecule. Subsequent to anisotropic refinement, the location of all hydrogen atoms was ascertained through the use of a Fourier difference map. The structure was refined with SHELXL-2019/3 [66] using the full-matrix least-square technique (use of F square magnitude; x, y, z, and βij for C, Br, N, and Ag atoms; x, y, and z in riding mode for H atoms) (Table 6). The Cambridge Crystallographic Data Centre (CCDC) contains the supplementary crystallographic data for the structures. The data can be obtained free of charge via www.ccdc.cam.ac.uk/structures.

### 3.5. In Vitro Effects on Acanthamoeba Species

#### 3.5.1. *Acanthamoeba* Strains and Culture Conditions

The two employed *Acanthamoeba* strains were isolated from patients afflicted with severe keratitis. The first specimen, *A. castellanii* 1BU, belongs to 18S rDNA sequence type T4, which is the most frequently associated with keratitis. The second strain, designated *A. hatchetti* 11DS, was identified as sequence type T6 [67].

Trophozoites in the exponential growth phase were harvested by centrifugation at 500× *g* for 10 min. Subsequently, the cell pellets were washed twice with a sterile Neff’s saline solution (1.2 g NaCl, 0.4 g MgSO_4_•H_2_O, 0.4 g CaCl_2_•2H_2_O, 1.42 g Na_2_HPO_4_, and 1.36 g KH_2_PO_4_ in 100 mL of distilled water). Cell density was determined by measuring the optical density of the samples using a Neubauer hemocytometer. The samples were then adjusted to contain 1 × 10^4^ trophozoites/mL, with ≥95% viability in PYG medium. The suspensions were utilized promptly for viability assays.

#### 3.5.2. Effect on *Acanthamoeba* Trophozoites

The silver(I) complexes **1a**–**f**, along with their corresponding benzimidazolium salts **3a**–**f**, were prepared using 100% dimethyl sulfoxide (DMSO). The same approach was used for the solutions of silver nitrate (AgNO_3_) and silver oxide (Ag_2_O). Working solutions at final concentrations of 10, 100, and 1000 µM were obtained by diluting the stock solutions with PYG medium. The final concentration of DMSO in all groups, including the highest test concentration, was standardized to 10% (*v*/*v*).

Trophozoites were seeded at a density of 1 × 10^4^ cells per mL per well in sterile 24-well tissue culture plates. Subsequently, the plates were exposed to the test compounds and were subjected to an incubation period at a temperature of 25 °C for a duration of 24, 48, or 72 h. Subsequent to the incubation period, the trophozoites that had become attached to the plate were detached using a sterile cell scraper and resuspended by means of gentle pipetting. The trophozoite suspensions were stained with 0.3% methylene blue for a period of 10 min. The viability of the trophozoites was determined by staining them with methylene blue, a method that distinguishes between viable (unstained) and non-viable (blue-stained) cells. The cell count was performed using a Neubauer hemocytometer under a light microscope. The percentage of viable trophozoites was subsequently calculated relative to the untreated control. The negative control consisted of trophozoites cultured in PYG medium alone, while the solvent control contained 10% (*v*/*v*) DMSO. It is imperative to note that all experiments were performed in quadruplicate.

#### 3.5.3. Microscopic Observation of Trophozoite Morphology

In order to evaluate the early morphological alterations, trophozoites of *A. castellanii* and *A. hatchetti* were incubated with silver(I) complexes **1a**–**f**, benzimidazolium salts **3a**–**f**, AgNO_3_ or Ag_2_O, all at a concentration of 100 µM. This concentration was selected because it reliably induced quantifiable biological effects in viability assays while circumventing the immediate death of trophozoites observed at higher concentrations. The assessment of morphological features was conducted one hour post-treatment using an inverted phase-contrast microscope (Eclipse Ti2, Nikon, Tokyo, Japan). Representative images were captured using an integrated digital camera system. The observations concentrated on the presence or absence of acanthopodia, cell rounding, and detachment from the substrate. Concurrently, the viability of the cells was evaluated using a 0.3% methylene blue exclusion technique.

### 3.6. Statistical Analysis

All experiments were conducted in four independent biological replicates, each of which was performed in technical triplicate. The data are expressed as the mean ± standard deviation (SD). Statistical analyses were performed using GraphPad Prism version 9.0 (GraphPad Software, San Diego, CA, USA). A one-way analysis of variance (ANOVA) was employed to assess the variances among the multiple groups, and Tukey’s post hoc test was subsequently utilized to identify any significant differences. Pairwise comparisons were conducted using a two-tailed Student’s *t*-test. A *p*-value of less than 0.05 was considered statistically significant.

### 3.7. Molecular Docking Method

Before the molecular docking performance, the benzimidazole-type ligands and their Ag(I) complexes were optimized with the ORCA package program, version 4.0. The def2-SVP def2-SVP/J basis sets with BP86 functionals were utilized in conjunction with tightscf and grid4 restrictions [68,69]. The optimized Cartesian coordinates of all molecules are presented in the Supplementary Materials. All molecular docking experiments were conducted using AutodockTools 4.2 [70] against *A. castellanii* CYP51 (pdb: 6ux0) [71], *A. castellanii* profilin IA (pdb:1prq) [72], *A. castellan*ii profilin IB (pdb:1acf) and *A. castellanii* profilin II (pdb:2acg) [73], which were downloaded from the RCSB Protein Data Bank “https://www.rcsb.org/ (accessed on 1 July 2025)”. The target molecules were saved as pdbqt format with Kollman charges, with the polar hydrogens, and without water molecules. The benzimidazole-type molecules and their Ag(I) complexes were also recorded in a database that utilizes the PDBQT format, with the inclusion of Gasteiger charges [74]. The performances were executed using Lamarckian Genetic Algorithms with random positions of the small molecules [75]. The visualizations were prepared using Discovery Studio 4.1.0.

## 4. Conclusions

In summary, the present article describes the synthesis of six bromo[*N*-alkyl-*N*-cinnamyl-benzimidazol-2-ylidene]silver(I) complexes. These complexes were obtained with isolated yields ranging from 35 to 49% from the corresponding benzimidazolium salts in the presence of silver oxide in dichloromethane at room temperature. A combination of analytical techniques, including FT-IR, NMR and X-ray spectroscopic analyses, as well as microanalyses and mass spectrometry analyses, enabled the characterization of these silver complexes.

The in vitro effects of silver(I) complexes on trophozoites from two *Acanthamoeba* isolates obtained from patients with keratitis were studied. The parasites were exposed to increasing concentrations ranging from 10 to 1000 µM for periods ranging from 24 to 72 h. The results of this study demonstrated that silver(I) complexes exhibited dose- and time-dependent activity. A complete inhibition of bacterial growth was observed after 24 h of treatment with a concentration of 1000 µM. At a lower concentration of 100 µM, three complexes containing 3-methoxylbenzyl, 3,5-dimethoxy-benzyl and naphthalen-1-ylmethyl substituents reduced viability to less than 10% after an incubation period ranging from 48 to 72 h. The presence of benzimidazolium salts and simple silver compounds, including Ag_2_O and AgNO_3_, did not cause the loss of acanthopods, rounding or detachment of the bacterial strains.

A computational molecular docking study was conducted on *A. castellanii* CYP51, *A. castellanii* profilin IA, IB, and II. The results indicated that complexes bromo[1-(naphthalen-1-ylmethyl)-3-cinnamyl-benzimidazol-2 ylidene]silver(I) (**1e**) and bromo[1-(pyren-1-ylmethyl)-3-cinnamyl-benzimidazol-2-yliden]silver(I) (**1f**) exhibited the strongest interactions. However, the in vivo results did not align with those observed for the complex with the highest affinity constants (**1f**). This finding indicates that the reactivity of these silver(I) complexes with AK is more intricate than previously assumed, necessitating the consideration of alternative mechanisms of action.

From a therapeutic perspective, compounds capable of preventing adhesion and inducing trophozoite death are highly desirable. The development of silver(I) complexes as prophylactic treatments involves the targeting of the dual pathogenesis routes of AK adhesion and viability. These complexes could be incorporated into contact lens disinfecting solutions or used as topical agents for early-stage AK.

## Figures and Tables

**Table 1:** Interaction details of the molecules against *A. castellanii* CYP51.

Compounds	Binding Affinity (kcal/mol)	Amino Acids Residue *A. castellanii* CYP51
**1a**	−8.36	Thr298 (H-bond), Phe427, Cys434, Met435 (π-interactions), Ile141, Leu291, Ala294, Pro362, Ala440 (alkylic interactions), Leu145, Ala290, Gly295, Ser299, Thr302, Leu357, Leu363, His432, Gly436, Phe439 (van der Waals interactions)
**1b**	−8.37	Thr298, Ser299 (H-bonds), Ala294 (π-interaction), Leu145, Leu291, Leu357, Pro362, Leu363, Ala440 (alkylic interactions), Phe121, Leu192, Phe293, Gly295, Thr302, Gly426, Phe427, Cys434, Gly436, Phe439 (van der Waals interactions)
**1c**	−8.68	Ala294, Cys434, Met435 (π-interactions), Ile141, Ala290, Leu291, Ala440 (alkylic interactions), Leu139, Leu145, Gly295, Thr298, Ser299, Leu363, Phe427, Gly428, His432, Gly433, Gly436, Phe439 (van der Waals interactions)
**1d**	−8.91	Gly433, Cys434, Met435, Gly436 (H-bonds), Phe121, Ala294, Leu363, Ala440 (alkylic interactions), Leu138, Leu291, Phe293, Gly295, Thr298, Ser299, His426, Phe427, Gly428, His432, Phe439 (van der Waals interactions)
**1e**	−9.85	Ala294, Thr298, Cys434, Met435 (H-bonds), Leu145, Leu291 (alkylic interactions), Leu138, Ile141, Leu192, Gly295, Leu357, Pro362, Leu363, Gly426, Phe427, His432, Gly436, Phe439 (van der Waals interactions)
**1f**	−11.26	Ala294, Thr298 (π-interactions), Leu145, Ala290, Leu291, Leu357, Pro362, Leu363, Cys434 (alkylic interactions), Phe121, Val126, Leu192, Phe293, Gly295, His297, Ser299, Gly426, Phe427, Gly436, Phe439, Ala440 (van der Waals interactions)
**3a**	−8.25	Cys434 (π-interaction), Leu145, Leu291, Leu357, Pro362 (alkylic interactions), Leu192, Ala294, Gly295, Thr298, Ser299, Leu363, Gly426, Phe427, Gly428, His432, Gly433, Gly436, Phe439, Ala440 (van der Waals interactions)
**3b**	−8.27	Phe427, Cys434 (π-interactions), Leu145, Leu291, Leu357, Pro362 (alkylic interactions), Leu192, Ala294, Gly295, Thr298, Ser299, Leu363, Gly426, Gly428, Ala429, His532, Gly433, Gly436, Phe439, Ala440 (van der Waals interactions)
**3c**	−8.63	His432, Gly433, Cys434, Met435 (H-bonds), Thr298 (π-interactions), Leu145, Leu291, Pro362 (alkylic interactions), Leu192, Ala294, Gly295, Ser299, Leu357, Leu363, Gly426, Phe427, Gly436, Phe439, Ala440 (van der Waals interactions)
**3d**	−8.67	Thr298, His432, Cys434 (H-bonds), Phe427 (π-interaction), Leu145, Leu291, Leu357, Pro362, Leu363, Ala440 (alkylic interactions), Leu192, Ala294, Gly295, Ser299, Thr302, Gly426, Gly428, Gly433, Gly436 Glu437, Phe439, Ile444 (van der Waals interactions)
**3e**	−9.30	Thr298, Cys434 (H-bonds), Leu145, Leu192, Leu291, Ala294, Leu363 (alkylic interactions), Ile141, Gly295, Ser299, Leu357, Pro362, Gly426, Phe427, Met435, Gly436, Phe439, Ala440 (van der Waals interactions)
**3f**	−10.56	Thr298, Cys434 (H-bonds), Phe427 (π-interaction), Leu145, Leu291, Ala294, Leu357, Pro362, Leu363, Ala440 (alkylic interactions), Leu192, Gly295, Ser299, Thr302, Gly426, Gly428, His432, Gly433, Met435, Gly436, Glu437, Phe439, Ile444 (van de Waals interactions)

**Table 2:** Interaction details of the molecules against *A. castellanii* profilin IA.

Compounds	Binding Affinity (kcal/mol)	Amino Acids Residue *A. castellanii* Profilin IA
**1a**	−6.49	Tyr100 (H-bond), Tyr78 (π-interaction), Arg71, Ile87, Pro106, Ala110 (alkylic interactions), Leu70, Asp73, Ser76, Gly79, Lys80, Gly85, Val86, Gly107, Asn111, Glu114 (van der Waals interactions)
**1b**	−6.32	Arg71, Tyr78, Ala110 (π-interactions), Leu70, Ile87, Tyr100, Pro106 (alkylic interactions), Asp73, Arg75, Ser76, Gly79, Gly85, Val86, Gly107, Asn111, Glu114 (van der Waals interactions)
**1c**	−6.74	Ser76, Tyr78, Tyr100 (H-bonds), Glu114 (π-interaction), Leu70, Arg71, Arg75, Ile87, Pro106, Ala110 (alkylic interactions), Asp73, Gly79, Lys80, Gly85, Val86, Gly107, Ala109 (van der Waals interactions)
**1d**	−6.12	Ser76, Gly79, Val86, Tyr100 (H-bonds), Glu114 (π-interaction), Leu70, Arg71, Tyr78, Pro106, Ala110 (alkylic interactions), Asp73, Arg75, Gly85, Ile87, Gly107 (van der Waals interactions)
**1e**	−7.47	Tyr100 (H-bond), Tyr78 (π-interaction), Arg71, Ile87, Ala110 (alkylic interactions), Leu70, Gly79, Lys79, Lys80, Gly85, Val86, Pro106, Gly107, Glu114 (van der Waals interactions)
**1f**	−7.48	Ser76, Tyr78, Tyr100 (H-bonds), Ala110, Glu114 (π-interactions), Leu70, Arg75, Ile87 (alkylic interactions), Arg71, Asp73, Gly79, Lys80, Gly85, Val86, Pro106, Gly107, Asn111 (van der Waals interactions)
**3a**	−6.24	Ser76, Tyr78, Tyr100 (H-bonds), Glu114 (π-interaction), Ile87, Pro106, Ala110 (alkylic interactions), Leu70, Arg71, Asp73, Arg75, Gly79, Lys80, Gly85, Val86, Gly107 (van der Waals interactions)
**3b**	−6.30	Tyr (H-bond), Tyr78 (π-interactions), Leu70, Arg71, Ile87, Pro106, Ala110 (alkylic interactions), Gly79, Lys80, Gly85, Val86, Gly107, Asn111 (van der Waals interactions)
**3c**	−6.20	Gly79, Val86, Tyr100 (H-bonds), Tyr78, Ala110 (π-interactions), Leu70, Arg71, Pro106 (alkylic interactions), Gly85, Ile87, Gly107, Asn111 (van der Waals interactions)
**3d**	−5.59	Val86, Tyr100 (H-bonds), Ala110 (π-interaction), Leu70 (alkylic interaction), Arg71, Tyr78, Gly79, Lys80, Gly85, Ile87, Gly107, Asn111, Glu114 (van der Waals interactions)
**3e**	−7.24	Ser76, Tyr78 (H-bonds), Ala110, Glu114 (π-interactions), Arg75, Ile87, Pro106 (alkylic interactions), Leu70, Arg71, Asp73, Tyr100, Gly107, Ala109 (van der Waals interactions)
**3f**	−6.85	Tyr100 (H-bond), Arg71, Tyr78, Ala110 (π-interactions), Ile87, Pro106 (alkylic interactions), Leu70, Gly79, Lys80, Gly85, Val86, Gly107, Asn111, Glu114 (van der Waals interactions)

**Table 3:** Interaction details of the molecules against *A. Castellanii* profilin IB.

Compounds	Binding Affinity (kcal/mol)	Amino Acids Residue *A. castellanii* Profilin IB
**1a**	−5.32	Tyr78, Tyr100 (H-bonds), Leu70, Arg71, Ile87, Ala110 (alkylic interactions), Val86, Pro106, Gly107, Ala109, Glu114 (van der Waals interactions)
**1b**	−5.44	Tyr100 (H-bond), Tyr78, Ala110 (π-interactions), Leu70, Arg71, Ile87 (alkylic interactions), Gly79, Gly85, Val86, Pro106, Gly107, Asn111 (van der Waaals interactions)
**1c**	−5.80	Gly79, Val86, Tyr100 (H-bonds), Tyr78, Pro106, Glu114 (π-interaction), Arg71, Arg75, Ala110 (alkylic interactions), Leu70, Arg75, Gly85, Ile87, Gly107, Asn111, Lys115 (van der Waals interactions)
**1d**	−5.60	Leu70, Arg71, Tyr78, Tyr100 (H-bonds), Glu114 (π-interaction), Ile87, Ala110 (alkylic interactions), Gly79, Val86, Pro106, Gly107, Asn111 (van der Waals interactions)
**1e**	−6.24	Leu70, Tyr78, Glu114 (π-interactions), Arg71, Ile87, Pro106, Ala110 (alkylic interactions), Gly79, Lys80, Gly107, Asn111, Lys115 (van der Waals interactions)
**1f**	−6.31	Tyr78 (H-bond), Leu70, Lys80, Ile87, Tyr100, Pro106, Ala110 (alkylic interactions), Arg71, Gly79, Gly82, Ser83, Ser84, Gly85, Val86, Gly107, Asn111, Lys115 (van der Waals interactions)
**3a**	−6.08	Tyr78, Tyr100, Pro106, Gly107 (H-bonds), Glu114 (π-interaction), Ile87, Ala110 (alkylic interactions), Leu70, Arg71, Val86, Ala109, Asn111, Lys115 (van der waals interactions)
**3b**	−6.37	Tyr78 (H-bond), Ala110 (π-interaction), Leu70, Arg71, Tyr78, Ile87 (alkylic interactions), Gly79, Gly85, Val86, Pro106, Gly107, Asn111 (van der Waals interactions)
**3c**	−5.80	Gly79, Val86, Tyr100 (H-bonds), Ala110 (π-interaction), Leu70, Arg71, Pro106 (alkylic interactions), Tyr78, Gly85, Ile87, Gly107, Asn111, Glu114 (van der Waals interactions)
**3d**	−5.70	Gly79, Val86 (H-bonds), Tyr78, Ala110 (π-interactions), Leu70, Arg71, Pro106 (alkylic interactions), Gly85, Ile87, Tyr100, Gly107, Asn111 (van der Waals interactions)
**3e**	−6.14	Tyr78, Ala110 (π-interactions), Leu70, Arg71, Ile87, Pro106 (alkylic interactions), Tyr100, Gly107, Asn111, Glu114 (van der Waals interactions)
**3f**	−6.11	Tyr78, Gly107 (H-bonds), Leu70, Arg71, Pro106, Ala110 (π-interactions), Ile87, Tyr100, Asn111, Glu114 (van der Waals interactions)

**Table 4:** Interaction details of the molecules against *A. castellanii* profilin II.

Compounds	Binding Affinity (kcal/mol)	Amino Acids Residue *A. castellanii* Profilin II
**1a**	−3.37	Tyr78, Pro106 (π-interactions), İle70, Ala110 (alkylic interactions), Arg71, Gly79, Lys80, Gly85, Val86, Tyr100, Gly107, Glu114 (van der Waals interactions)
**1b**	−3.67	Tyr100 (H-bond), Tyr78 (π-interaction), Ile70, Pro106 (alkylic interactions), Arg71, Gly79, Lys80, Gly85, Val86, Ile87, Gly107, Ala110 (van der Waals interactions)
**1c**	−3.46	Tyr78 (H-bond), Glu114 (π-interaction), Ile70, Pro106, Ala110 (alkylic interactions), Arg71, Arg78, Gly79, Lys80, Ile87, Tyr100, Gly107, Asn111, Lys115 (van der Waals interactions)
**1d**	−3.05	Ile87 (π-interaction), Ile70, Arg71 (alkylic interactions), Gly107, Glu114 (van der Waal interactions), Tyr78, Tyr100, Pro106, Ala109, Ala110 (Unfavorable Bumb)
**1e**	−3.08	Tyr78 (H-bond), Ile70, Ala110 (π-interactions), Pro106 (alkylic interaction), Arg71, Lys80, Tyr100, Gly107, Asn111, Glu114, Lys115 (van der Waal interactions)
**1f**	−3.31	Tyr100 (H-bond), Ile70 (alkylic interaction), Val77, Lys80, Thr88, Gly107 (van der Waals interactions), Tyr78, Gly79, Val86, Ile87, Pro106, Ala110 (Unfavorable Bump)
**3a**	−4.38	Tyr78 (H-bond), Glu114 (π-interaction), Ile70, Lys80, Pro106, Ala110 (alkylic interactions), Arg71, Tyr100, Gly107, Asn111, Lys115 (van der Waals interactions)
**3b**	−4.41	Tyr78, Pro106, Ala110 (alkylic interactions), Ile70, Arg71, Lys80, Ser83, Ala84, Tyr100, Glu102, Ile104, Gln105, Gly107 (van der Waals interactions)
**3c**	−5.22	Tyr78, Glu114 (π-interactions), Ile70, Pro106, Ala110 (alkylic interactions), Arg71, Arg75, Ile87, Tyr100, Gly107, Lys115 (van der Waals interactions)
**3d**	−4.04	Tyr100, Pro106 (H-bonds), Arg71 (π-interaction), Ile70, Lys80 (alkylic interactions), Tyr78, Gly79, Gly85, Ile87, Gly107, Ala110 (van der Waals interactions)
**3e**	−4.11	Ile70 (π-interaction), Lys80, Pro106 (alkylic interaction), Arg71, Tyr78, Tyr100, Ala110 (van der Waals interactions)
**3f**	−3.52	Ala110, Glu114 (π-interactions), Ile70, Lys80, Pro106 (alkylic interactions), Arg71, Tyr78, Tyr100, Gly107, Asn111, Lys115 (van der Waals interactions)

**Table 5:** Calculated logP [63] for silver(I) complexes **1a**–**f**.

**Silver Complex**	**Calculated logP**	**Silver Complex**	**Calculated logP**
**1a**	4.60	**1d**	4.52
**1b**	5.01	**1e**	5.14
**1c**	4.52	**1f**	6.69

**Table 6:** Selected crystallographic data for complex **1b**.

CCDC depository	2,482,191	Chemical formula	C_48_H_44_Ag_2_Br_2_N_4_
Molar mass (g·mol^−1^)	1052.43	Temperature (K)	120(2)
Crystal system	Triclinic	Space group	$P\bar{1}$
*a* (Å)	8.7251(4)	*α* (°)	69.405(2)
*b* (Å)	10.7811(6)	*β* (°)	78.683(2)
*c* (Å)	11.7674(6)	*γ* (°)	86.031(3)
Volume (Å^3^)	1016.03(9)	*Z*	1
*ρ*_calc._ (g·cm^−3^)	1.720	*μ* (mm^−1^)	2.970
*F*000	524	Crystal size (mm)	0.140 × 0.130 × 0.120
*θ* range for data collection (°)	1.881 ≤ *θ* ≤ 27.877	*Rint*	0.0538
Reflections collected	34781	Goodness-of-fit on *F*^2^	1.018
*R*_1_, *wR*_2_ (all data)	*R*_1_ = 0.0473 *wR*_2_ = 0.0894	*R*_1_, *wR*_2_ [*I* > 2σ(*I*)]	*R*_1_ = 0.0359 *wR*_2_ = 0.0819

## Data Availability

The data presented in this study are available upon request from the corresponding authors.

## Supplementary Materials

The following supporting information can be downloaded at https://www.mdpi.com/article/10.3390/ijms26199393/s1.

## References

[1] Marciano-Cabral F., Cabral G. (2003). *Acanthamoeba* spp. as agents of disease in humans. Clin. Microbiol. Rev..

[2] Siddiqui R., Khan N.A. (2012). Biology and pathogenesis of *Acanthamoeba*. Parasit. Vectors.

[3] Varacalli G., Di Zazzo A., Mori T., Dohlman T.H., Spelta S., Coassin M., Bonini S. (2021). Challenges in *Acanthamoeba* Keratitis: A review. J. Clin. Med..

[4] Bahrami S., Darvishi M., Zarei M., Sabaeian M., Henriquez F.L. (2023). Sublethal exposure to plasma-activated water influences the morphological characteristics, phagocytic ability, and virulence of *Acanthamoeba castellanii*. Acta Parasitol..

[5] Iovieno A., Miller D., Ledee D.R., Alfonso E.C. (2014). Cysticidal activity of antifungals against different genotypes of *Acanthamoeba*. Antimicrob. Agents Chemother..

[6] Khan N.A. (2006). *Acanthamoeba*: Biology and increasing importance in human health. FEMS Microbiol. Rev..

[7] Khan N.A., Paget T.A. (2002). Molecular tools for speciation and epidemiological studies of *Acanthamoeba*. Curr. Microbiol..

[8] Illingworth C.D., Cook S.D. (1998). *Acanthamoeba* Keratitis. Surv. Ophthalmol..

[9] Padhi T.R., Das S., Sharma S., Rath S., Rath S., Tripathy D., Panda K.G., Basu S., Besirli C.G. (2017). Ocular parasitoses: A comprehensive review. Surv. Ophthalmol..

[10] Jones D.B., Visvesvara G.S., Robinson N.M. (1975). *Acanthamoeba polyphaga* keratitis and *Acanthamoeba* uveitis associated with fatal meningoencephalitis. Trans. Ophthalmol. Soc. UK.

[11] Martinez A.J., Visvesvara G.S. (1997). Free-living, *Amphizoic* and *Opportunistic amebas*. Brain Pathol..

[12] Fanselow N., Sirajuddin N., Yin X.-T., Huang A.J.W., Stuart P.M. (2021). *Acanthamoeba* keratitis, pathology, diagnosis and treatment. Pathogens.

[13] Zhang Y., Xu X., Wei Z., Cao K., Zhang Z., Liang Q. (2023). The global epidemiology and clinical diagnosis of *Acanthamoeba* keratitis. J. Infect. Public Health.

[14] Petrillo F., Tortori A., Vallino V., Galdiero M., Fea A.M., De Sanctis U., Reibaldi M. (2024). Understanding *Acanthamoeba* keratitis: An in-depth review of a sight-threatening eye infection. Microorganisms.

[15] Wang Y., Jiang L., Zhao Y., Ju X., Wang L., Jin L., Fine R.D., Li M. (2023). Biological characteristics and pathogenicity of *Acanthamoeba*. Front. Microbiol..

[16] Cabrera-Aguas M., Khoo P., Watson S.L. (2022). Infectious keratitis: A review. Clin. Experiment Ophthalmol..

[17] Gokhale N.S. (2008). Medical management approach to infectious keratitis. Indian J. Ophthalmol..

[18] Shareef O., Shareef S., Saeed H.N. (2023). New frontiers in *Acanthamoeba* keratitis diagnosis and management. Biology.

[19] Durand M.L., Barshak M.B., Chodosh J. (2021). Infectious keratitis in 2021. JAMA.

[20] Goh J.W., Harrison R., Hau S., Alexander C.L., Tole D.M., Avadhanam V.S. (2018). Comparison of *in vivo* confocal microscopy, PCR and culture of corneal scrapes in the diagnosis of *Acanthamoeba* keratitis. Cornea.

[21] Hopkinson M.N., Richter C., Schedler M., Glorius F. (2014). An overview of *N*-heterocyclic carbenes. Nature.

[22] Öfele K. (1968). 1, 3-Dimethyl-4-imidazolinyliden-(2)-pentacarbonylchrom ein neuer Übergangsmetall-carben-komplex. J. Organomet. Chem..

[23] Wanzlick H.W., Schönherr H.J. (1968). Direct synthesis of a mercury salt-carbene complex. Angew. Chem. Int. Ed..

[24] Arduengo A.J., Harlow R.L., Kline M. (1991). A stable crystalline carbene. J. Am. Chem. Soc..

[25] Kühl O. (2007). The chemistry of functionalised *N*-heterocyclic carbenes. Chem. Soc. Rev..

[26] Normand A.T., Cavell K.J. (2008). Donor-functionalised *N*-heterocyclic carbene complexes of group 9 and 10 metals in catalysis: Trends and directions. Eur. J. Inorg. Chem..

[27] Kascatan-Nebioglu A., Panzner M.J., Tessier C.A., Cannon C.L., Youngs W.J. (2007). *N*-Heterocyclic carbene-silver complexes: A new class of antibiotics. Coord. Chem. Rev..

[28] Gasser G., Ott I., Metzler-Nolte N. (2011). Organometallic anticancer compounds. J. Med. Chem..

[29] Budagumpi S., Haque R.A., Salman A.W. (2012). Stereochemical and structural characteristics of single-and double-site Pd(II)-*N*-heterocyclic carbene complexes: Promising catalysts in organic syntheses ranging from CC coupling to olefin polymerizations. Coord. Chem. Rev..

[30] Ceramella J., Catalano A., Mariconda A., D’Amato A., Aquila S., Saturnino C., Rosano C., Sinicropi M.S., Longo P. (2025). Silver *N*-heterocyclic carbene (NHC) complexes as antimicrobial and/or anticancer agents. Pharmaceuticals.

[31] Raju S.K., Karunakaran A., Kumar S., Sekar P., Murugesan M., Karthikeyan M. (2022). Silver complexes as anticancer agents: A perspective review. Ger. J. Pharm. Biomater..

[32] Ferreira L.G., Dos Santos R.N., Oliva G., Andricopulo A.D. (2015). Molecular docking and structure-based drug design strategies. Molecules.

[33] Singh A., Singh K., Sharma A., Kaur K., Chadha R., Bedi P.M.S. (2023). Recent advances in antifungal drug development targeting lanosterol 14α-demethylase (CYP51): A comprehensive review with structural and molecular insights. Chem. Biol. Drug Des..

[34] Bubb M.R., Baines I.C., Korn E.D. (1998). Localization of actobindin, profilin I, profilin II, and phosphatidylinositol-4, 5-bisphosphate (PIP2) in *Acanthamoeba castellanii*. Cell Motil. Cytoskelet..

[35] Akın-Polat Z., Şahin N., Hkiri S., Ly B.M.T., Özdemir İ., Sémeril D. (2025). *In vitro* evaluation of silver-NHC complexes against a clinical isolate of *Acanthamoeba castellanii*: Time-and dose-dependent effects. Inorganics.

[36] Dos Santos D.L., Chaúque B.J.M., Berté F.K., de Miranda Ribeiro L., Matiazo F.F., Rott M.B., Schrekker H.S., Sekine L. (2025). Imidazolium salt as potent amoebicide for rapid inactivation of *Acanthamoeba* spp. trophozoites and cysts. Exp. Parasitol..

[37] Tutar U., Çelik C., Üstün E., Özdemir N., ¸Sahin N., Sémeril D., Gürbüz N., Özdemir İ. (2023). Benzimidazol-2-ylidene silver complexes: Synthesis, characterization, antimicrobial and antibiofilm activities, molecular docking and theoretical investigations. Inorganics.

[38] Garrison J.C., Youngs W.J. (2005). Ag(I) *N*-heterocyclic carbene complexes:  synthesis, structure, and application. Chem. Rev..

[39] Tulloch A.A.D., Danopoulos A.A., Winston S., Kleinhenz S., Eastham G. (2000). *N*-Functionalised heterocyclic carbene complexes of silver. J. Chem. Soc. Dalton Trans..

[40] Lin I.J.B., Vasam C.S. (2004). Silver(I) *N*-heterocyclic carbenes. Comments Inorg. Chem..

[41] Chen W., Liu F. (2003). Synthesis and characterization of oligomeric and polymeric silver-imidazol-2-ylidene iodide complexes. J. Organomet. Chem..

[42] Guarra F., Busto N., Guerri A., Marchetti L., Marzo T., García B., Biver T., Gabbiani C. (2020). Cytotoxic Ag(I) and Au(I) NHC-carbenes bind DNA and show TrxR inhibition. J. Inorg. Biochem..

[43] de Lacerda A.G., Lira M. (2020). *Acanthamoeba* keratitis: A review of biology, pathophysiology and epidemiology. Ophthalmic Physiol. Opt..

[44] Ilyas M., Stapleton F., Willcox M.D.P., Henriquez F., Peguda H.K., Rayamajhee B., Zahid T., Petsoglou C., Carnt N.A. (2024). Epidemiology of and genetic factors associated with *Acanthamoeba* keratitis. Pathogens.

[45] Reyes-Batlle M., Sifaoui I., Rodríguez-Expósito R.L., Piñero J.E., Lorenzo-Morales J. (2022). New insights in *Acanthamoeba*. Pathogens.

[46] Lee S.M., Lee J.E., Lee D.I., Yu H.S. (2018). Adhesion of *Acanthamoeba* on cosmetic contact lenses. J. Korean Med. Sci..

[47] Hendiger E.B., Padzik M., Sifaoui I., Reyes-Batlle M., López-Arencibia A., Rizo-Liendo A., Bethencourt-Estrella C.J., Nicolás-Hernández D.S., Chiboub O., Rodríguez-Expósito R.L. (2020). Silver nanoparticles as a novel potential preventive agent against *Acanthamoeba* keratitis. Pathogens.

[48] Debeljak N., Fink M., Rozman D. (2003). Many facets of mammalian lanosterol 14α-demethylase from the evolutionarily conserved cytochrome P450 family CYP51. Arch. Biochem. Biophys..

[49] Hargrove T.Y., Lamb D.C., Wawrzak Z., Hull M., Kelly S.L., Guengerich F.P., Lepesheva G.I. (2024). Identification of potent and selective inhibitors of *Acanthamoeba*: Structural insights into sterol 14*α*-demethylase as a key drug target. J. Med. Chem..

[50] Abdel-Hakeem S.S., Hassan F.A.M., Hifney A.F., Salem S.H. (2024). Combating the causative agent of amoebic keratitis, *Acanthamoeba castellanii*, using Padina pavonica alcoholic extract: Toxicokinetic and molecular docking approaches. Sci. Rep..

[51] Saeed B.Q., Hamdy R., Akbar N., Sajeevan S.E., Khan N.A., Soliman S.S. (2024). Azole-based compounds as potential anti-*Acanthamoeba* agents. RSC Med. Chem..

[52] Kurian T., Sebastian R. (2024). Molecular docking study of heterocyclic compounds for antifungal activity against Granulomatous Amoebic Encephalitis. J. Pharm. Res..

[53] Aksu A., Çelik M.S., Polat Z.A., Yenidünya A.F., Çetinkaya S., Tüzün B. (2024). Experimental and theoretical evidence on the amoebicidal activity of synthesized tRNA-palmitic acid esters. Iran. J. Chem. Chem. Eng..

[54] Witke W. (2004). The role of profilin complexes in cell motility and other cellular processes. Trends Cell Biol..

[55] Bubb M.R., Korn E.D. (1991). [11] Purification of actobindin from *Acanthamoeba castellanii*. Methods Enzymol..

[56] Khairul W.M., Hashim F., Rahamathullah R., Mohammed M., Razali S.A., Johari S.A.T.T., Azizan S. (2024). Exploring ethynyl-based chalcones as green semiconductor materials for optical limiting interests. Spectrochim. Acta A Mol. Biomol. Spectrosc..

[57] Zamli K.M., Hashim F., Razali S.A., Yusoff H.M., Mohamad H., Abdullah F., Asari A. (2023). Synthesis, anti-amoebic activity and molecular docking simulation of eugenol derivatives against *Acanthamoeba* sp. Saudi Pharm. J..

[58] Hermosaningtyas A.A., Budzianowska A., Kruszka D., Derda M., Długaszewska J., Kikowska M. (2025). Can *in vitro* cell cultures of *Eryngium planum*, *Lychnis flos-cuculi*, and *Kickxia elatine* be an alternative source of plant biomass with biological antimicrobial and anti-*Acanthamoeba* activities?. Appl. Sci..

[59] Meng G., Kakalis L., Nolan S.P., Szostak M. (2019). A simple ^1^H NMR method for determining the σ-donor properties of *N*-heterocyclic carbenes. Tetrahedron Lett..

[60] Asekunowo P.O., Haque R.A., Razali M.R. (2017). A comparative insight into the bioactivity of mono- and binuclear silver(I)-*N*-heterocyclic carbene complexes: Synthesis, lipophilicity and substituent effect. Rev. Inorg. Chem..

[61] Saczewski J., Popenda Ł., Fedorowicz J. (2024). *In silico* SwissADME analysis of antibacterial NHC-Silver acetates and halides complexes. Appl. Sci..

[62] Ronga L., Varcamonti M., Tesauro D. (2023). Structure-activity relationships in NHC-silver complexes as antimicrobial agents. Molecules.

[63] Admetlab. https://admetlab3.scbdd.com.

[64] Tan K.L., Vasudevan A., Bergman R.G., Ellman J.A., Souers A.J. (2003). Microwave-assisted C-H bond activation: A rapid entry into functionalized heterocycles. Org. Lett..

[65] Sheldrick G.M. (2015). SHELXT-Integrated space-group and crystal-structure determination. Acta Crystallogr. Sect. A Found. Adv..

[66] Sheldrick G.M. (2015). Crystal structure refinement with SHELXL. Acta Crystallogr. Sect. C Struct. Chem..

[67] Walochnik J., Obwaller A., Aspöck H. (2000). Correlations between morphological, molecular biological, and physiological characteristics in clinical and nonclinical isolates of *Acanthamoeba* spp. *Appl*. Environ. Microbiol..

[68] Neese F. (2012). The ORCA program system. WIREs Comput. Mol. Sci..

[69] Neese F. (2018). Software update: The ORCA program system, version 4.0. WIREs Comput. Mol. Sci..

[70] Cosconati S., Forli S., Perryman A.L., Harris R., Goodsell D.S., Olson A.J. (2010). Virtual screening with AutoDock: Theory and practice. Expert Opin. Drug Discov..

[71] Sharma V., Shing B., Hernandez-Alvarez L., Debnath A., Podust L.M. (2020). Domain-swap dimerization of *Acanthamoeba castellanii* CYP51 and a unique mechanism of inactivation by isavuconazole. Mol. Pharmacol..

[72] Liu S., Fedorov A.A., Pollard T.D., Lattman E.E., Almo S.C., Magnus K.A. (1998). Crystal packing induces a conformational change in profilin-I from *Acanthamoeba castellanii*. J. Struct. Biol..

[73] Fedorov A.A., Magnus K.A., Graupe M.H., Lattman E.E., Pollard T.D., Almo S.C. (1994). X-ray structures of isoforms of the actin-binding protein profilin that differ in their affinity for phosphatidylinositol phosphates. Proc. Natl. Acad. Sci. USA.

[74] Morris G.M., Huey R., Lindstrom W., Sanner M.F., Belew R.K., Goodsell D.S., Olson A.J. (2009). AutoDock4 and AutoDockTools4: Automated docking with selective receptor flexibility. J. Comput. Chem..

[75] Hkiri S., Coşkun K.A., Üstün E., Samarat A., Tutar Y., Şahin N., Sémeril D. (2022). Silver(I) complexes based on oxadiazole-functionalized *α*-aminophosphonate: Synthesis, structural study, and biological activities. Molecules.

