# Supplementary material for: Silver(I)-NHC Complexes as Dual-Action Agents Against Pathogenic Acanthamoeba Trophozoites: Anti-Amoebic and Anti-Adhesion Activities
Source: Int J Mol Sci. 2025 Sep 25;26(19):9393. doi: 10.3390/ijms26199393 (PMC12524456; doi:10.3390/ijms26199393)
Supplement: Supplementary file 1 [file ijms-26-09393-s001.zip › ijms-3873388-supplementary.pdf]

# Silver(I)-NHC complexes as dual-action agents against pathogenic Acanthamoeba trophozoites: Anti-amoebic and anti-adhesion activities

Shaima Hkiri, Neslihan Şahin, Zübeyda Akın-Polat, Elvan Üstün, Bui Minh Thu Ly, İsmail Özdemir and David Sémeril

## Contents

Characterizing data of benzimidazolium salts

1-benzyl-3-cinnamyl-benzimidazolium bromide (**3a**) p 2

1-(4-methylbenzyl)-3-cinnamyl-benzimidazolium bromide (**3b**) p 5

1-(3-methoxylbenzyl)-3-cinnamyl-benzimidazolium bromide (**3c**) p 9

1-(3,5-dimethoxy-benzyl)-3-cinnamyl-benzimidazolium bromide (**3d**) p 13

1-(naphthalen-1-ylmethyl)-3-cinnamyl-benzimidazolium bromide (**3e**) p 17

1-(pyren-1-ylmethyl)-3-cinnamyl-benzimidazolium bromide (**3f**) p 21

Characterizing data of silver(I) complexes

bromo(1-benzyl-3-cinnamyl-benzimidazol-2-ylidene)silver (I) (**1a**) p 25

bromo[1-(4-methylbenzyl)-3-cinnamyl-benzimidazol-2-yliden]silver(I) (**1b**) p 29

bromo[1-(3-methoxylbenzyl)-3-cinnamyl-benzimidazol-2-yliden]silver(I) (**1c**) p 33

bromo[1-(3,5-dimethoxy-benzyl)-3-cinnamyl-benzimidazol-2-ylidene]silver(I) (**1d**) p 37

bromo[1-(naphthalen-1-ylmethyl)-3-cinnamyl-benzimidazol-2 ylidene]silver(I) (**1e**) p 41

bromo[1-(pyren-1-ylmethyl)-3-cinnamyl-benzimidazol-2-yliden]silver(I) (**1f**) p 45

Interaction detail and interaction residue of tested compounds against

*Acanthamoeba castellanii* CYP51 p 49

*Acanthamoeba castellanii* profilin IA p 53

*Acanthamoeba castellanii* profilin IB p 57

*Acanthamoeba castellanii* profilin II p 61

**1-Benzyl-3-cinnamyl-benzimidazolium bromide (3a)**

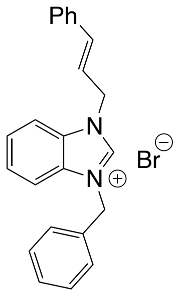


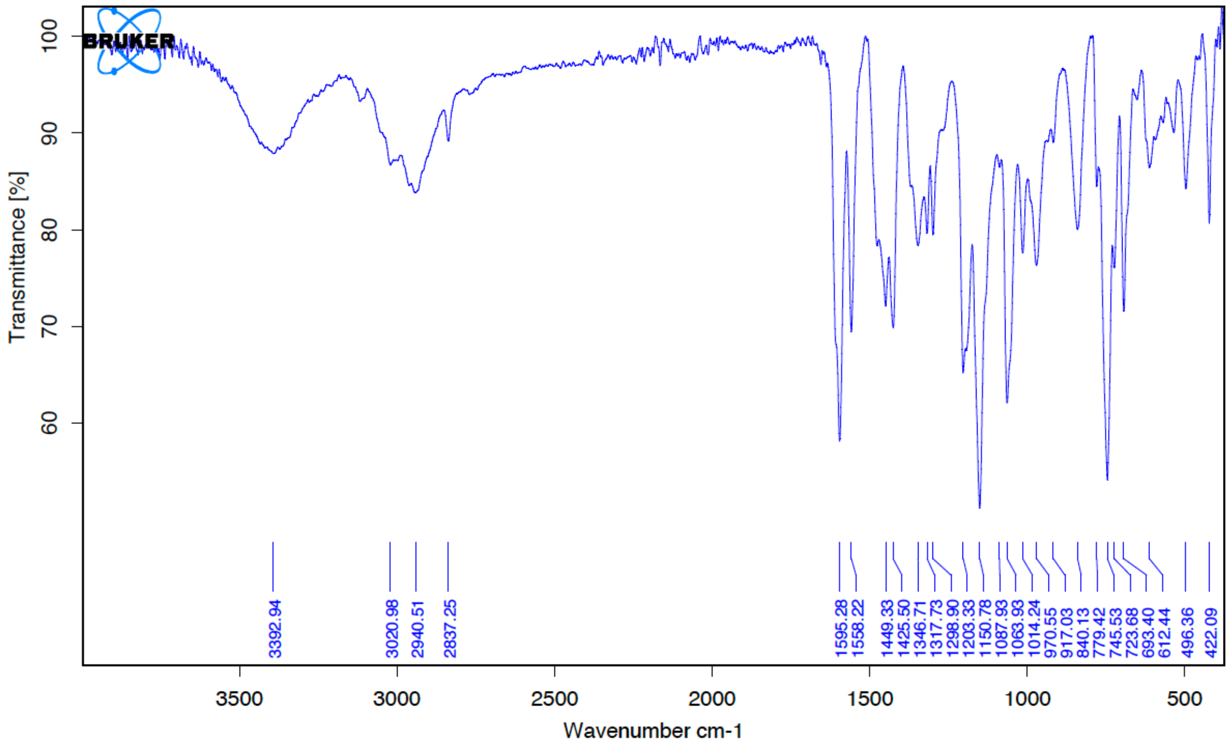


**Figure S1**. FT-IR spectrum

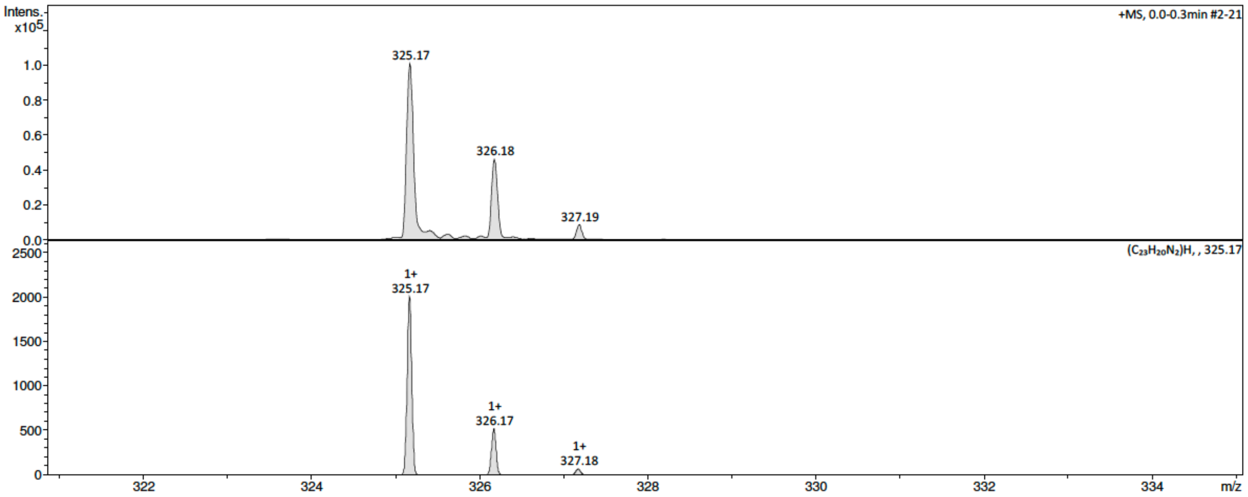


**Figure S2**. Mass spectrum (ESI-TOF): exp. spectrum (top); calc. spectrum (bottom) for $C_{23}H_{21}N_2$ ($[M - Br]^+$)

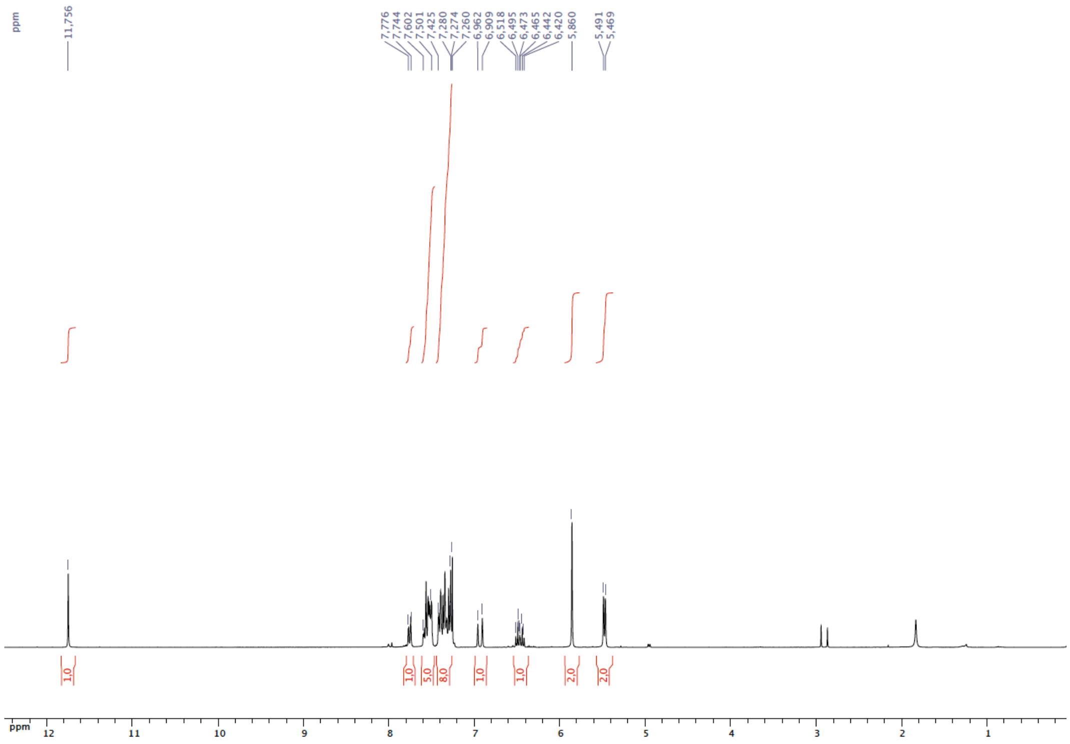


**Figure S3**. $^{1}$H NMR spectrum ($CDCl_3$)

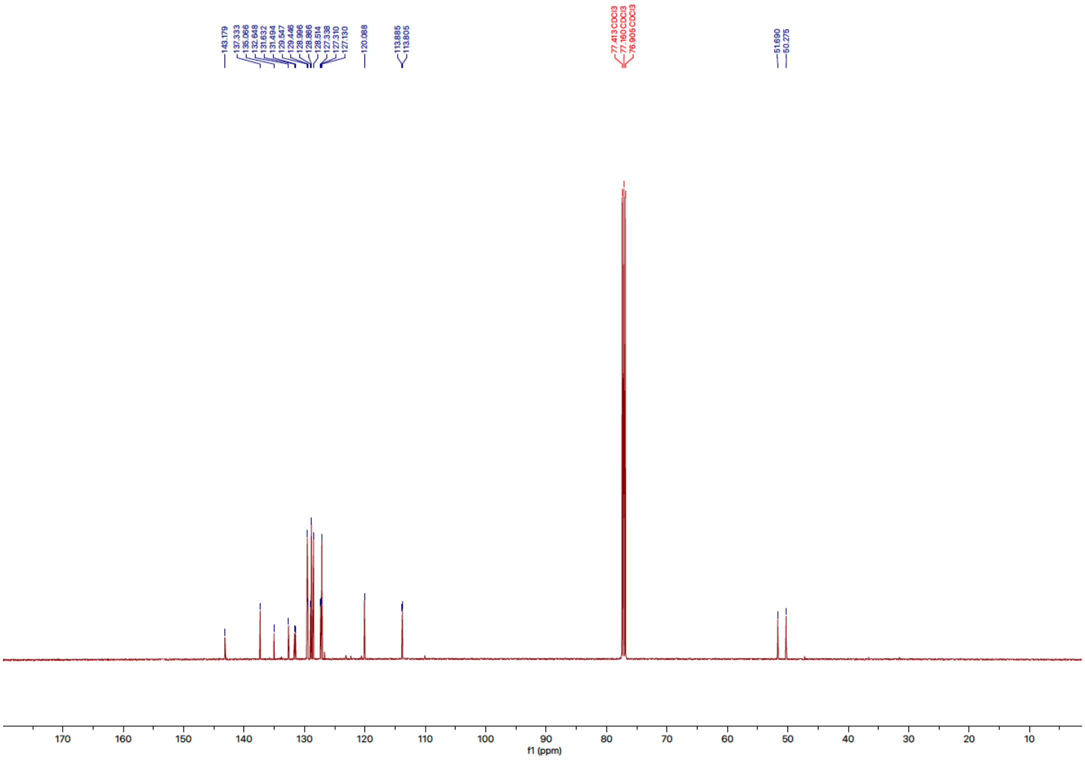


**Figure S4**. $^{13}C\{^{1}H\}$ NMR spectrum ($CDCl_3$)

**Table S1.** Optimized Cartesian Coordinates of **3a**

| **Atom** | **Cartesian Coordinates** | | |
|---|---|---|---|
| | **x** | **y** | **z** |
| N | -0,94092 | 1,986133 | 0,344322 |
| N | 0,894162 | 0,909268 | -0,1943 |
| C | -0,0613 | 2,154637 | 1,42852 |
| C | 1,12942 | 1,465209 | 1,0803 |
| C | -0,35225 | 1,229399 | -0,60814 |
| C | -2,84003 | 2,260535 | -1,17464 |
| C | -0,19017 | 2,832836 | 2,651791 |
| C | 2,237933 | 1,436 | 1,942868 |
| C | -2,29143 | 2,549565 | 0,206494 |
| C | 2,111451 | 2,12479 | 3,158573 |
| C | 0,921139 | 2,807481 | 3,507751 |
| C | -2,16224 | 2,754512 | -2,32369 |
| C | -4,01778 | 1,509034 | -1,34228 |
| C | -3,82188 | 1,722368 | -3,76718 |
| C | 1,834744 | 0,152375 | -1,02049 |
| C | -4,50824 | 1,243634 | -2,63111 |
| C | -2,6479 | 2,474949 | -3,6121 |
| H | 2,96015 | 2,133387 | 3,858934 |
| H | 0,866823 | 3,331996 | 4,473012 |
| C | 2,205084 | -1,21188 | -0,51592 |
| C | 1,628088 | -1,87326 | 0,525565 |
| C | 1,97582 | -3,20301 | 1,021592 |
| C | 1,259819 | -3,7202 | 2,137653 |
| H | -1,1122 | 3,363258 | 2,928862 |
| H | 3,161114 | 0,900678 | 1,681775 |
| H | -2,22762 | 3,64422 | 0,391853 |
| H | -2,94494 | 2,115865 | 0,990821 |
| H | -1,27266 | 3,392606 | -2,20414 |
| H | -4,55401 | 1,124074 | -0,46132 |
| H | -4,21533 | 1,51365 | -4,77338 |
| H | 2,750201 | 0,768368 | -1,16704 |
| H | 1,360937 | 0,073751 | -2,02746 |
| H | -5,4308 | 0,656692 | -2,75437 |
| H | -2,11853 | 2,865493 | -4,49364 |
| H | 3,009613 | -1,69115 | -1,09925 |
| H | 0,808347 | -1,37304 | 1,070576 |
| C | 2,99948 | -4,00904 | 0,444911 |
| C | 1,55174 | -4,98498 | 2,657258 |
| H | 0,466389 | -3,10786 | 2,594632 |
| H | 3,569761 | -3,64168 | -0,42101 |
| C | 3,288128 | -5,27227 | 0,964175 |
| C | 2,56765 | -5,7665 | 2,072534 |
| H | 4,08014 | -5,8845 | 0,507306 |
| H | 2,799996 | -6,76232 | 2,478673 |
| H | 0,990415 | -5,36858 | 3,522196 |

## 1-(4-Methylbenzyl)-3-cinnamyl-benzimidazolium bromide (3b)

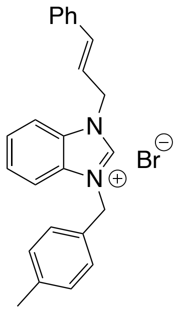


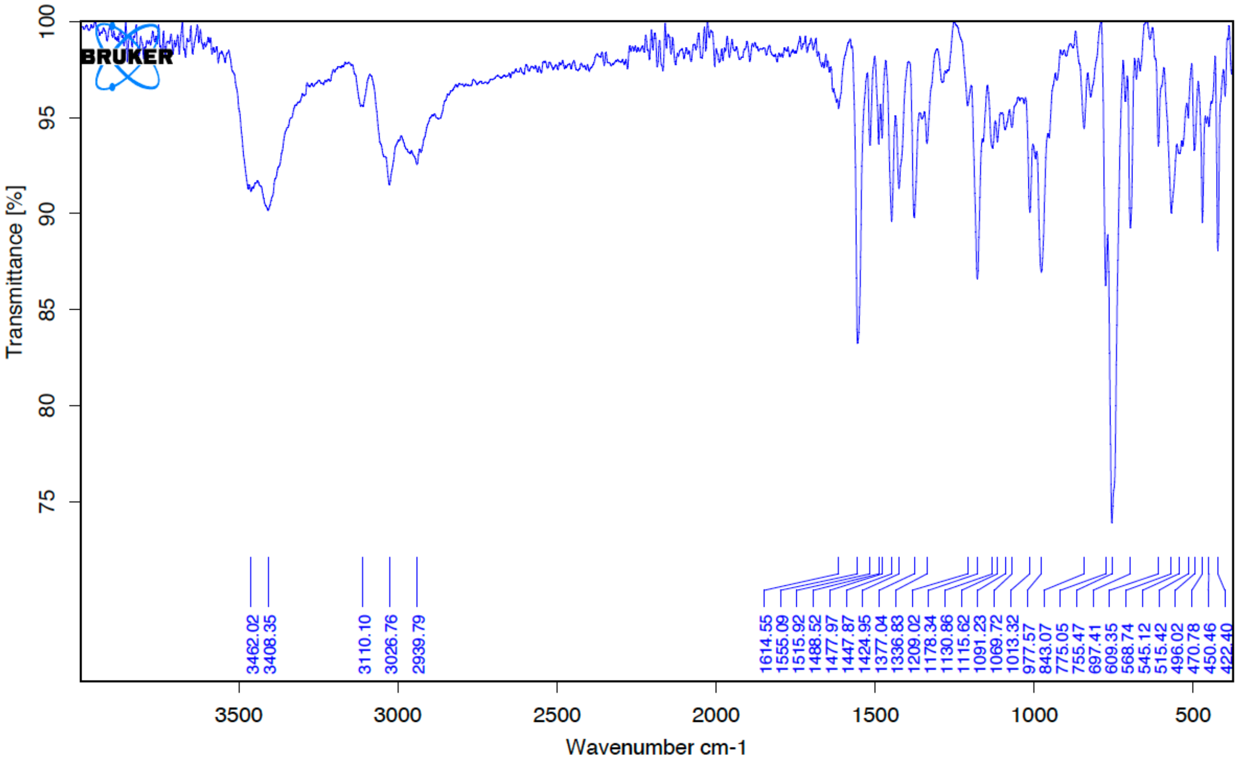


**Figure S5**. FT-IR spectrum

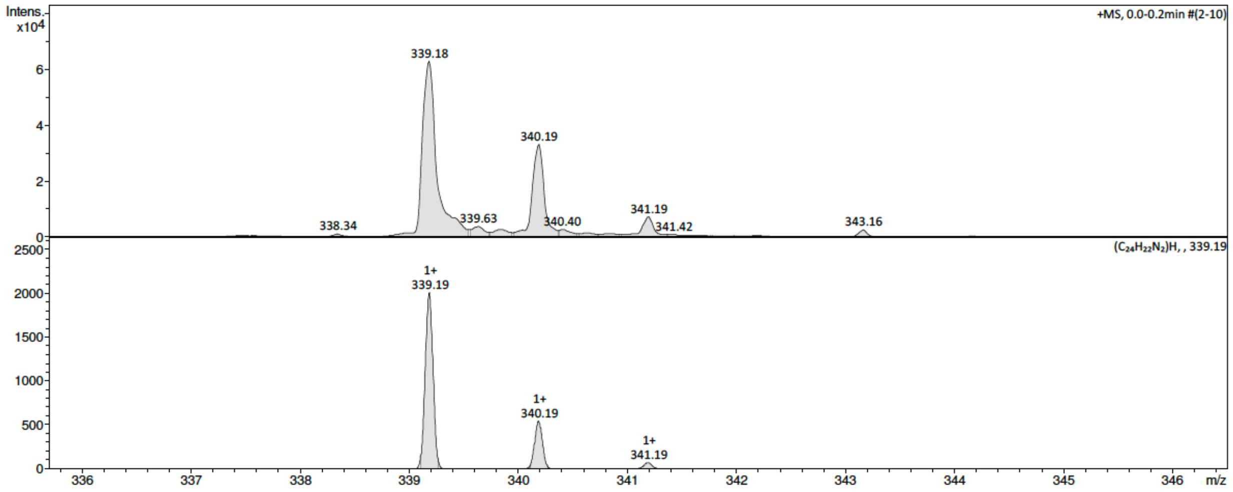


**Figure S6**. Mass spectrum (ESI-TOF): exp. spectrum (top);

calc. spectrum (bottom) for $C_{24}H_{23}N_2$ ($[M - Br]^+$)

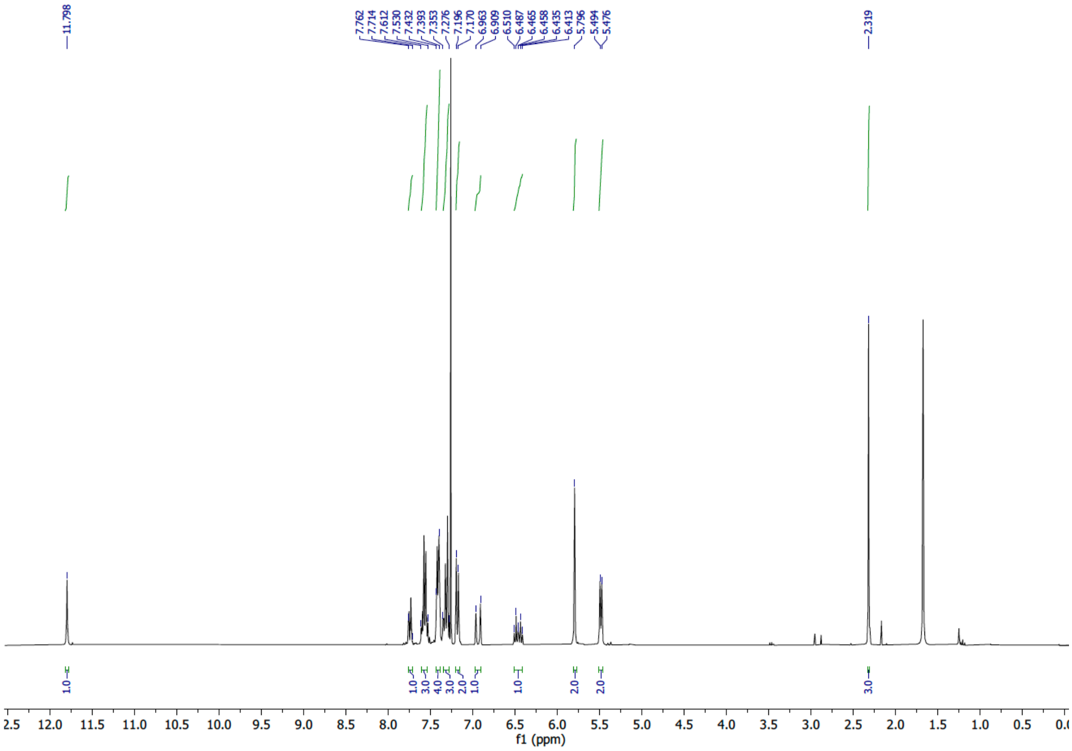


**Figure S7**. $^{1}$H NMR spectrum ($CDCl_3$)

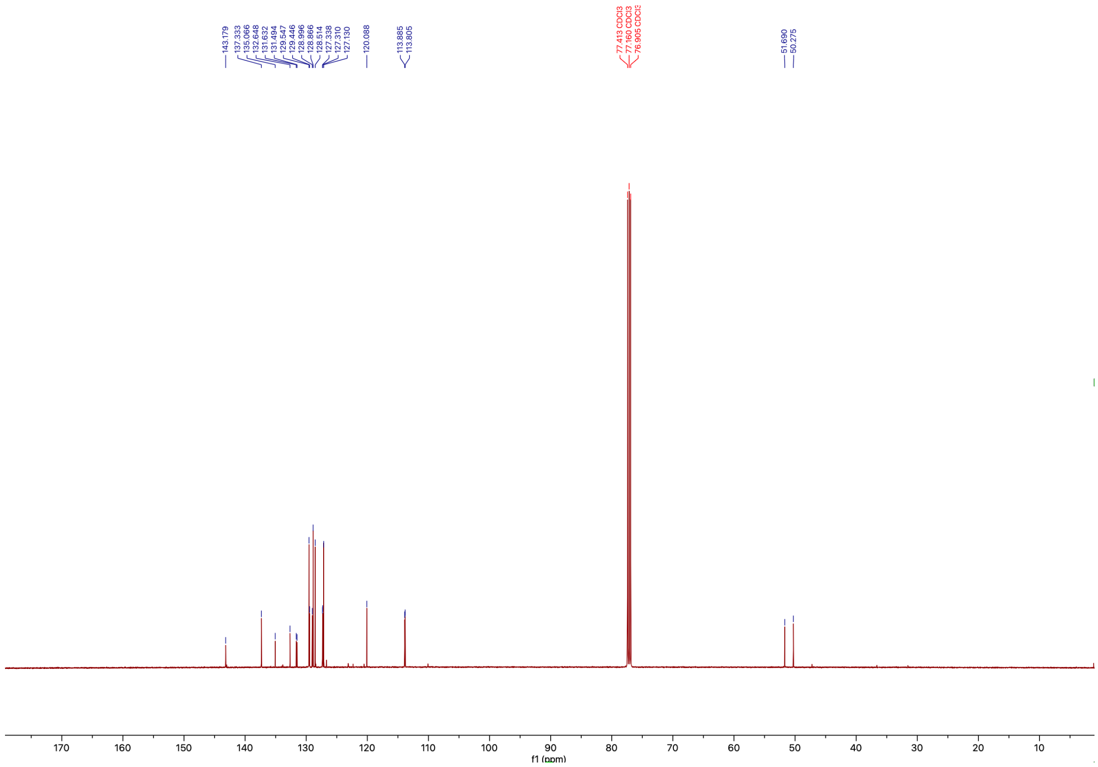


**Figure S8**. $^{13}$C{$^{1}$H} NMR spectrum ($CDCl_3$)

**Table S2.** Optimized Cartesian Coordinates of **3b**

| **Atom** | **Cartesian Coordinates** | | |
|---|---|---|---|
| | **x** | **y** | **z** |
| N | -0,77054 | 1,82958 | 0,510082 |
| N | 1,052398 | 0,750488 | -0,05702 |
| C | 0,129953 | 2,011219 | 1,573666 |
| C | 1,313927 | 1,320207 | 1,205999 |
| C | -0,20389 | 1,062747 | -0,44993 |
| C | -2,65817 | 2,129005 | -1,01088 |
| C | 0,026365 | 2,700409 | 2,793281 |
| C | 2,440689 | 1,301409 | 2,045102 |
| C | -2,12364 | 2,382533 | 0,382643 |
| C | 2,339428 | 2,001189 | 3,256892 |
| C | 1,155712 | 2,684971 | 3,625603 |
| C | -2,01594 | 2,70767 | -2,14322 |
| C | -3,8105 | 1,345906 | -1,21554 |
| C | -3,6627 | 1,697951 | -3,64617 |
| C | 1,977915 | -0,01464 | -0,89239 |
| C | -4,30243 | 1,135447 | -2,51054 |
| C | -2,50734 | 2,485508 | -3,43302 |
| H | 3,202936 | 2,017149 | 3,938728 |
| H | 1,120642 | 3,217251 | 4,587491 |
| C | 2,360724 | -1,37271 | -0,37908 |
| C | 1,806398 | -2,02224 | 0,681107 |
| C | 2,166252 | -3,34633 | 1,186297 |
| C | 1,481345 | -3,84693 | 2,328412 |
| H | -0,89112 | 3,230664 | 3,085533 |
| H | 3,358656 | 0,764374 | 1,76956 |
| H | -2,07404 | 3,472192 | 0,598961 |
| H | -2,78006 | 1,920502 | 1,14841 |
| H | -1,13968 | 3,359372 | -2,00033 |
| H | -4,32837 | 0,895309 | -0,35498 |
| C | -4,20095 | 1,477714 | -5,03432 |
| H | 2,889601 | 0,600702 | -1,06342 |
| H | 1,484875 | -0,10493 | -1,8888 |
| H | -5,20564 | 0,521899 | -2,65332 |
| H | -1,99803 | 2,945966 | -4,29358 |
| H | 3,155632 | -1,85697 | -0,9715 |
| H | 0,997682 | -1,51635 | 1,237317 |
| C | 3,172151 | -4,16169 | 0,59323 |
| C | 1,786496 | -5,10494 | 2,857911 |
| H | 0,701712 | -3,22706 | 2,798999 |
| H | 3,717939 | -3,80766 | -0,29373 |
| C | 3,474014 | -5,41821 | 1,122096 |
| C | 2,784586 | -5,89608 | 2,25666 |
| H | 4,252362 | -6,03774 | 0,651633 |
| H | 3,027118 | -6,8865 | 2,669975 |
| H | 1,249059 | -5,47501 | 3,743622 |

| | | | |
|---|---|---|---|
| H | -3,44337 | 0,984637 | -5,68039 |
| H | -4,44764 | 2,444247 | -5,52251 |
| H | -5,11302 | 0,851978 | -5,03195 |

**1-(3-Methoxylbenzyl)-3-cinnamyl-benzimidazolium bromide (3c)**

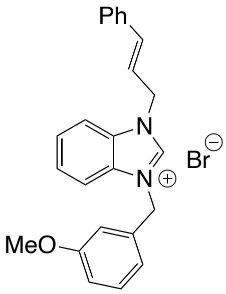


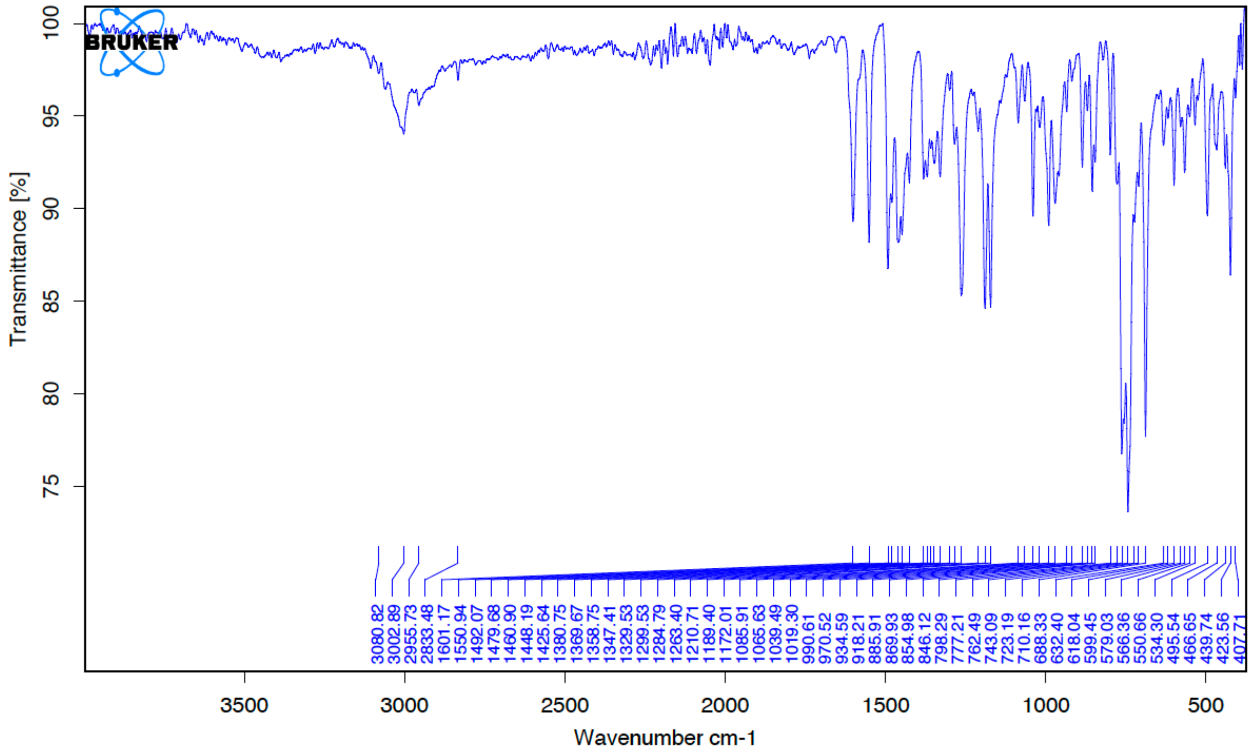


**Figure S9**. FT-IR spectrum

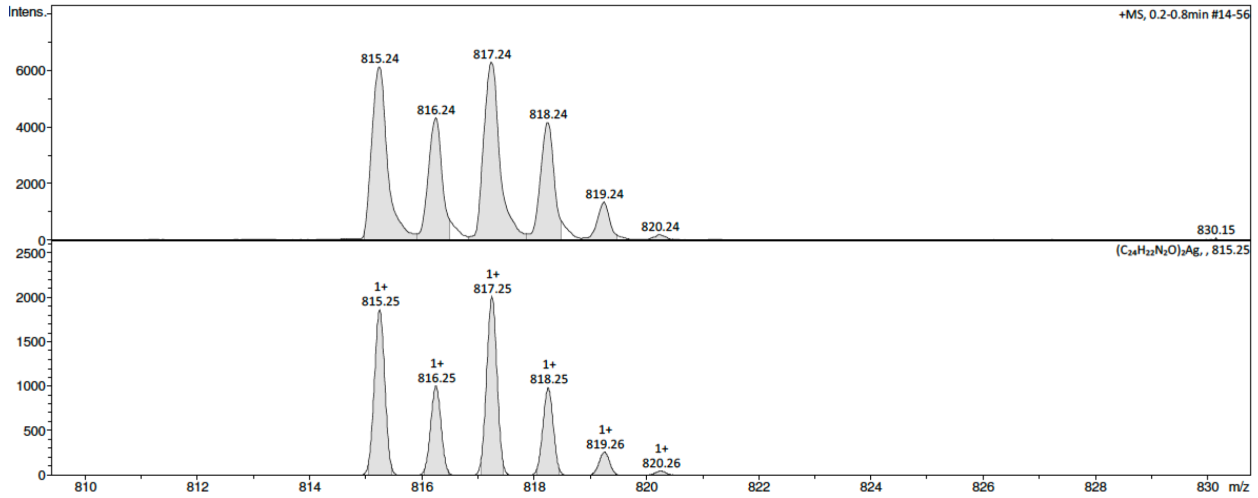


**Figure S10**. Mass spectrum (ESI-TOF): exp. spectrum (top);

calc. spectrum (bottom) for $C_{24}H_{23}N_2O$ ([M - Br]$^+$)

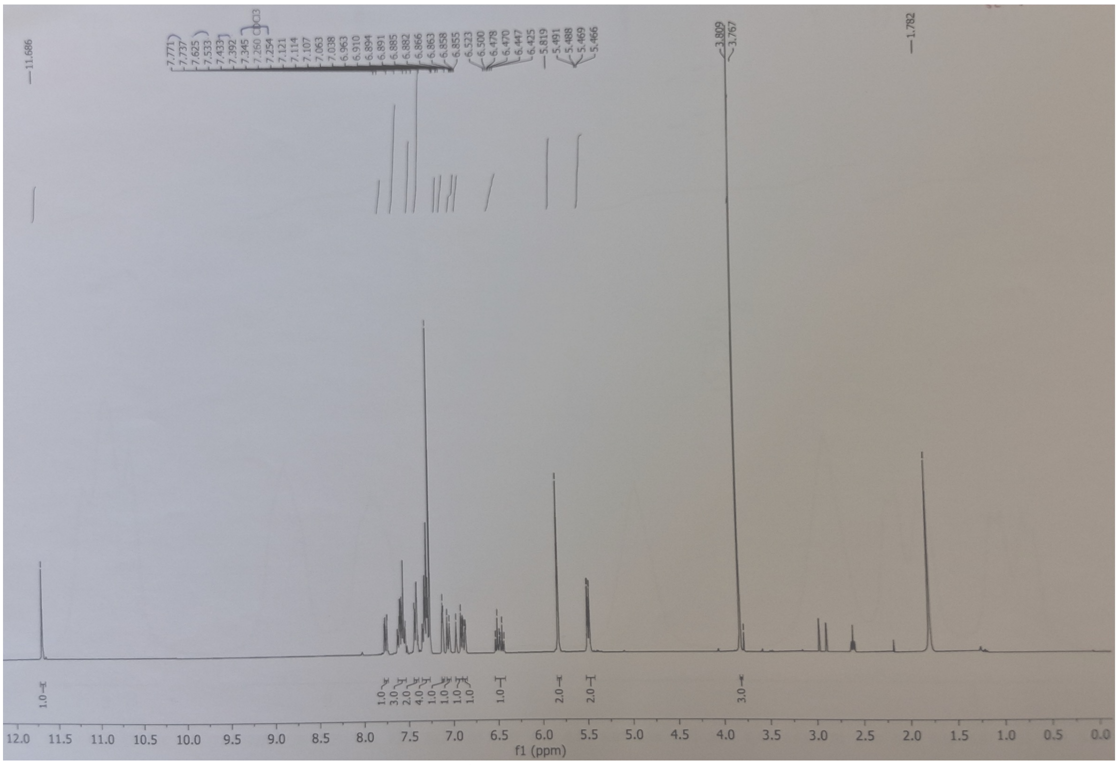

**Figure S11**. $^{1}H$ NMR spectrum ($CDCl_3$)

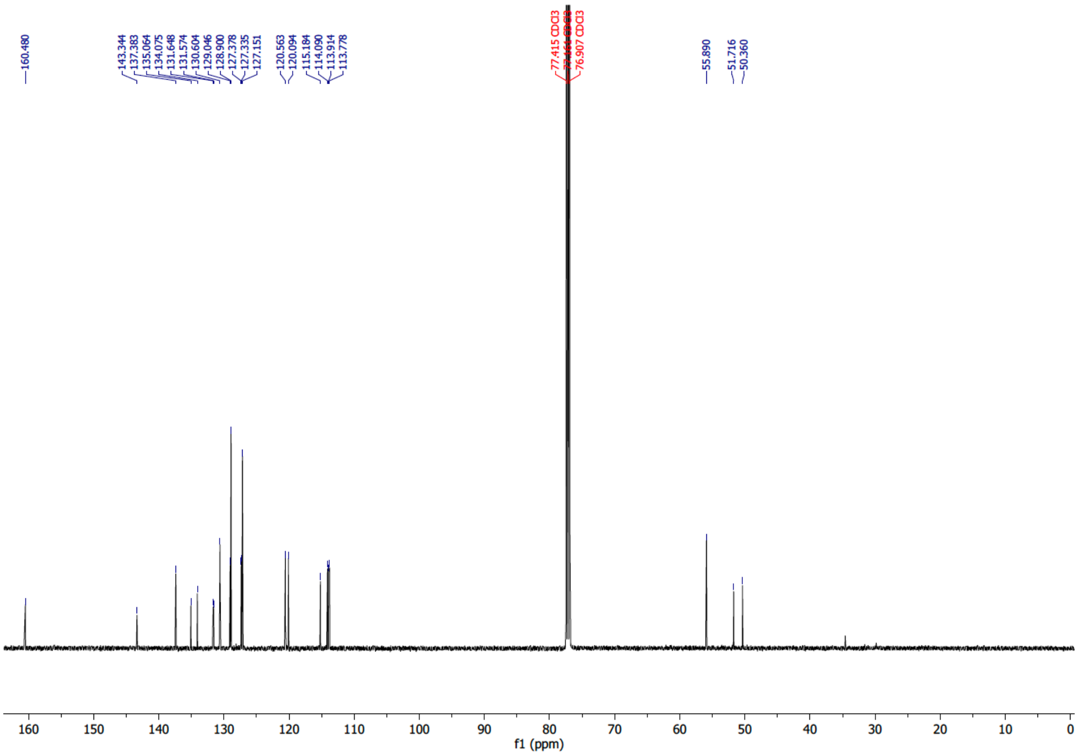

**Figure S12**. $^{13}C\{^{1}H\}$ NMR spectrum ($CDCl_3$)

**Table S3.** Optimized Cartesian Coordinates of **3c**

| **Atom** | **Cartesian Coordinates** | | |
|---|---|---|---|
| | **x** | **y** | **z** |
| N | -0,56972 | 1,575968 | 0,557181 |
| N | 1,181188 | 0,330926 | 0,135292 |
| C | 0,276512 | 1,717894 | 1,670158 |
| C | 1,413379 | 0,915333 | 1,39665 |
| C | -0,00946 | 0,74636 | -0,35564 |
| C | -2,3818 | 1,925605 | -1,03456 |
| C | 0,164205 | 2,457966 | 2,858536 |
| C | 2,48682 | 0,835516 | 2,29911 |
| C | -1,87481 | 2,22142 | 0,361006 |
| C | 2,375752 | 1,579986 | 3,483469 |
| C | 1,236291 | 2,372677 | 3,76017 |
| C | -2,63842 | 2,962571 | -1,93844 |
| C | -2,55126 | 0,570481 | -1,43485 |
| C | -3,20667 | 1,295567 | -3,66242 |
| C | 2,097129 | -0,53662 | -0,60858 |
| C | -2,94965 | 0,271218 | -2,75692 |
| C | -3,06359 | 2,655067 | -3,25775 |
| H | 3,194791 | 1,542473 | 4,21759 |
| H | 1,189968 | 2,93546 | 4,704204 |
| C | 2,290697 | -1,91784 | -0,04593 |
| C | 1,613393 | -2,46483 | 0,996811 |
| C | 1,781309 | -3,80982 | 1,554607 |
| C | 1,044682 | -4,15969 | 2,717492 |
| H | -0,71828 | 3,075549 | 3,079697 |
| H | 3,369675 | 0,214147 | 2,094338 |
| H | -1,76181 | 3,312867 | 0,52041 |
| H | -2,58205 | 1,842985 | 1,132466 |
| H | -2,49047 | 4,005873 | -1,62645 |
| H | -2,45236 | -0,23982 | -0,69871 |
| H | -3,53826 | 1,093717 | -4,69108 |
| H | 3,077388 | -0,01661 | -0,69741 |
| H | 1,690066 | -0,59337 | -1,64383 |
| H | -3,08511 | -0,77632 | -3,06437 |
| O | -3,34045 | 3,568657 | -4,19984 |
| H | 3,057566 | -2,50502 | -0,57961 |
| H | 0,852814 | -1,84587 | 1,50533 |
| C | 2,649175 | -4,78529 | 0,991353 |
| C | 1,173145 | -5,42608 | 3,300876 |
| H | 0,36811 | -3,41514 | 3,167504 |
| H | 3,226102 | -4,55356 | 0,083453 |
| C | 2,773524 | -6,05068 | 1,572067 |
| C | 2,038816 | -6,37712 | 2,730132 |
| H | 3,446075 | -6,79602 | 1,121 |
| H | 2,14169 | -7,37388 | 3,185189 |
| H | 0,599105 | -5,67514 | 4,205982 |

| | | | |
|---|---|---|---|
| C | -3,2507 | 4,966167 | -3,90364 |
| H | -2,21165 | 5,251218 | -3,63577 |
| H | -3,94122 | 5,244906 | -3,08029 |
| H | -3,54853 | 5,491343 | -4,82807 |

### 1-(3,5-Dimethoxy-benzyl)-3-cinnamyl-benzimidazolium bromide (3d)

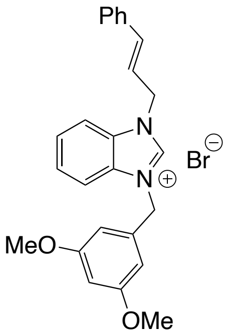


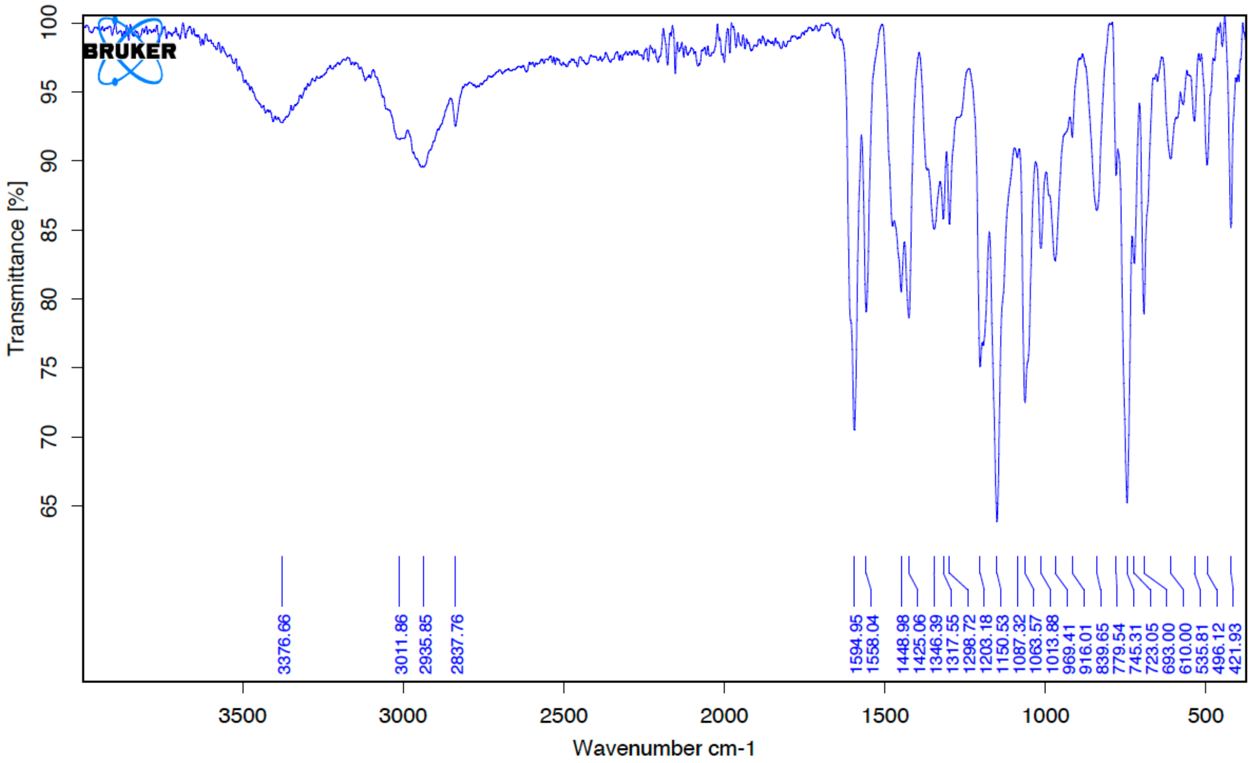


**Figure S13**. FT-IR spectrum

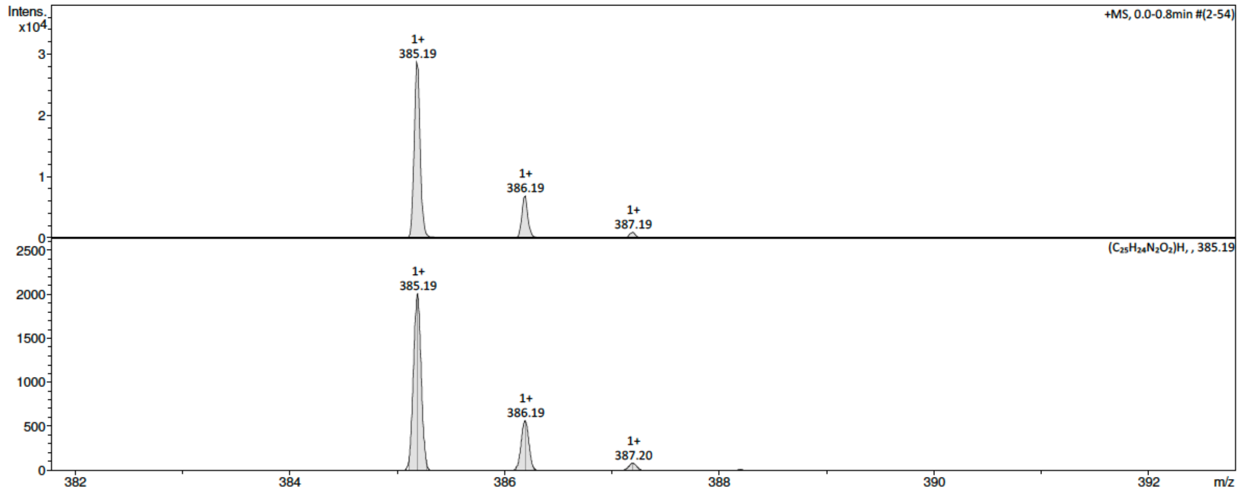


**Figure S14**. Mass spectrum (ESI-TOF): exp. spectrum (top);

calc. spectrum (bottom) for $C_{25}H_{25}N_2O_2$ ($[M - Br]^+$)

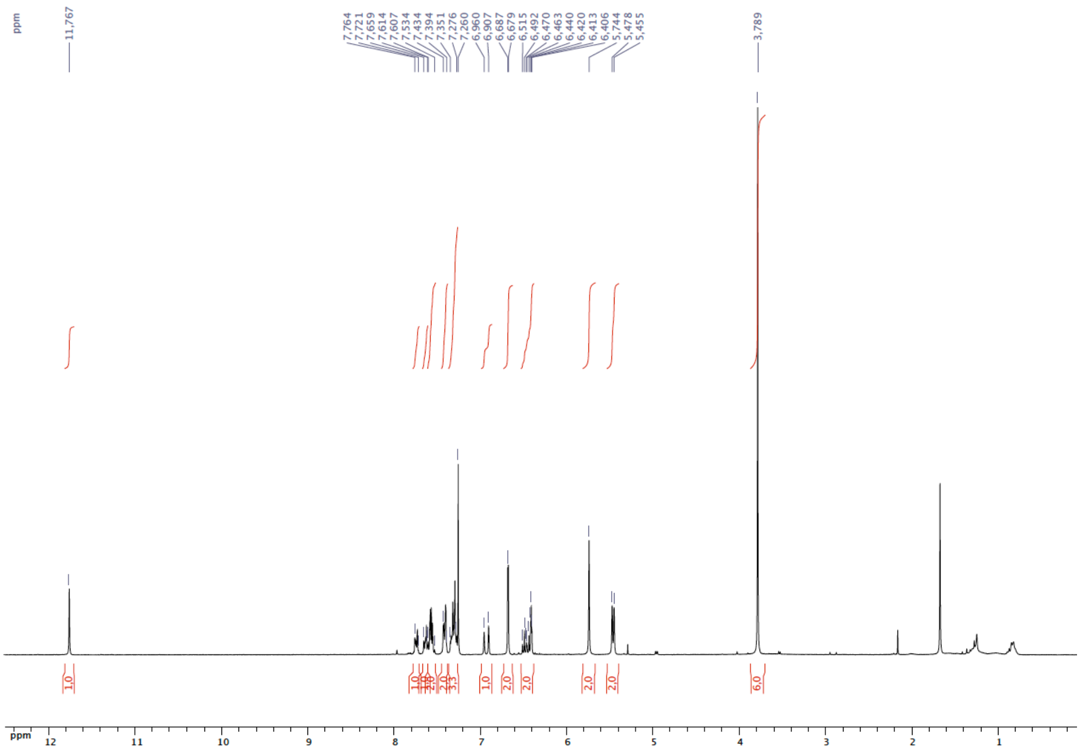


**Figure S15**. $^{1}$H NMR spectrum ($CDCl_3$)

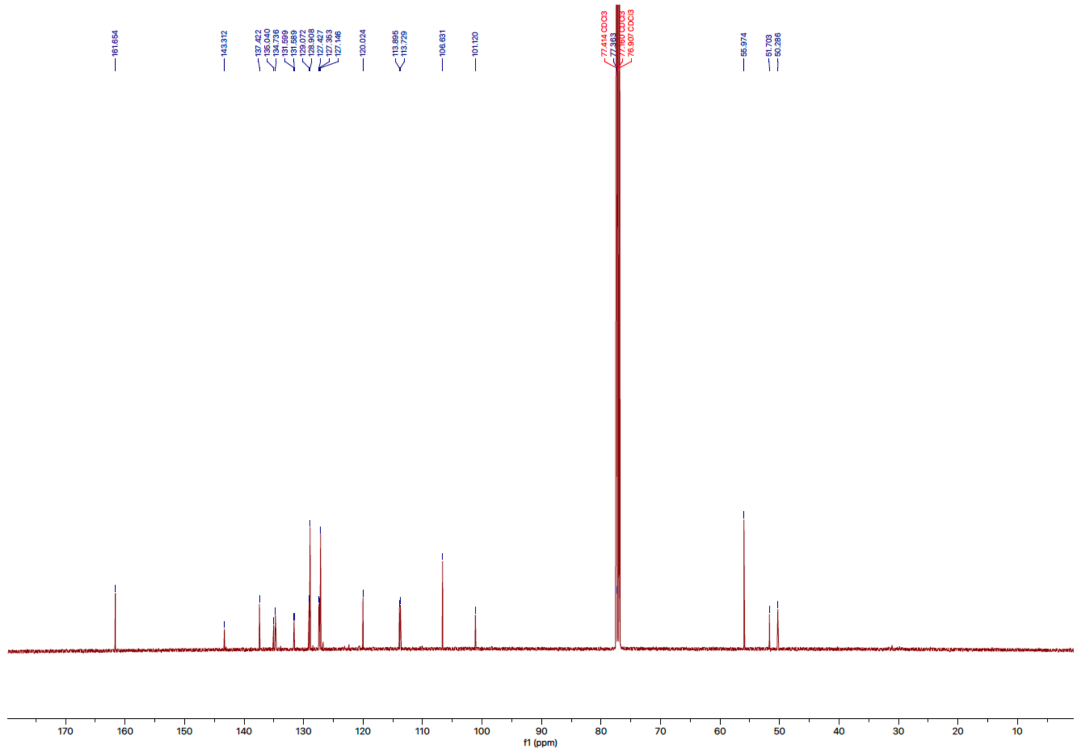


**Figure S16**. $^{13}$C{$^{1}$H} NMR spectrum ($CDCl_3$)

**Table S4.** Optimized Cartesian Coordinates of **3d**

| Atom | Cartesian Coordinates | | |
|---|---|---|---|
| | x | y | z |
| N | 0,006963 | 2,594592 | 0,936773 |
| N | 1,40191 | 1,171855 | 1,843857 |
| C | 0,619894 | 3,28092 | 1,998333 |
| C | 1,513818 | 2,356685 | 2,593875 |
| C | 0,484888 | 1,327891 | 0,85576 |
| C | -1,80571 | 2,042547 | -0,6073 |
| C | 0,47941 | 4,590079 | 2,487859 |
| C | 2,29133 | 2,705204 | 3,710773 |
| C | -1,02073 | 3,157197 | 0,042883 |
| C | 2,145946 | 4,011477 | 4,201851 |
| C | 1,259154 | 4,936439 | 3,601346 |
| C | -1,10774 | 1,106575 | -1,41899 |
| C | -3,16216 | 1,88426 | -0,35867 |
| C | -3,19275 | -0,17583 | -1,70808 |
| C | 2,116131 | -0,08318 | 2,117021 |
| C | -3,86955 | 0,776303 | -0,92833 |
| C | -1,81227 | -0,01431 | -1,9538 |
| C | 2,266065 | -0,92717 | 0,884903 |
| C | 1,848355 | -2,21891 | 0,809829 |
| C | 2,059857 | -3,15561 | -0,29846 |
| C | 1,357869 | -4,38942 | -0,29687 |
| O | -5,17997 | 0,736464 | -0,64004 |
| H | -7,0118 | -0,1358 | -0,7832 |
| O | -1,24677 | -0,94729 | -2,73851 |
| H | 0,352141 | -1,75001 | -3,69982 |
| C | 2,954085 | -2,89396 | -1,37185 |
| C | 1,52252 | -5,31605 | -1,33539 |
| C | 3,120598 | -3,82195 | -2,40678 |
| C | 2,402639 | -5,03456 | -2,39587 |
| C | 0,14557 | -0,87827 | -3,05422 |
| C | -5,98748 | -0,3317 | -1,14566 |
| H | -0,20377 | 5,316255 | 2,024667 |
| H | 2,980719 | 1,993707 | 4,186729 |
| H | -0,52596 | 3,806037 | -0,71364 |
| H | -1,68828 | 3,799316 | 0,649726 |
| H | 2,734505 | 4,322788 | 5,077501 |
| H | 1,178336 | 5,951213 | 4,017987 |
| H | -0,07434 | 1,307419 | -1,72397 |
| H | -3,72527 | 2,581055 | 0,27911 |
| H | -3,69595 | -1,04143 | -2,15669 |
| H | 1,591242 | -0,63662 | 2,924544 |
| H | 3,118684 | 0,197427 | 2,508441 |
| H | 2,822593 | -0,46688 | 0,050367 |
| H | 1,284444 | -2,62848 | 1,667852 |
| H | 0,674715 | -4,61771 | 0,537105 |

| | | | |
|---|---|---|---|
| H | 3,549108 | -1,96758 | -1,37701 |
| H | 0,969828 | -6,26747 | -1,31475 |
| H | 3,829129 | -3,6106 | -3,22267 |
| H | 2,54318 | -5,76569 | -3,207 |
| H | 0,767072 | -0,94675 | -2,13698 |
| H | 0,387116 | 0,053341 | -3,60987 |
| H | -5,63831 | -1,31189 | -0,75884 |
| H | -5,9817 | -0,34451 | -2,25604 |

**1-(Naphthalen-1-ylmethyl)-3-cinnamyl-benzimidazolium bromide (3e)**

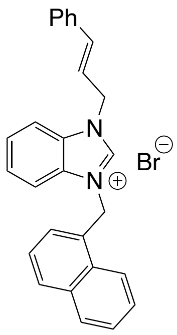


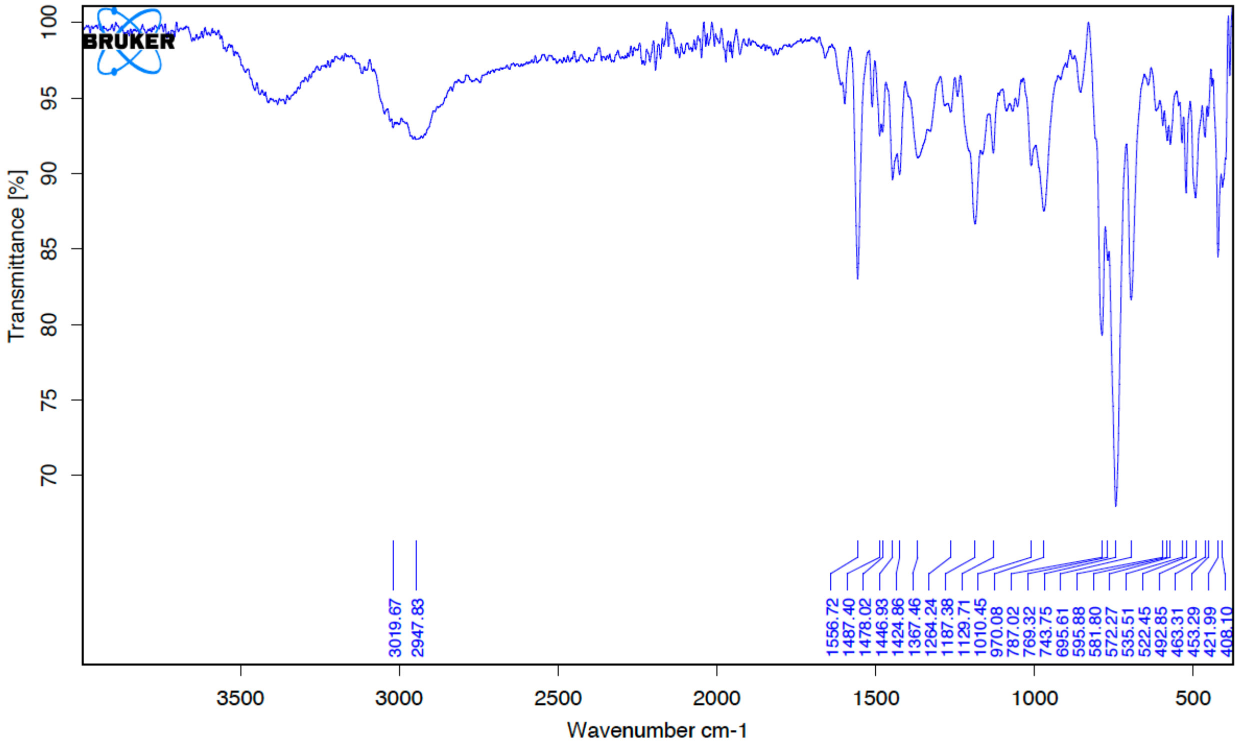


**Figure S17**. FT-IR spectrum

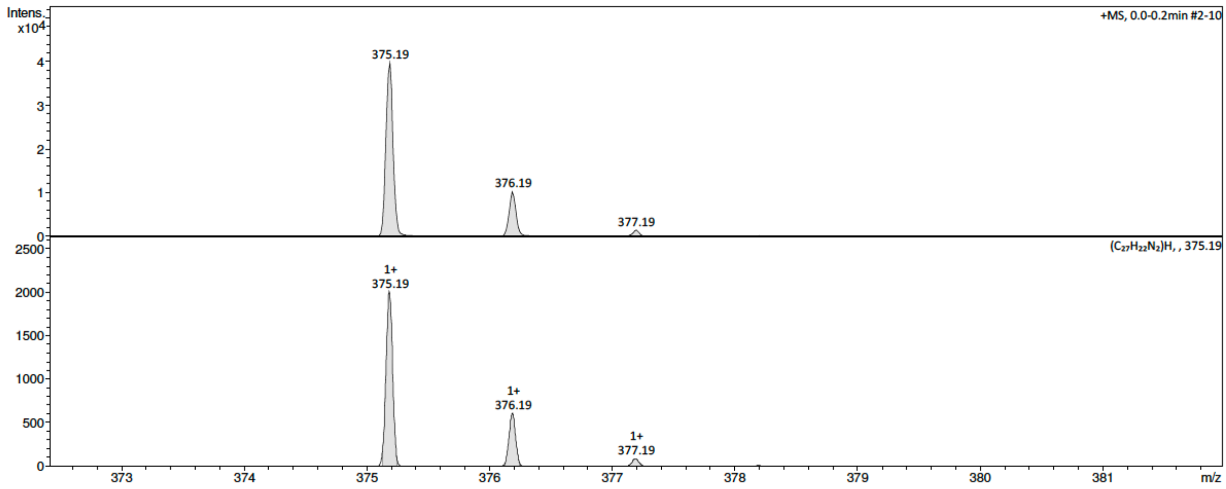


**Figure S18**. Mass spectrum (ESI-TOF): exp. spectrum (top);

calc. spectrum (bottom) for $C_{27}H_{23}N_2$ ($[M - Br]^+$)

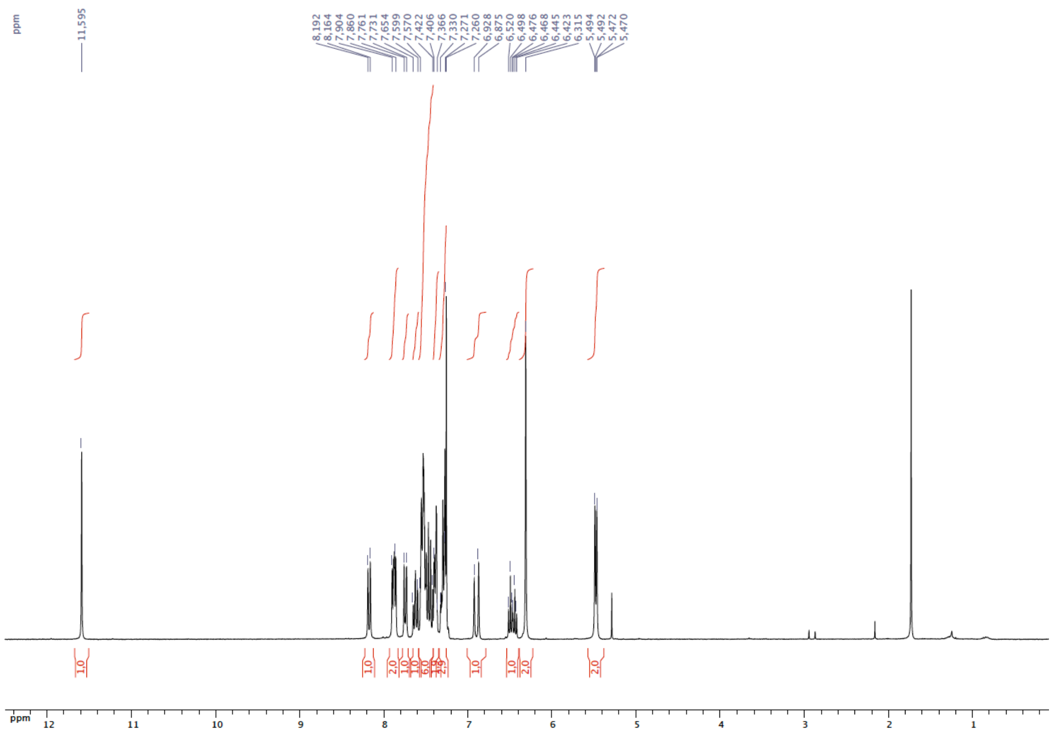

**Figure S19**. $^{1}H$ NMR spectrum ($CDCl_3$)

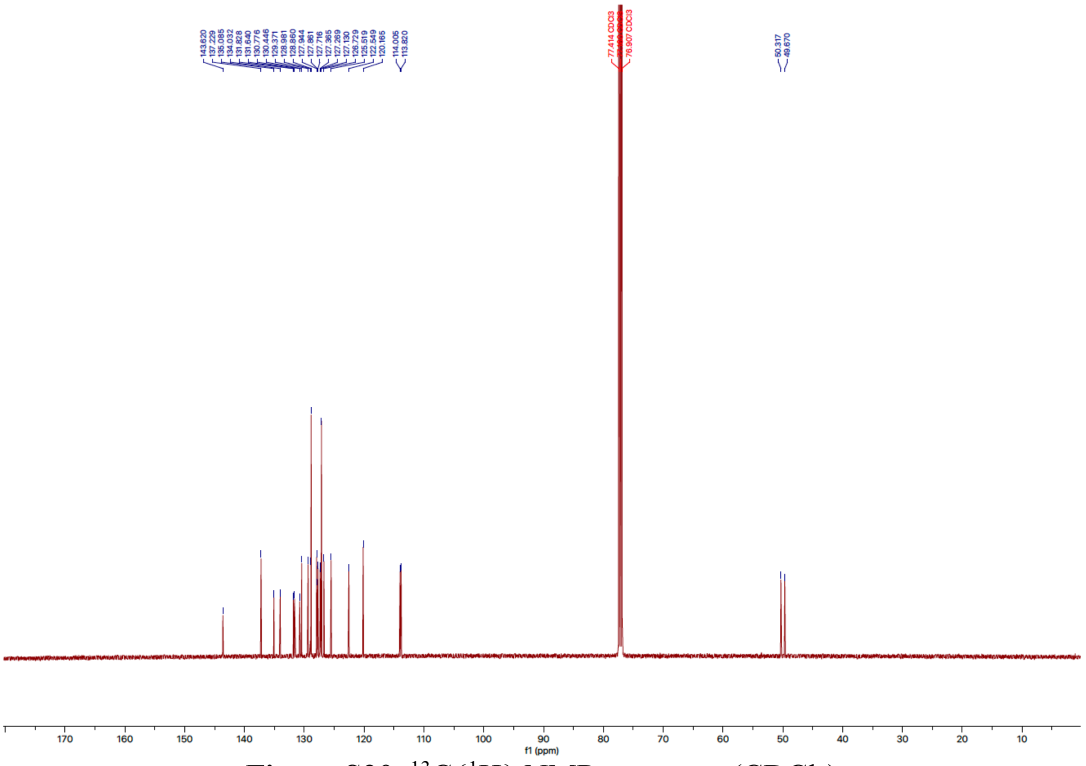

**Figure S20**. $^{13}C\{^{1}H\}$ NMR spectrum ($CDCl_3$)

**Table S5.** Optimized Cartesian Coordinates of **3e**

| **Atom** | **Cartesian Coordinates** | | |
|---|---|---|---|
| | **x** | **y** | **z** |
| N | -0,27335 | 1,909461 | 0,479975 |
| N | 1,481495 | 0,767178 | -0,15097 |
| C | 0,685907 | 2,099979 | 1,488035 |
| C | 1,828418 | 1,365723 | 1,076897 |
| C | 0,213456 | 1,099616 | -0,49463 |
| C | -2,22773 | 2,224982 | -0,94958 |
| C | 0,670323 | 2,829797 | 2,688228 |
| C | 2,999834 | 1,342332 | 1,852312 |
| C | -1,60816 | 2,507846 | 0,407515 |
| C | 2,986269 | 2,080755 | 3,04546 |
| C | 1,843851 | 2,808177 | 3,457612 |
| C | -1,63521 | 2,824512 | -2,0815 |
| C | -3,40448 | 1,414427 | -1,10849 |
| C | -3,29547 | 1,854086 | -3,55429 |
| C | 2,343194 | -0,05657 | -0,99904 |
| C | -3,94162 | 1,235112 | -2,44238 |
| C | -2,15705 | 2,635529 | -3,37761 |
| H | 3,887004 | 2,092667 | 3,677214 |
| H | 1,876244 | 3,369391 | 4,402982 |
| C | 2,711354 | -1,40619 | -0,45019 |
| C | 2,191423 | -1,99567 | 0,659358 |
| C | 2,535582 | -3,31069 | 1,203525 |
| C | 1,899542 | -3,73829 | 2,400615 |
| H | -0,21418 | 3,396 | 3,013969 |
| H | 3,885996 | 0,771273 | 1,543002 |
| H | -1,51058 | 3,605257 | 0,557781 |
| H | -2,22532 | 2,123175 | 1,24209 |
| H | -0,75675 | 3,473971 | -1,94422 |
| C | -4,07987 | 0,774743 | -0,025 |
| H | -3,72066 | 1,711132 | -4,55993 |
| H | 3,26385 | 0,522958 | -1,23565 |
| H | 1,800451 | -0,16763 | -1,96618 |
| C | -5,11181 | 0,444588 | -2,62444 |
| H | -1,67234 | 3,116635 | -4,23938 |
| H | 3,464727 | -1,9356 | -1,05834 |
| H | 1,426697 | -1,44266 | 1,232946 |
| C | 3,479444 | -4,18482 | 0,595725 |
| C | 2,192636 | -4,98281 | 2,969759 |
| H | 1,167603 | -3,07218 | 2,885293 |
| H | 3,986942 | -3,88895 | -0,33422 |
| C | 3,769432 | -5,42791 | 1,163696 |
| C | 3,129304 | -5,83324 | 2,353041 |
| H | 4,50023 | -6,09344 | 0,679598 |
| H | 3,362421 | -6,81282 | 2,796686 |
| H | 1,693091 | -5,29436 | 3,899109 |

| | | | |
|---|---|---|---|
| H | -3,69875 | 0,879241 | 1,001005 |
| C | -5,2262 | 0,008931 | -0,23522 |
| H | -5,51058 | 0,317039 | -3,64267 |
| C | -5,74868 | -0,15756 | -1,54 |
| H | -5,72745 | -0,46692 | 0,620589 |
| H | -6,65646 | -0,75883 | -1,69699 |

## 1-(Pyren-1-ylmethyl)-3-cinnamyl-benzimidazolium bromide (3f)

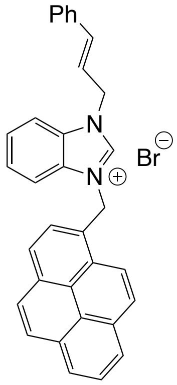


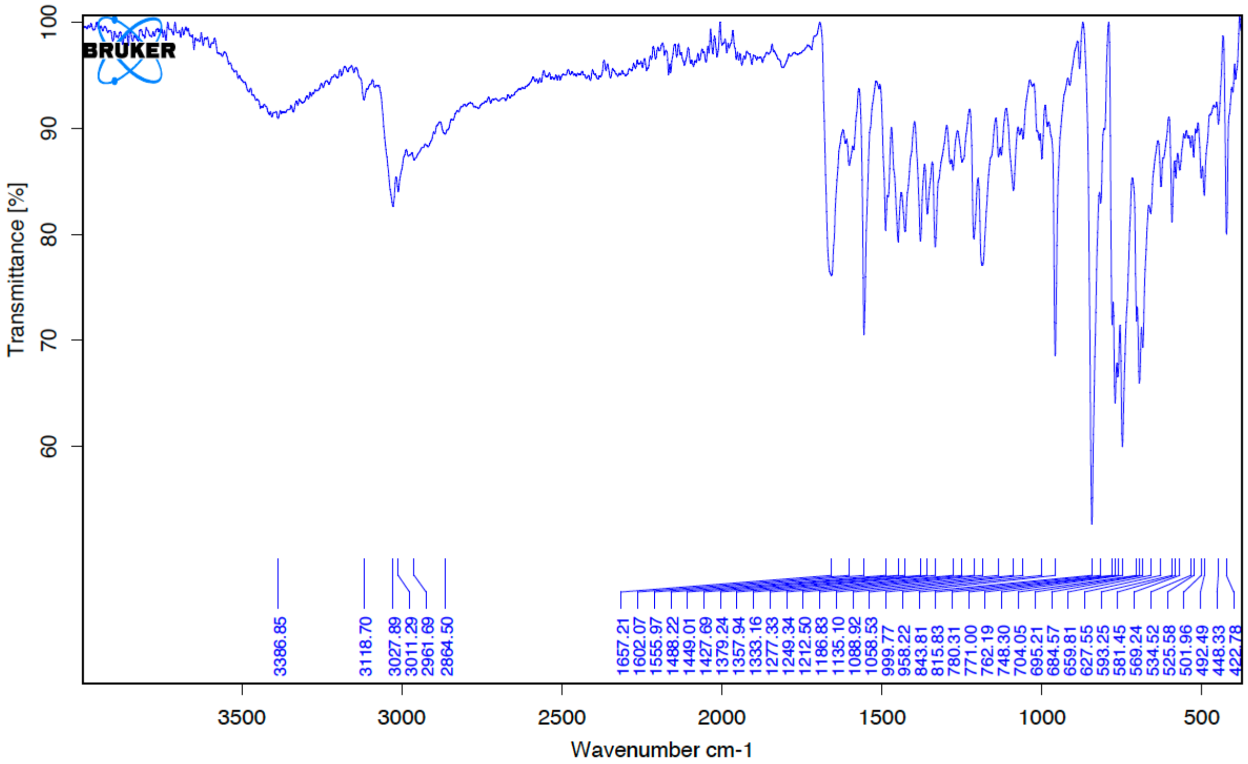


**Figure S21**. FT-IR spectrum

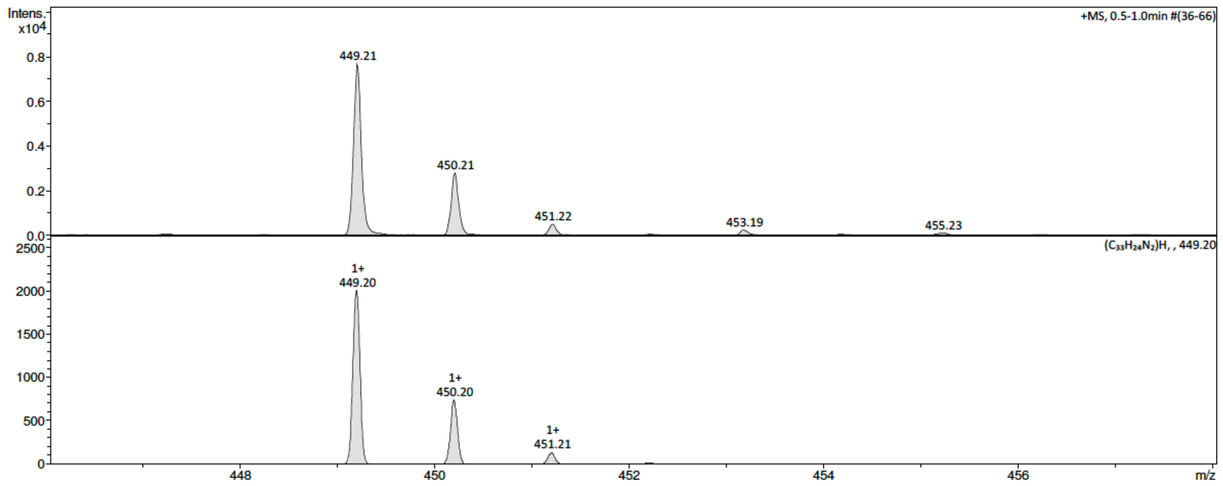


**Figure S22**. Mass spectrum (ESI-TOF): exp. spectrum (top);

calc. spectrum (bottom) for $C_{33}H_{25}N_2$ ($[M - Br]^+$)

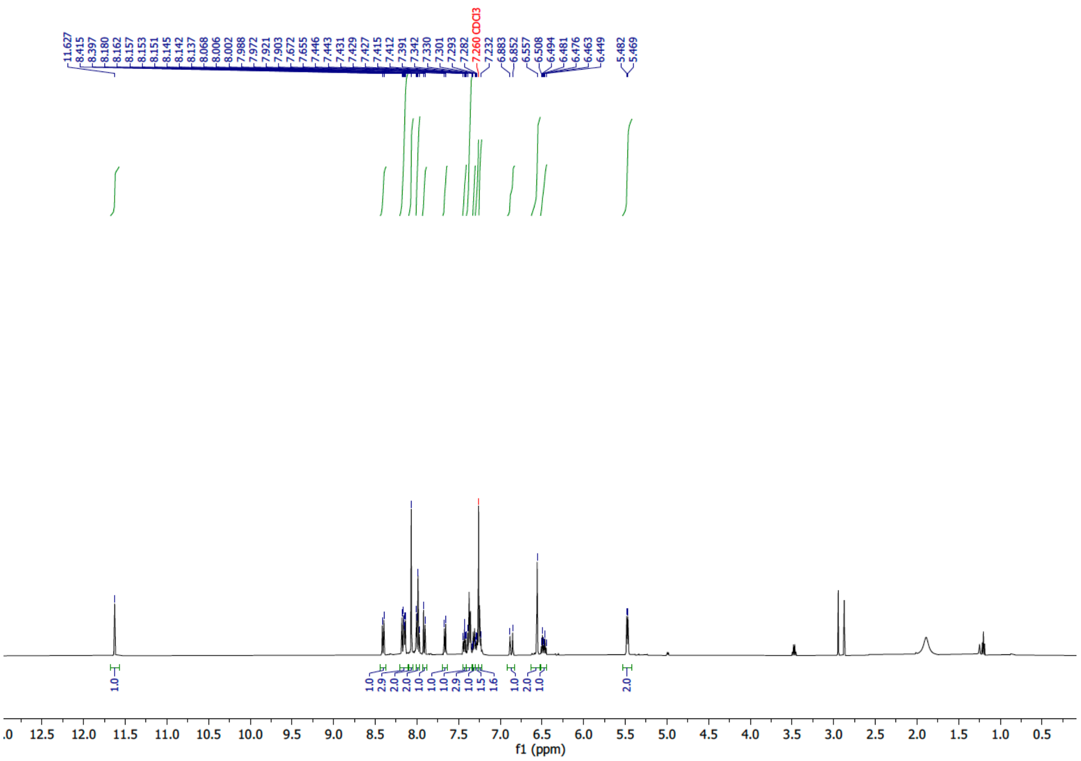

**Figure S23**. $^{1}H$ NMR spectrum ($CDCl_3$)

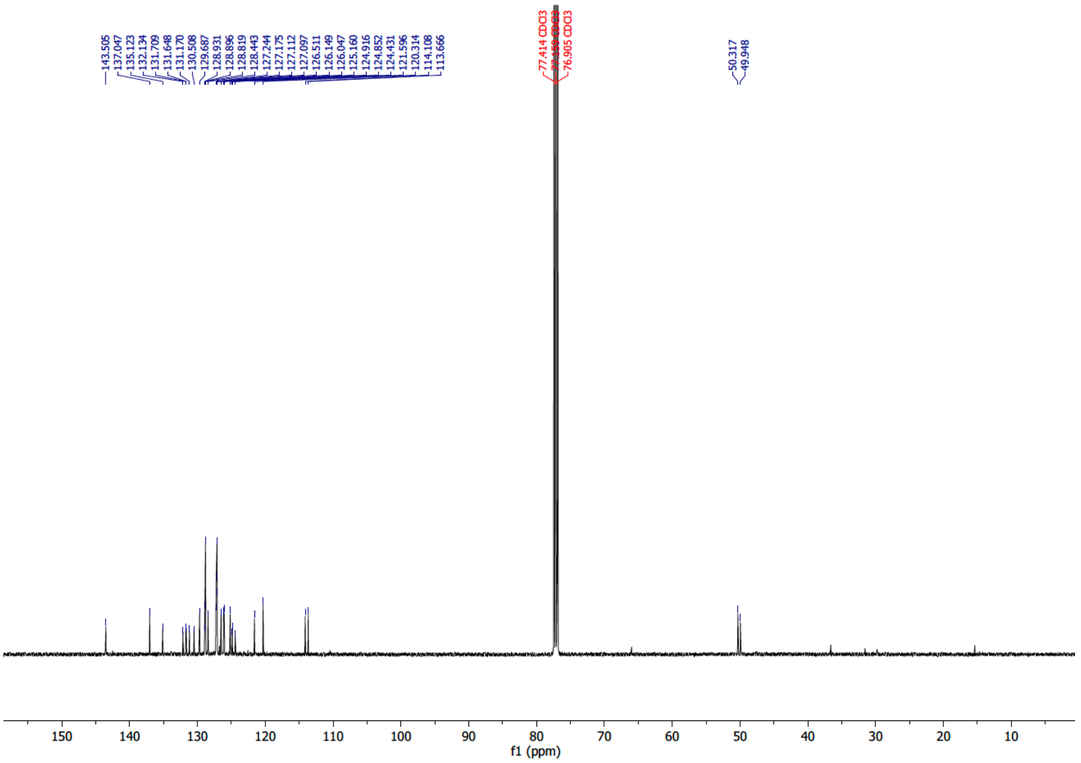

**Figure S24**. $^{13}C\{^{1}H\}$ NMR spectrum ($CDCl_3$)

**Table S6.** Optimized Cartesian Coordinates of **3f**

| Atom | Cartesian Coordinates | | |
|---|---|---|---|
| | x | y | z |
| N | -0,34485 | 0,938985 | 1,616411 |
| N | 1,476162 | -0,12878 | 1,069238 |
| C | 0,496659 | 1,035478 | 2,735832 |
| C | 1,683235 | 0,344951 | 2,378924 |
| C | 0,248349 | 0,233783 | 0,614323 |
| C | -2,16023 | 1,334552 | 0,034355 |
| C | 0,347476 | 1,644588 | 3,992704 |
| C | 2,766629 | 0,248456 | 3,268016 |
| C | -1,69894 | 1,473715 | 1,474294 |
| C | 2,619962 | 0,865568 | 4,520302 |
| C | 1,433437 | 1,549045 | 4,877453 |
| C | -1,7968 | 2,302594 | -0,96794 |
| C | -3,02776 | 0,27299 | -0,30488 |
| C | -3,1656 | 1,037928 | -2,62761 |
| C | 2,439699 | -0,86639 | 0,253661 |
| C | -3,51543 | 0,116672 | -1,60149 |
| C | -2,30473 | 2,143827 | -2,3048 |
| H | 3,447586 | 0,811605 | 5,243577 |
| H | 1,359358 | 2,014292 | 5,871388 |
| C | 2,712475 | -2,28256 | 0,6814 |
| C | 2,050031 | -2,97375 | 1,64431 |
| C | 2,288581 | -4,35808 | 2,064251 |
| C | 1,535693 | -4,87769 | 3,150558 |
| H | -0,57454 | 2,17157 | 4,277402 |
| H | 3,685699 | -0,2918 | 3,001756 |
| H | -1,70709 | 2,533258 | 1,805038 |
| H | -2,3859 | 0,919128 | 2,147483 |
| C | -0,96038 | 3,433527 | -0,69027 |
| H | -3,31715 | -0,44851 | 0,474325 |
| C | -3,65698 | 0,899832 | -3,96541 |
| H | 3,390144 | -0,2879 | 0,217359 |
| H | 2,03385 | -0,85087 | -0,78345 |
| H | -4,1812 | -0,72627 | -1,84281 |
| C | -1,9666 | 3,092676 | -3,32126 |
| H | 3,523839 | -2,76613 | 0,110699 |
| H | 1,240685 | -2,45802 | 2,190656 |
| C | 3,238858 | -5,21114 | 1,439678 |
| C | 1,726059 | -6,18986 | 3,60097 |
| H | 0,795098 | -4,22956 | 3,646176 |
| H | 3,832223 | -4,84571 | 0,587939 |
| C | 3,426468 | -6,52194 | 1,88813 |
| C | 2,673346 | -7,01779 | 2,971634 |
| H | 4,165007 | -7,16912 | 1,390839 |
| H | 2,826254 | -8,04942 | 3,322753 |
| H | 1,136159 | -6,57153 | 4,448058 |

| | | | |
|---|---|---|---|
| C | -0,63761 | 4,353358 | -1,67261 |
| C | -1,12376 | 4,212714 | -3,01229 |
| C | -2,47329 | 2,93367 | -4,65729 |
| H | 0,004811 | 5,213706 | -1,42839 |
| C | -3,32137 | 1,815304 | -4,94835 |
| H | -4,3127 | 0,048112 | -4,20374 |
| C | -2,12199 | 3,889869 | -5,64843 |
| H | -0,55657 | 3,575746 | 0,32205 |
| H | -3,7092 | 1,695087 | -5,97156 |
| C | -1,29505 | 4,980569 | -5,33922 |
| C | -0,80176 | 5,144481 | -4,03747 |
| H | -0,15687 | 6,003481 | -3,79464 |
| H | -2,51131 | 3,765632 | -6,67079 |
| H | -1,03479 | 5,710148 | -6,12044 |

**Bromo(1-benzyl-3-cinnamyl-benzimidazol-2-ylidene)silver (I) (1a)**

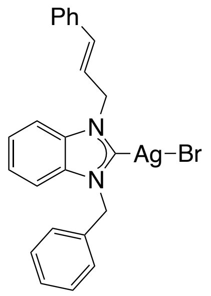


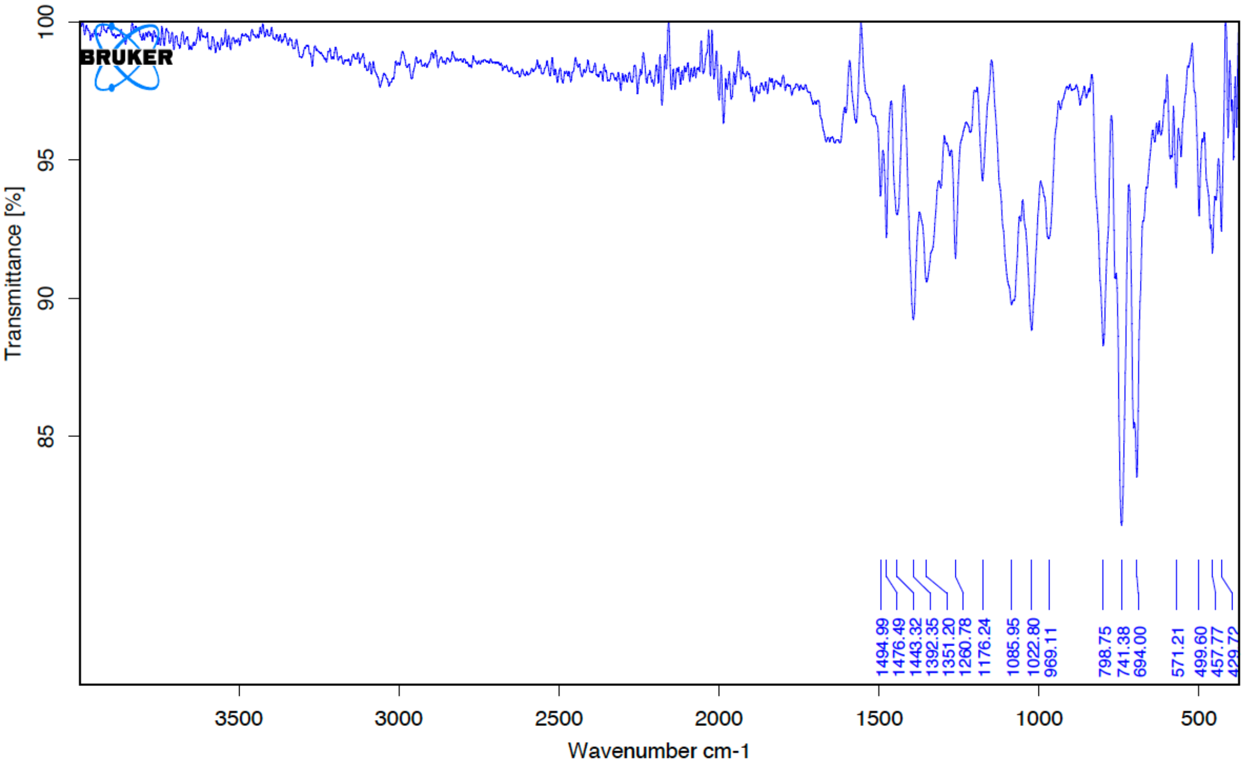


**Figure S25**. FT-IR spectrum

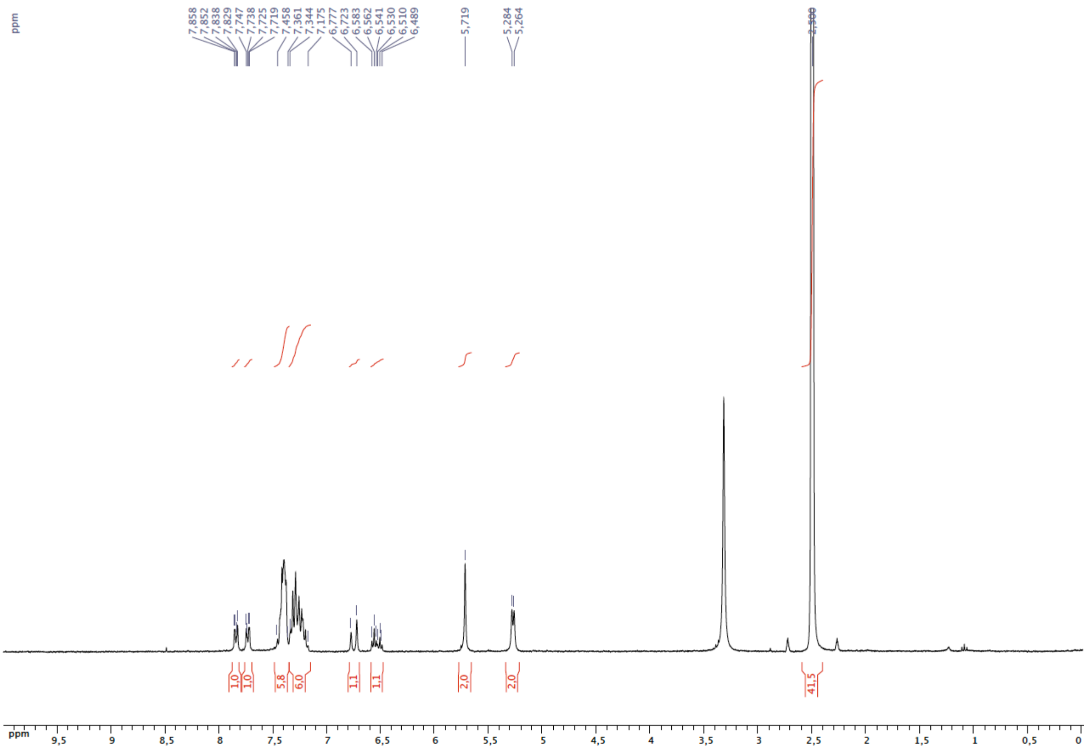


**Figure S26**. $^{1}$H NMR spectrum (DMSO-$d_6$)

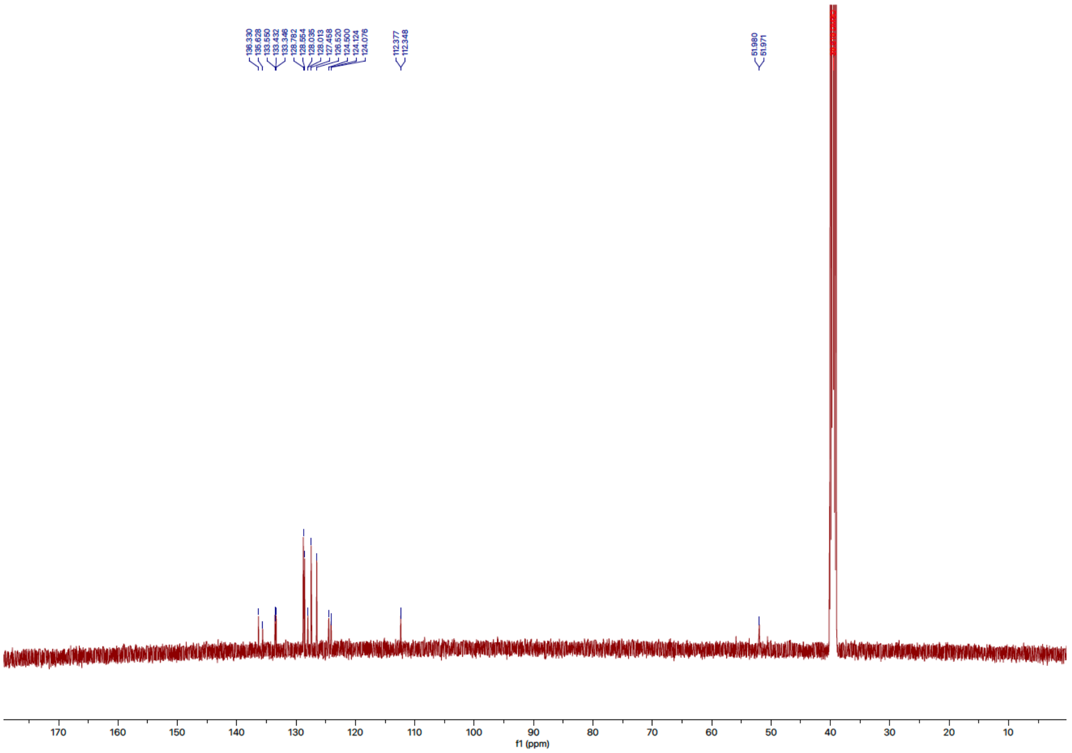


**Figure S27**. $^{13}$C{$^{1}$H} NMR spectrum (DMSO-$d_6$)

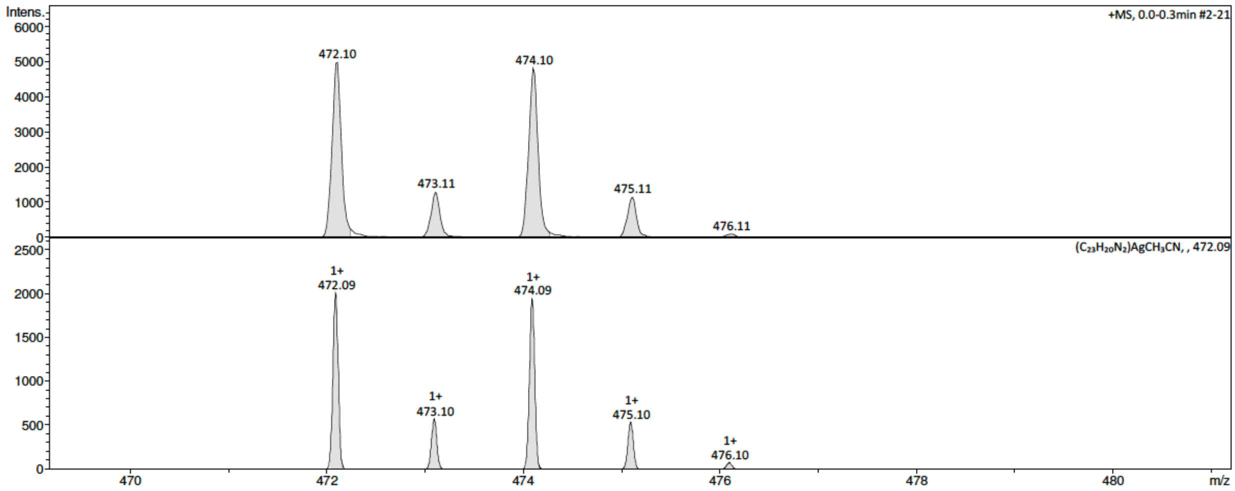


**Figure S28**. Mass spectrum (ESI-TOF): exp. spectrum (top); calc. spectrum (bottom) for $C_{25}H_{23}N_3Ag$ ($[M - Br + CH_3CN]^+$)

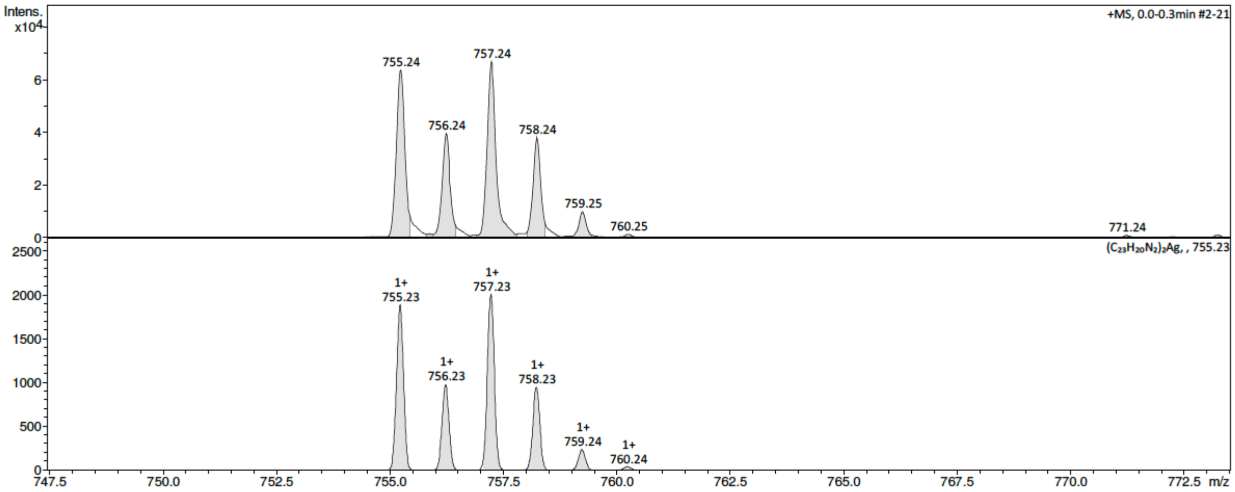


**Figure S29**. Mass spectrum (ESI-TOF): exp. spectrum (top); calc. spectrum (bottom) for $C_{46}H_{40}N_4Ag$ ($[M - Br + C_{23}H_{20}N_2]^+$)

**Table S7.** Optimized Cartesian Coordinates of **1a**

| **Atom** | **Cartesian Coordinates** | | |
|---|---|---|---|
| | **x** | **y** | **z** |
| Ag | -1,33501 | -0,0019 | -2,98634 |
| Br | -2,60137 | -0,33355 | -5,16388 |
| N | -1,04921 | 1,440283 | -0,20328 |
| N | 0,813352 | 0,406804 | -0,69877 |
| C | -0,15418 | 1,734398 | 0,836399 |
| C | 1,055763 | 1,072421 | 0,511407 |
| C | -0,46261 | 0,628883 | -1,13147 |
| C | -3,04229 | 2,021928 | -1,6234 |
| C | -0,27777 | 2,502624 | 2,007084 |
| C | 2,191276 | 1,165063 | 1,333847 |

| | | | |
|---|---|---|---|
| C | -2,45117 | 1,896998 | -0,2328 |
| C | 2,068579 | 1,941705 | 2,494998 |
| C | 0,856609 | 2,595025 | 2,82687 |
| C | -2,48391 | 2,925773 | -2,56933 |
| C | -4,2021 | 1,295107 | -1,9674 |
| C | -4,20318 | 2,321451 | -4,1805 |
| C | 1,794171 | -0,36737 | -1,45484 |
| C | -4,78034 | 1,442167 | -3,23723 |
| C | -3,0593 | 3,072077 | -3,83505 |
| H | 2,934437 | 2,04403 | 3,16623 |
| H | 0,802666 | 3,188484 | 3,75157 |
| C | 2,22142 | -1,66988 | -0,84272 |
| C | 1,628169 | -2,29427 | 0,213362 |
| C | 2,017959 | -3,56756 | 0,812562 |
| C | 1,255769 | -4,06464 | 1,90747 |
| H | -1,21462 | 3,009425 | 2,277804 |
| H | 3,129824 | 0,650762 | 1,085787 |
| H | -2,47268 | 2,887691 | 0,267253 |
| H | -3,06445 | 1,208359 | 0,385064 |
| H | -1,59546 | 3,519672 | -2,30104 |
| H | -4,65231 | 0,604678 | -1,23683 |
| H | -4,66344 | 2,442393 | -5,17219 |
| H | 2,683905 | 0,272195 | -1,65133 |
| H | 1,33382 | -0,54886 | -2,45791 |
| H | -5,68066 | 0,86852 | -3,50112 |
| H | -2,62066 | 3,770671 | -4,56295 |
| H | 3,081938 | -2,13604 | -1,35185 |
| H | 0,757984 | -1,80422 | 0,684035 |
| C | 3,125496 | -4,33976 | 0,356226 |
| C | 1,580774 | -5,27895 | 2,518645 |
| H | 0,39741 | -3,47876 | 2,27242 |
| H | 3,737878 | -3,98496 | -0,48605 |
| C | 3,448015 | -5,55199 | 0,967909 |
| C | 2,678511 | -6,02824 | 2,051046 |
| H | 4,305706 | -6,13776 | 0,604751 |
| H | 2,936941 | -6,98477 | 2,529533 |
| H | 0,981421 | -5,64839 | 3,364093 |

**Chloro[1-methoxymethyl-3-cinnamyl-benzimidazol-2-yliden]silver(I) (1b)**

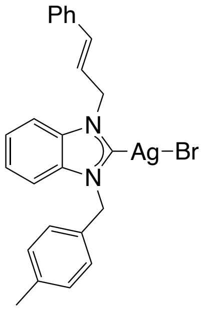


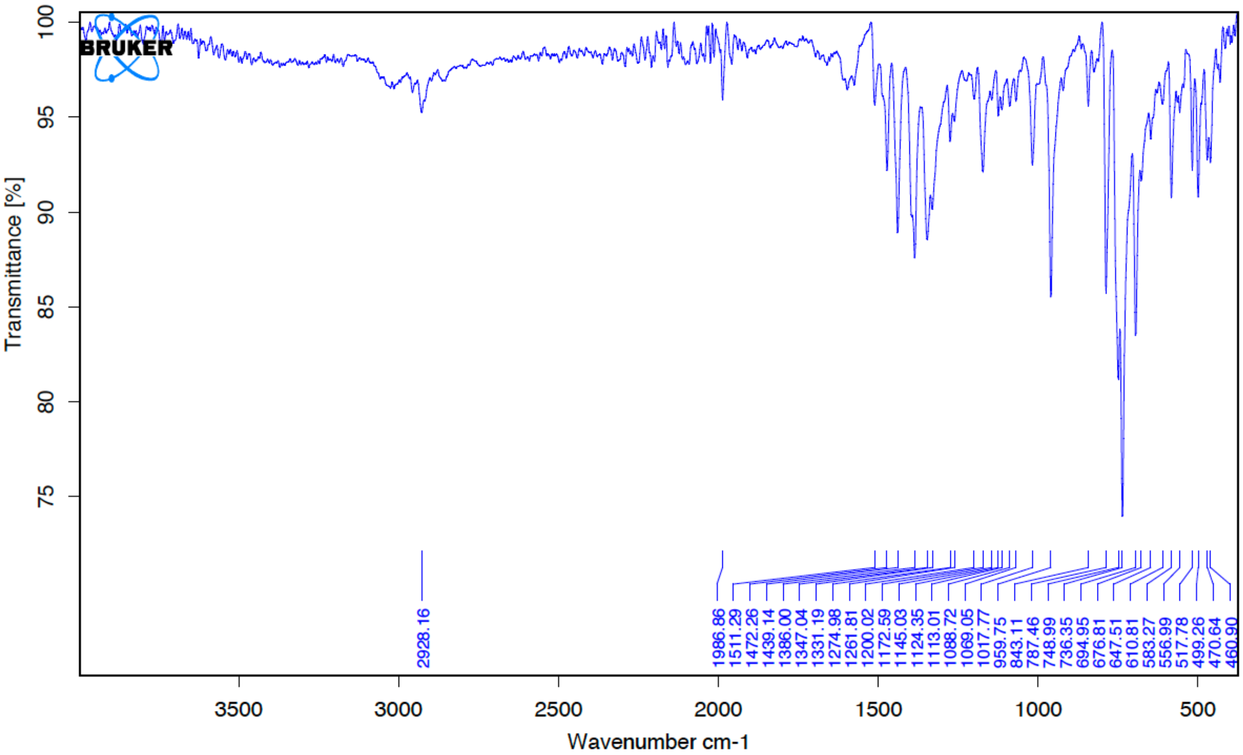


**Figure S30**. FT-IR spectrum

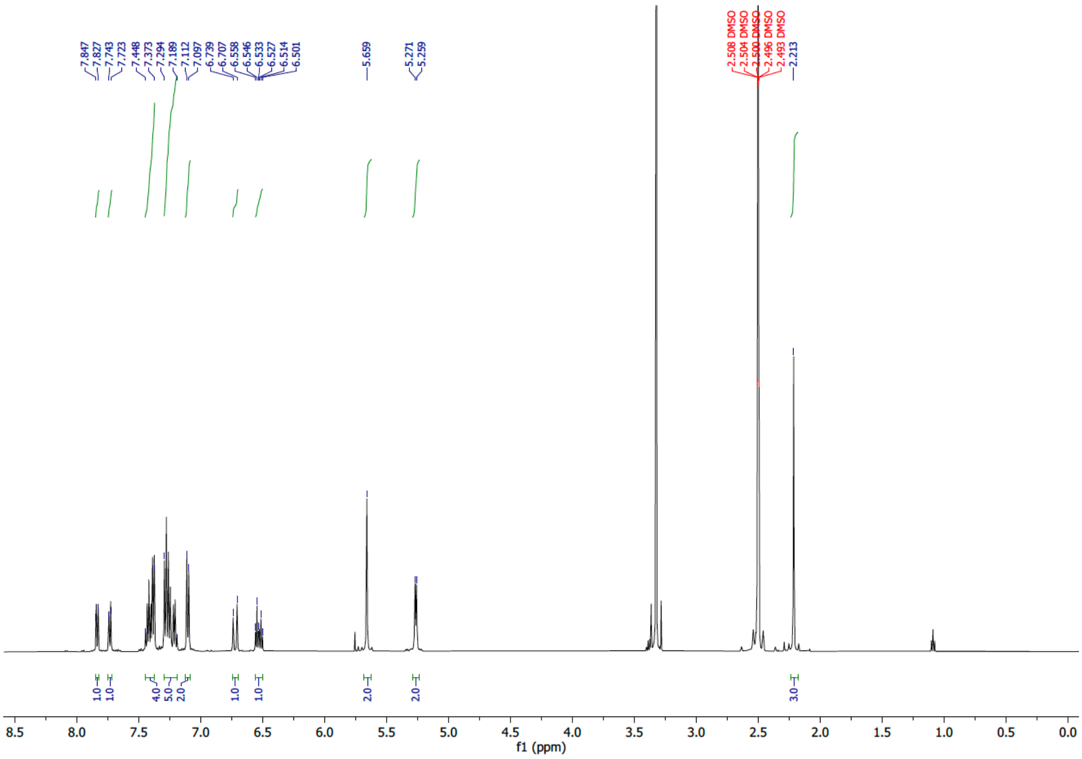


**Figure S31**. $^{1}$H NMR spectrum ((DMSO-$d_6$)

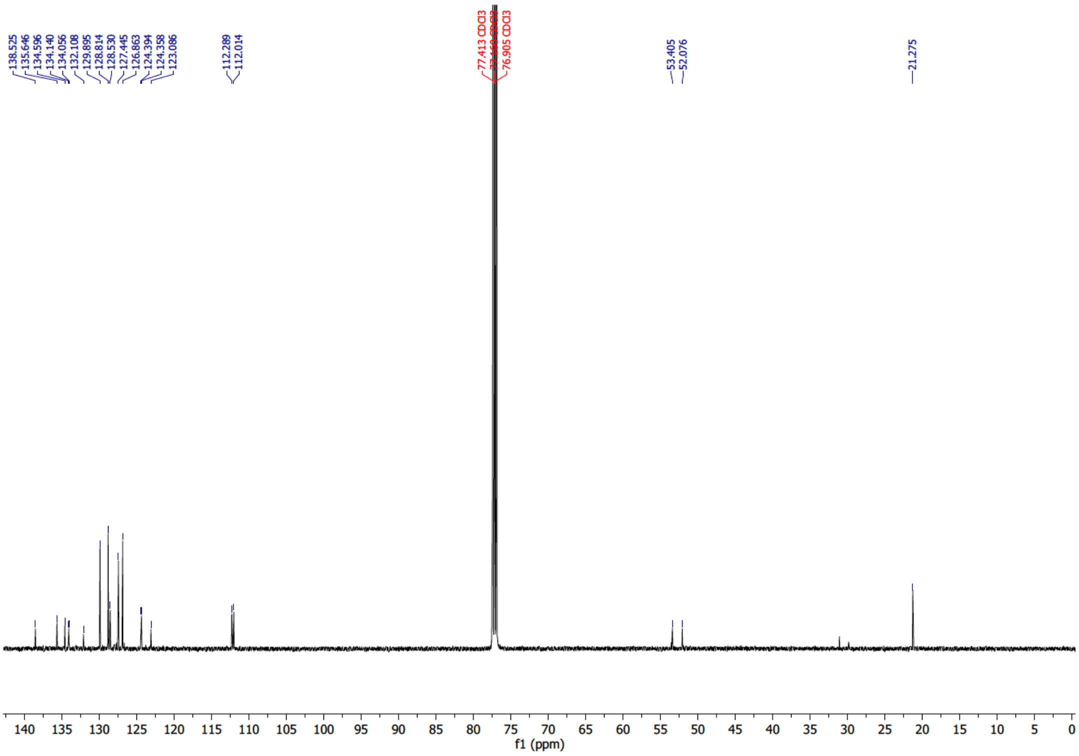


**Figure S32** $^{13}$C{$^{1}$H} NMR spectrum (DMSO-$d_6$)

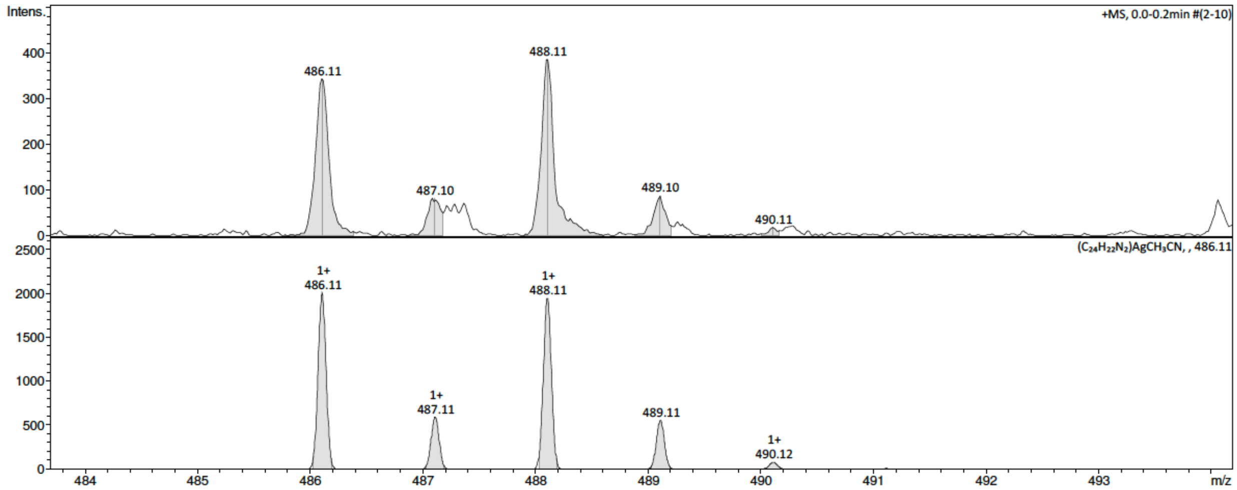


**Figure S33**. Mass spectrum (ESI-TOF): exp. spectrum (top);

calc. spectrum (bottom) for $C_{26}H_{25}N_3Ag$ ($[M - Br + CH_3CN]^+$)

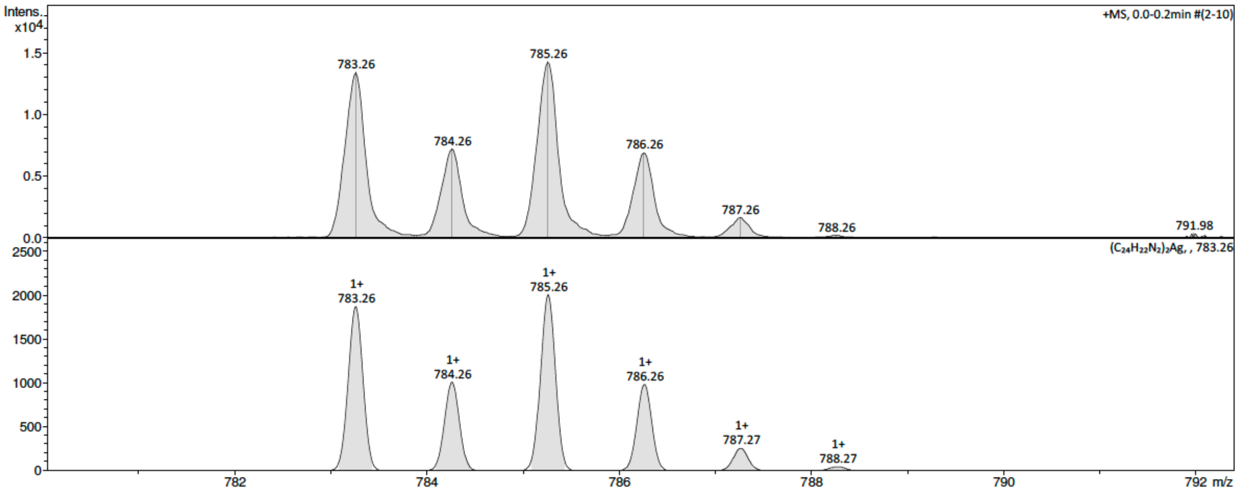


**Figure S34**. Mass spectrum (ESI-TOF): exp. spectrum (top);

calc. spectrum (bottom) for $C_{48}H_{44}N_4Ag$ ($[M - Br + C_{24}H_{22}N_2]^+$)

**Table S8.** Optimized Cartesian Coordinates of **1b**

| **Atom** | **Cartesian Coordinates** | | |
|---|---|---|---|
| | **x** | **y** | **z** |
| Ag | -1,03123 | 0,044113 | -2,24576 |
| Br | -2,48442 | -0,38754 | -4,28293 |
| N | -0,70616 | 1,52439 | 0,497513 |
| N | 1,166829 | 0,51289 | -0,00535 |
| C | 0,191698 | 1,836624 | 1,529429 |
| C | 1,407918 | 1,188576 | 1,199627 |
| C | -0,11453 | 0,714769 | -0,42945 |
| C | -2,7034 | 2,103477 | -0,91743 |
| C | 0,067308 | 2,609593 | 2,696904 |
| C | 2,547116 | 1,299728 | 2,014428 |
| C | -2,11609 | 1,954371 | 0,471781 |

| | | | |
|---|---|---|---|
| C | 2,423136 | 2,080689 | 3,172347 |
| C | 1,205469 | 2,720848 | 3,508742 |
| C | -2,13649 | 3,015255 | -1,85531 |
| C | -3,87269 | 1,408956 | -1,27701 |
| C | -3,89626 | 2,50584 | -3,48383 |
| C | 2,151743 | -0,25982 | -0,75818 |
| C | -4,4537 | 1,597678 | -2,5414 |
| C | -2,72141 | 3,208502 | -3,10619 |
| H | 3,291838 | 2,196231 | 3,837583 |
| H | 1,149813 | 3,318134 | 4,430809 |
| C | 2,585275 | -1,55693 | -0,13682 |
| C | 1,990428 | -2,17891 | 0,918483 |
| C | 2,38242 | -3,4483 | 1,527147 |
| C | 1,615626 | -3,94368 | 2,618613 |
| H | -0,87383 | 3,10613 | 2,971903 |
| H | 3,489778 | 0,794396 | 1,763507 |
| H | -2,15886 | 2,93208 | 0,995816 |
| H | -2,72073 | 1,240541 | 1,06932 |
| H | -1,23292 | 3,585372 | -1,58556 |
| H | -4,33418 | 0,703489 | -0,56819 |
| C | -4,54375 | 2,756462 | -4,81633 |
| H | 3,037636 | 0,384413 | -0,95603 |
| H | 1,692864 | -0,44839 | -1,75988 |
| H | -5,36844 | 1,046265 | -2,80501 |
| H | -2,27096 | 3,923876 | -3,81202 |
| H | 3,451284 | -2,0203 | -0,63912 |
| H | 1,115142 | -1,69131 | 1,382305 |
| C | 3,495775 | -4,21738 | 1,082383 |
| C | 1,941805 | -5,15408 | 3,237932 |
| H | 0,751891 | -3,36041 | 2,975148 |
| H | 4,112428 | -3,86385 | 0,24259 |
| C | 3,819712 | -5,42569 | 1,701978 |
| C | 3,045498 | -5,90069 | 2,781784 |
| H | 4,682568 | -6,00895 | 1,34698 |
| H | 3,304853 | -6,8539 | 3,26638 |
| H | 1,337875 | -5,52187 | 4,080915 |
| H | -3,84087 | 2,529415 | -5,64425 |
| H | -4,82206 | 3,826793 | -4,91917 |
| H | -5,45424 | 2,146024 | -4,95823 |

**Bromo[1-(3-methoxylbenzyl)-3-cinnamyl-benzimidazol-2-yliden]silver(I) (1c)**

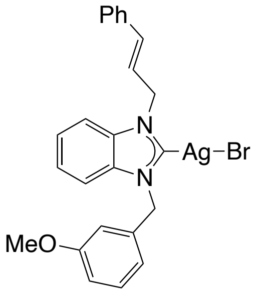


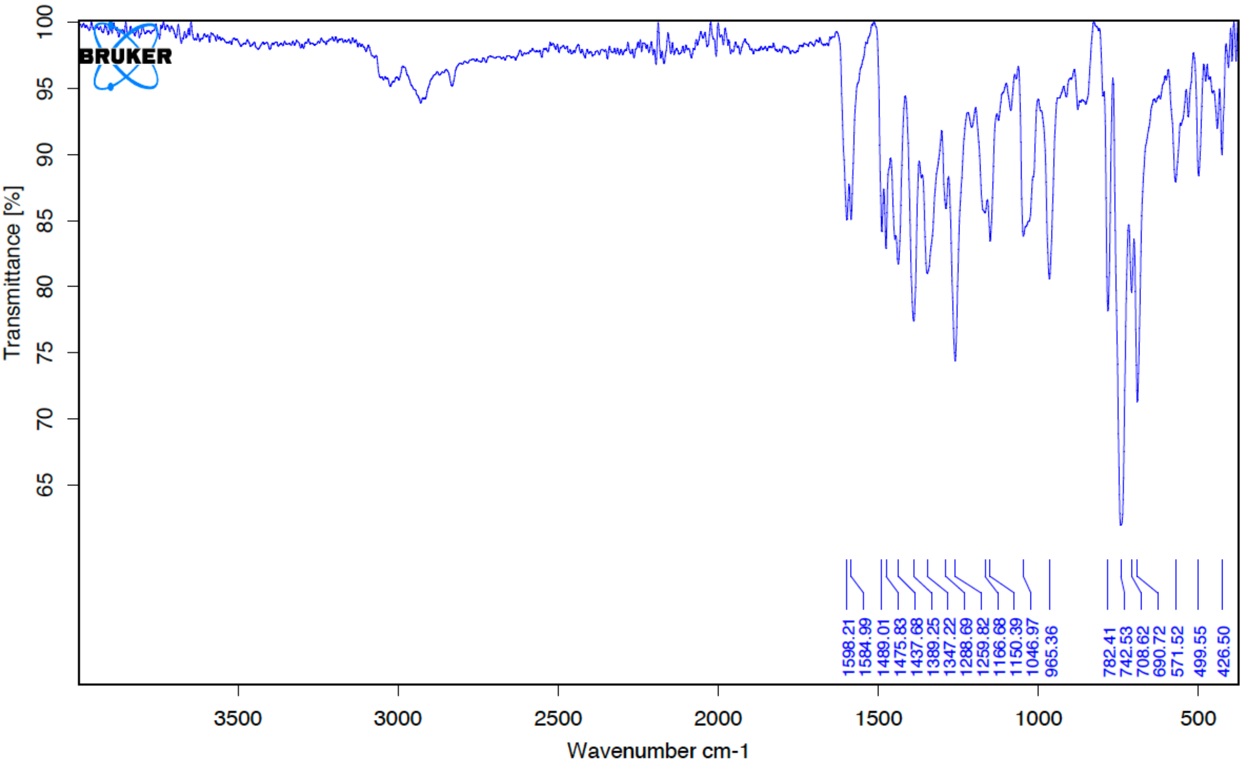


**Figure S35**. FT-IR spectrum

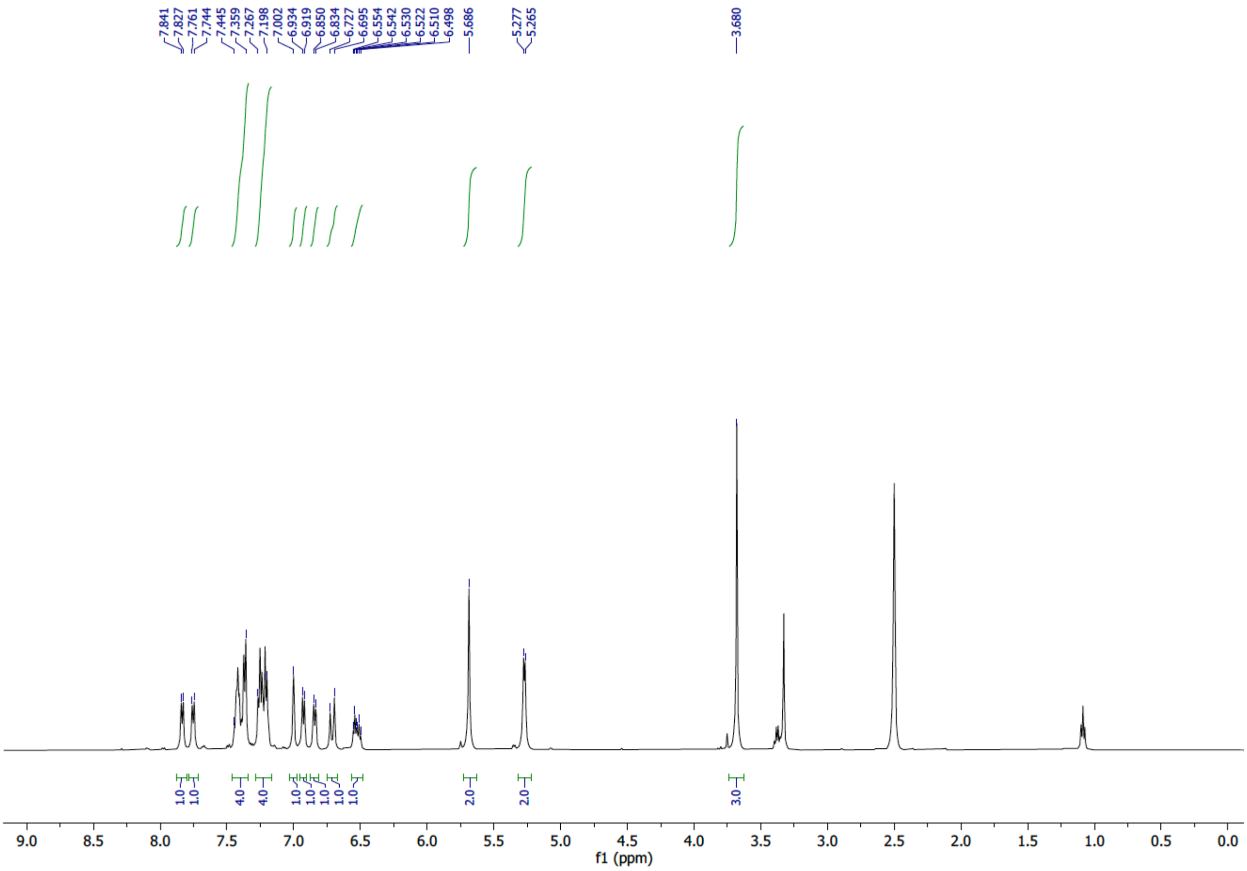


**Figure S36**. $^{1}H$ NMR spectrum ((DMSO-$d_6$)

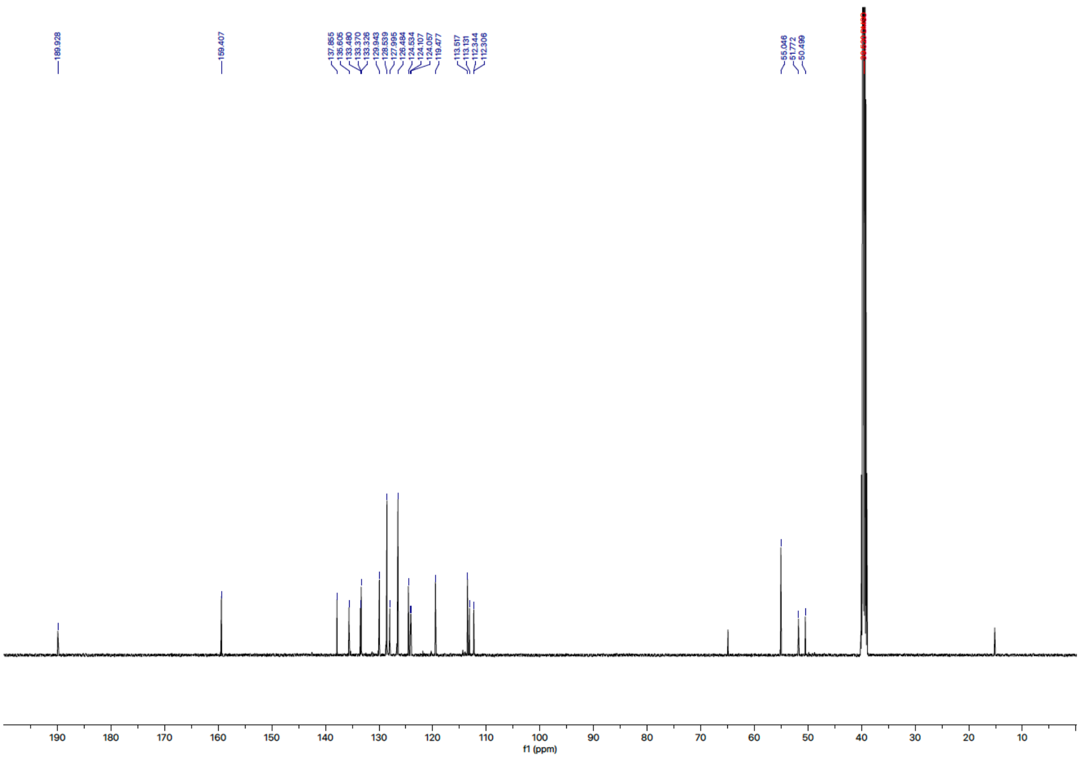


**Figure S37**. $^{13}C\{^{1}H\}$ NMR spectrum (DMSO-$d_6$)

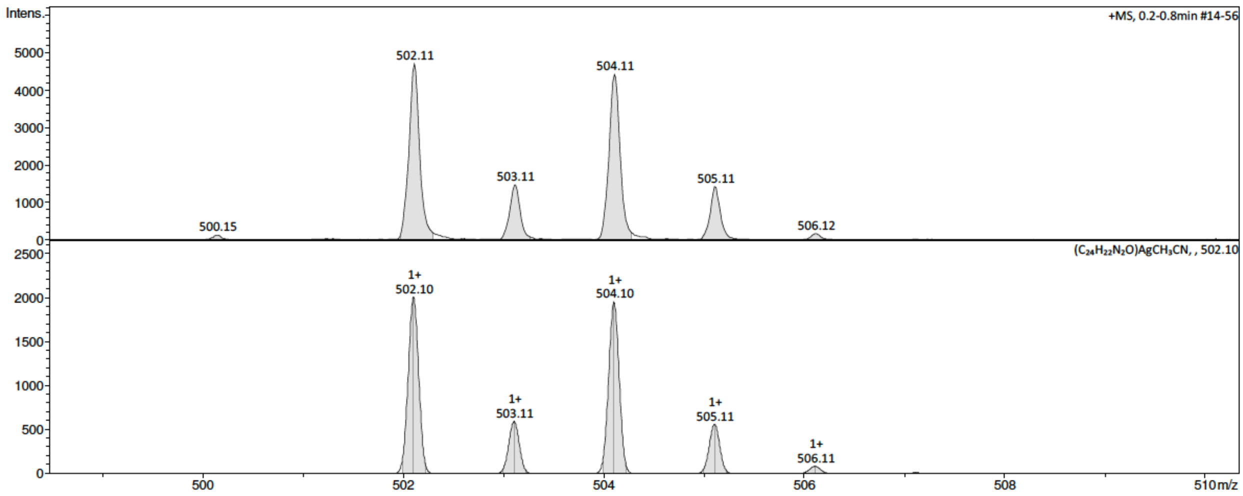


**Figure S38**. Mass spectrum (ESI-TOF): exp. spectrum (top); calc. spectrum (bottom) for $C_{26}H_{25}N_3OAg$ ([M - Br + $CH_3CN$]$^+$)

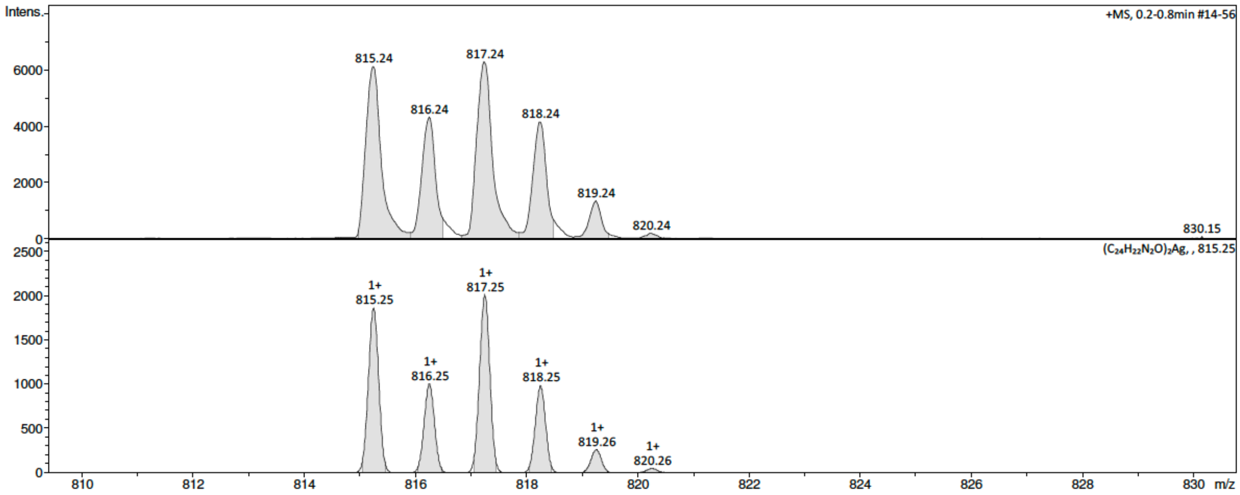


**Figure S39**. Mass spectrum (ESI-TOF): exp. spectrum (top); calc. spectrum (bottom) for $C_{48}H_{44}N_4O_2Ag$ ([M - Br + $C_{24}H_{22}N_2O$]$^+$)

**Table S9.** Optimized Cartesian Coordinates of **1c**

| Atom | Cartesian Coordinates | | |
|---|---|---|---|
| | x | y | z |
| Ag | -0,12097 | 1,077625 | -2,83267 |
| Br | -1,31364 | 0,811243 | -5,05849 |
| N | -0,27915 | 2,185708 | 0,102517 |
| N | 1,51676 | 0,958536 | -0,12272 |
| C | 0,364717 | 2,208551 | 1,350035 |
| C | 1,525412 | 1,41066 | 1,204714 |
| C | 0,431972 | 1,439921 | -0,79505 |
| C | -2,31979 | 2,443411 | -1,34864 |
| C | 0,060533 | 2,844375 | 2,566108 |

| | | | |
|---|---|---|---|
| C | 2,42214 | 1,213735 | 2,268058 |
| C | -1,4761 | 2,984124 | -0,20915 |
| C | 2,115489 | 1,847626 | 3,481195 |
| C | 0,956445 | 2,647049 | 3,627456 |
| C | -2,63356 | 3,27498 | -2,43419 |
| C | -2,85687 | 1,124983 | -1,28223 |
| C | -3,95783 | 1,448217 | -3,42535 |
| C | 2,54406 | 0,1251 | -0,7456 |
| C | -3,66621 | 0,640587 | -2,31692 |
| C | -3,47053 | 2,791397 | -3,47508 |
| H | 2,791818 | 1,719936 | 4,339604 |
| H | 0,753904 | 3,12734 | 4,595933 |
| C | 2,594047 | -1,30867 | -0,29694 |
| C | 1,69704 | -1,93215 | 0,511974 |
| C | 1,720128 | -3,33216 | 0,942199 |
| C | 0,742693 | -3,77378 | 1,874591 |
| H | -0,83305 | 3,4725 | 2,691207 |
| H | 3,319163 | 0,587918 | 2,159211 |
| H | -1,16548 | 4,027965 | -0,42394 |
| H | -2,09194 | 3,015058 | 0,714872 |
| H | -2,22909 | 4,295694 | -2,47028 |
| H | -2,63532 | 0,489572 | -0,41153 |
| H | -4,60058 | 1,100954 | -4,2468 |
| H | 3,534769 | 0,610577 | -0,59274 |
| H | 2,35405 | 0,176925 | -1,84502 |
| H | -4,06885 | -0,38149 | -2,27225 |
| O | -3,86761 | 3,510018 | -4,52953 |
| H | 3,453653 | -1,86653 | -0,70576 |
| H | 0,855215 | -1,33879 | 0,910866 |
| C | 2,676018 | -4,27603 | 0,473106 |
| C | 0,722848 | -5,09823 | 2,3271 |
| H | -0,00533 | -3,0561 | 2,247837 |
| H | 3,440547 | -3,97139 | -0,25779 |
| C | 2,653311 | -5,59873 | 0,923353 |
| C | 1,679027 | -6,01648 | 1,853669 |
| H | 3,397595 | -6,31726 | 0,548017 |
| H | 1,666175 | -7,05853 | 2,207372 |
| H | -0,03969 | -5,42046 | 3,052548 |
| C | -3,35329 | 4,828887 | -4,74373 |
| H | -2,2483 | 4,804663 | -4,85113 |
| H | -3,64344 | 5,511118 | -3,91716 |
| H | -3,80721 | 5,180412 | -5,68625 |

**Bromo[1-(3,5-dimethoxy-benzyl)-3-cinnamyl-benzimidazol-2-ylidene]silver(I) (1d)**

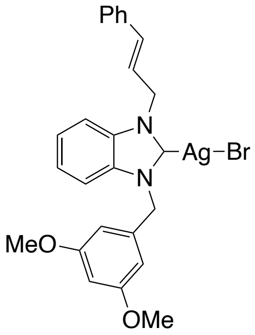


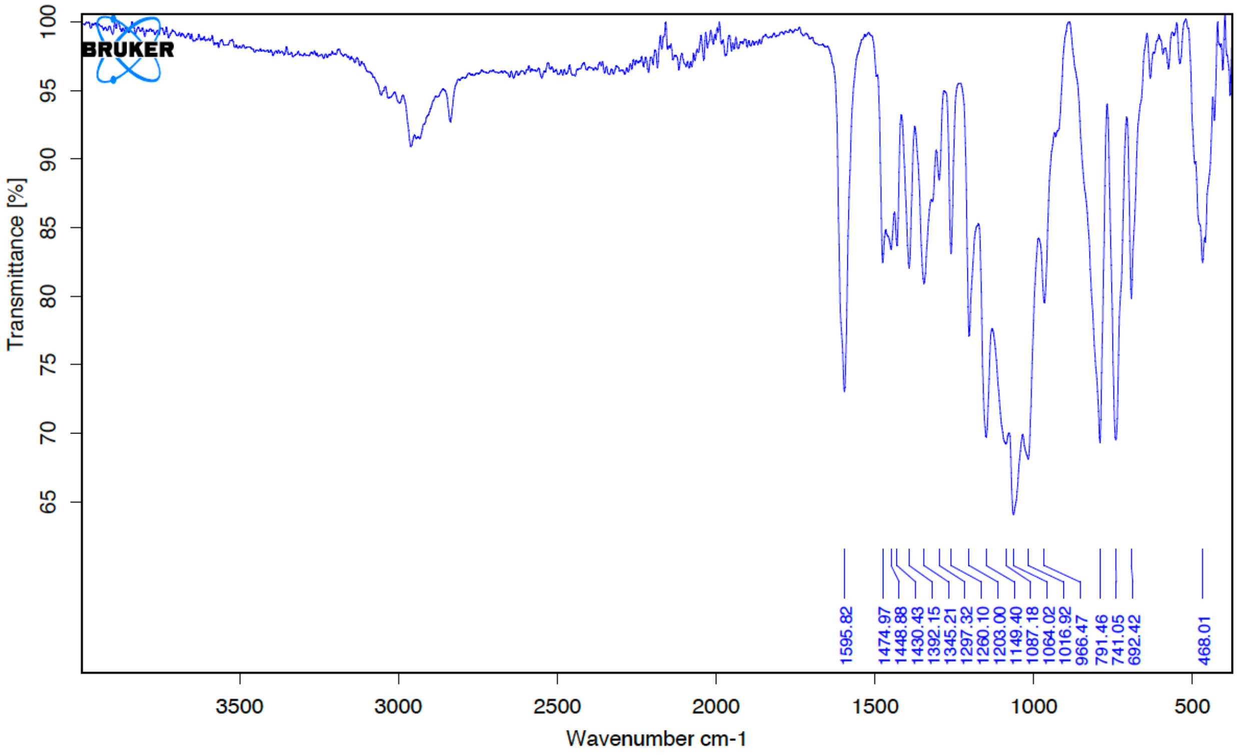


**Figure S40**. FT-IR spectrum

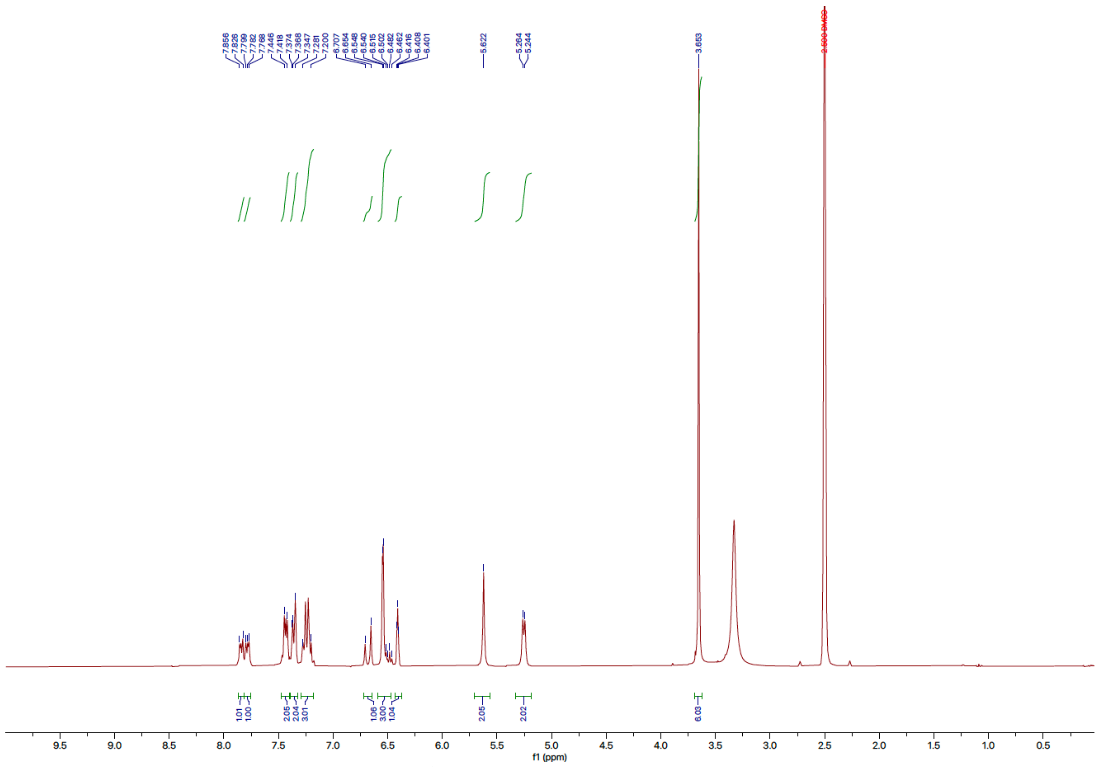


**Figure S41**. $^{1}$H NMR spectrum ((DMSO-$d_6$)

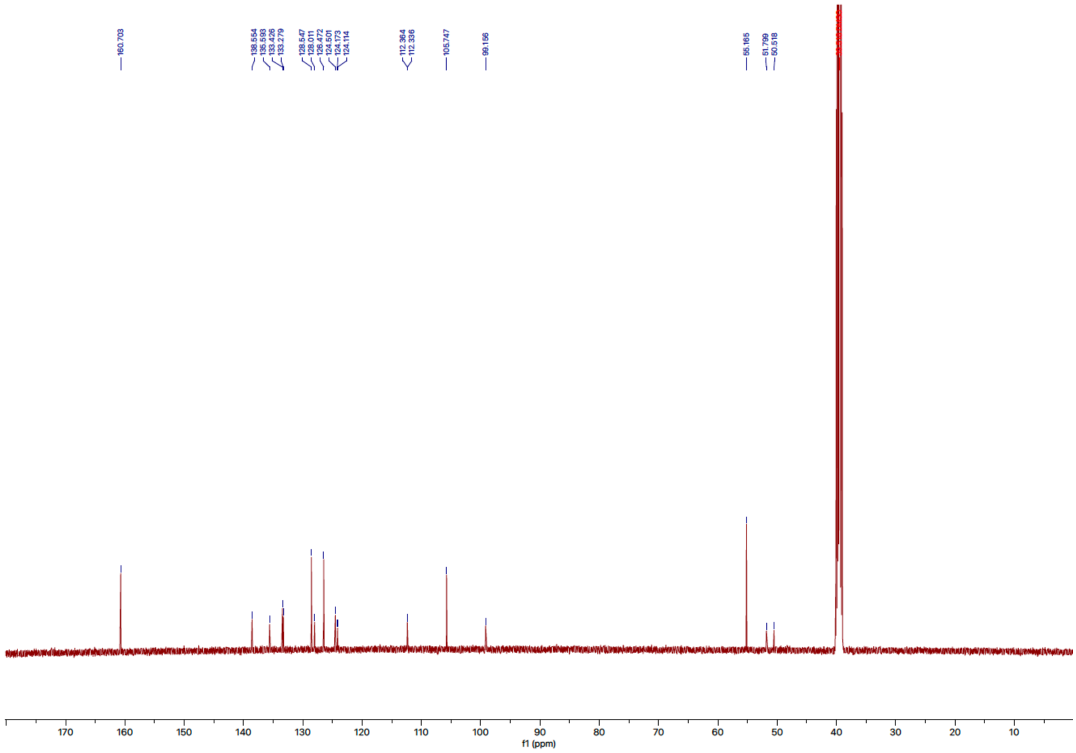


**Figure S42**. $^{13}$C{$^{1}$H} NMR spectrum (DMSO-$d_6$)

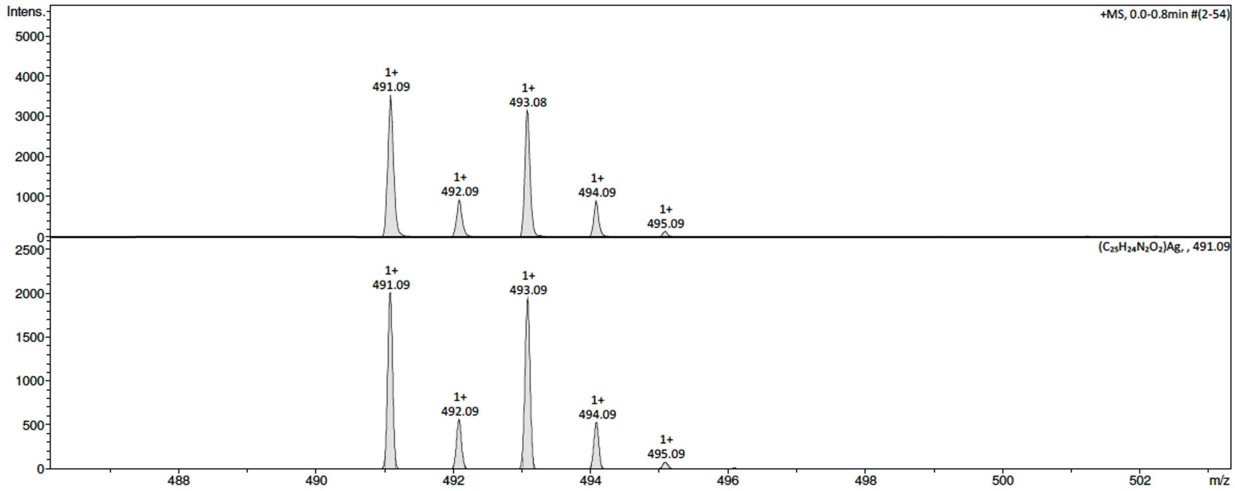


**Figure S43**. Mass spectrum (ESI-TOF): exp. spectrum (top); calc. spectrum (bottom) for $C_{25}H_{24}N_2O_2Ag$ ($[M - Br]^+$)

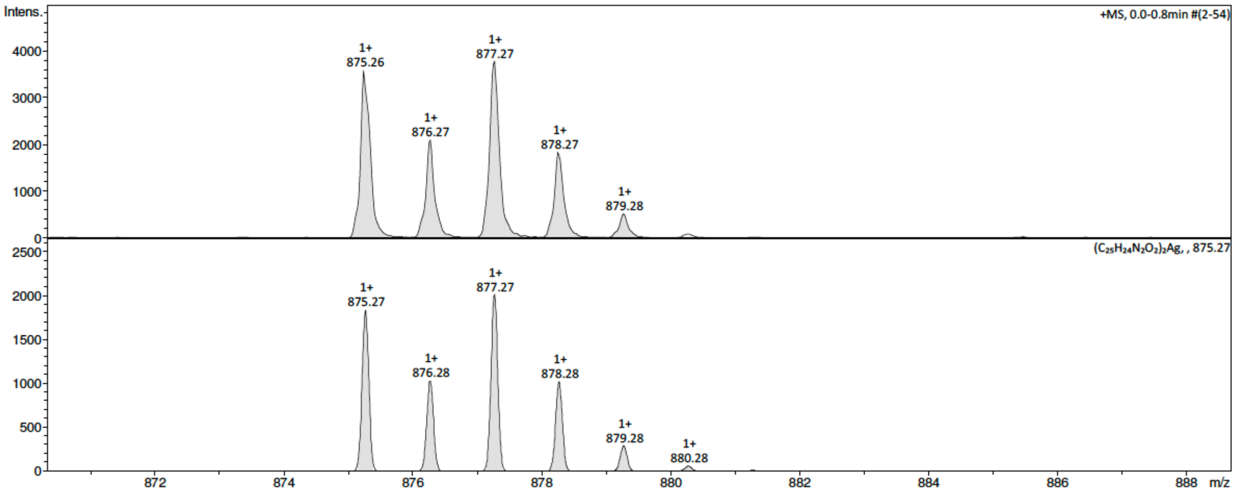


**Figure S44**. Mass spectrum (ESI-TOF): exp. spectrum (top); calc. spectrum (bottom) for $C_{50}H_{48}N_4O_4Ag$ ($[M - Br + C_{25}H_{24}N_2O_2]^+$)

**Table S10.** Optimized Cartesian Coordinates of **1d**

| Atom | Cartesian Coordinates | | |
|---|---|---|---|
| | x | y | z |
| Ag | -0,08586 | -0,56149 | 0,257901 |
| Br | -1,47215 | -1,95912 | -1,36277 |
| N | 0,113057 | 2,498822 | 1,360217 |
| N | 1,67144 | 1,15554 | 2,096146 |
| C | 0,931305 | 3,272667 | 2,197436 |
| C | 1,941121 | 2,401328 | 2,675761 |
| C | 0,557056 | 1,211909 | 1,311434 |
| C | -1,70014 | 2,126161 | -0,34253 |
| C | 0,881272 | 4,622917 | 2,58307 |

| | | | |
|---|---|---|---|
| C | 2,938881 | 2,850155 | 3,557906 |
| C | -1,10738 | 3,025532 | 0,724712 |
| C | 2,890182 | 4,199639 | 3,937759 |
| C | 1,880869 | 5,069669 | 3,460169 |
| C | -1,05318 | 2,023362 | -1,60839 |
| C | -2,90639 | 1,465326 | -0,08899 |
| C | -2,83882 | 0,520102 | -2,35418 |
| C | 2,402134 | -0,09254 | 2,364028 |
| C | -3,48899 | 0,657966 | -1,09672 |
| C | -1,63426 | 1,238498 | -2,61444 |
| C | 2,333196 | -1,08387 | 1,230356 |
| C | 1,994076 | -2,39954 | 1,426938 |
| C | 2,125469 | -3,51724 | 0,492692 |
| C | 1,642064 | -4,79036 | 0,89263 |
| O | -4,64468 | 0,056876 | -0,78318 |
| H | -6,1617 | -1,23061 | -1,20166 |
| O | -1,14641 | 1,078064 | -3,86127 |
| H | 0,245855 | 1,483508 | -5,28426 |
| C | 2,725566 | -3,39366 | -0,78884 |
| C | 1,742498 | -5,8983 | 0,041427 |
| C | 2,823616 | -4,49996 | -1,63756 |
| C | 2,331603 | -5,75576 | -1,22684 |
| C | 0,04976 | 1,759151 | -4,23293 |
| C | -5,2554 | -0,85261 | -1,70561 |
| H | 0,095808 | 5,303536 | 2,225151 |
| H | 3,721519 | 2,182054 | 3,944055 |
| H | -0,84605 | 4,008595 | 0,279246 |
| H | -1,85629 | 3,215269 | 1,520902 |
| H | 3,653207 | 4,589707 | 4,627927 |
| H | 1,876817 | 6,119266 | 3,789101 |
| H | -0,12414 | 2,580477 | -1,786 |
| H | -3,42507 | 1,552577 | 0,875985 |
| H | -3,27456 | -0,05444 | -3,18116 |
| H | 2,018527 | -0,54428 | 3,30399 |
| H | 3,463374 | 0,184336 | 2,545052 |
| H | 2,789327 | -0,76313 | 0,276626 |
| H | 1,593535 | -2,67093 | 2,421728 |
| H | 1,179692 | -4,902 | 1,885995 |
| H | 3,125951 | -2,42383 | -1,12102 |
| H | 1,361059 | -6,87749 | 0,366918 |
| H | 3,292072 | -4,39105 | -2,62713 |
| H | 2,413574 | -6,62462 | -1,89767 |
| H | 0,90876 | 1,438597 | -3,60446 |
| H | -0,0728 | 2,861721 | -4,15835 |
| H | -4,56978 | -1,69677 | -1,92973 |
| H | -5,546 | -0,33743 | -2,64508 |

**Bromo[1-(naphthalen-1-ylmethyl)-3-cinnamyl-benzimidazol-2 ylidene]silver(I) (1e)**

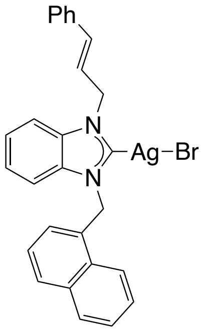


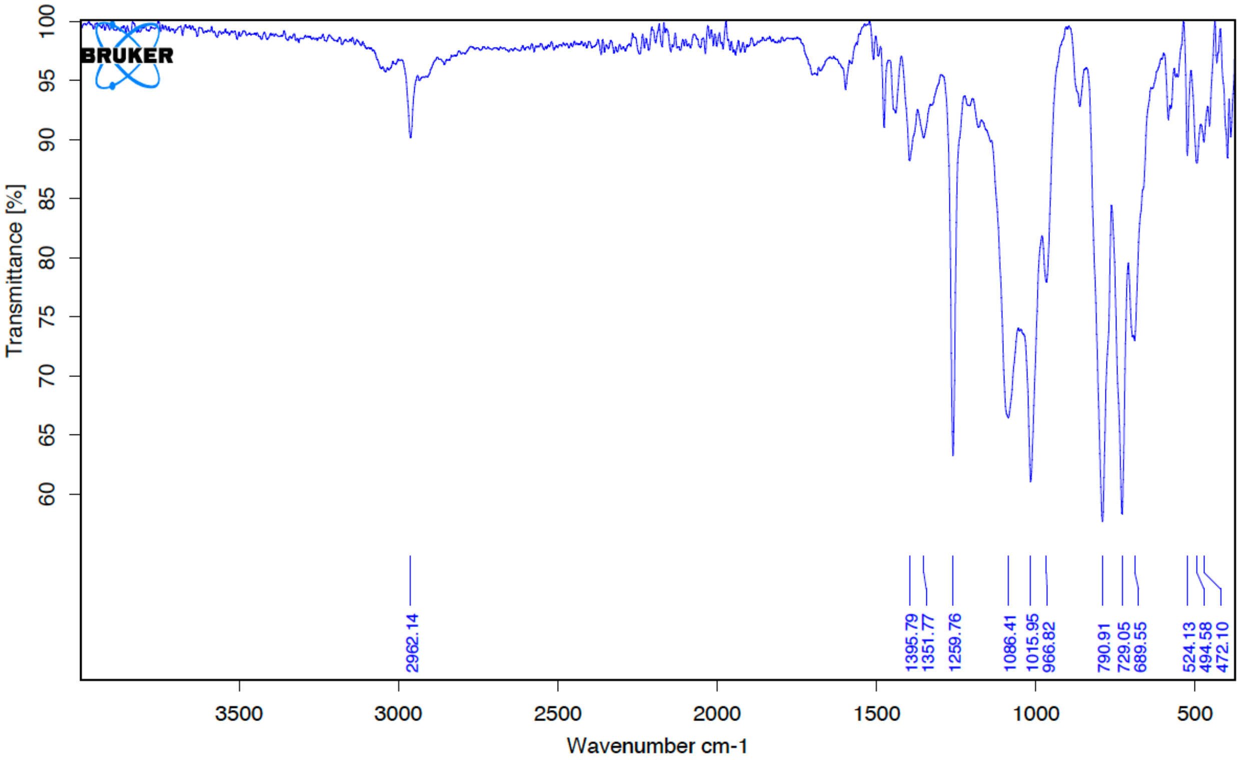


**Figure S45**. FT-IR spectrum

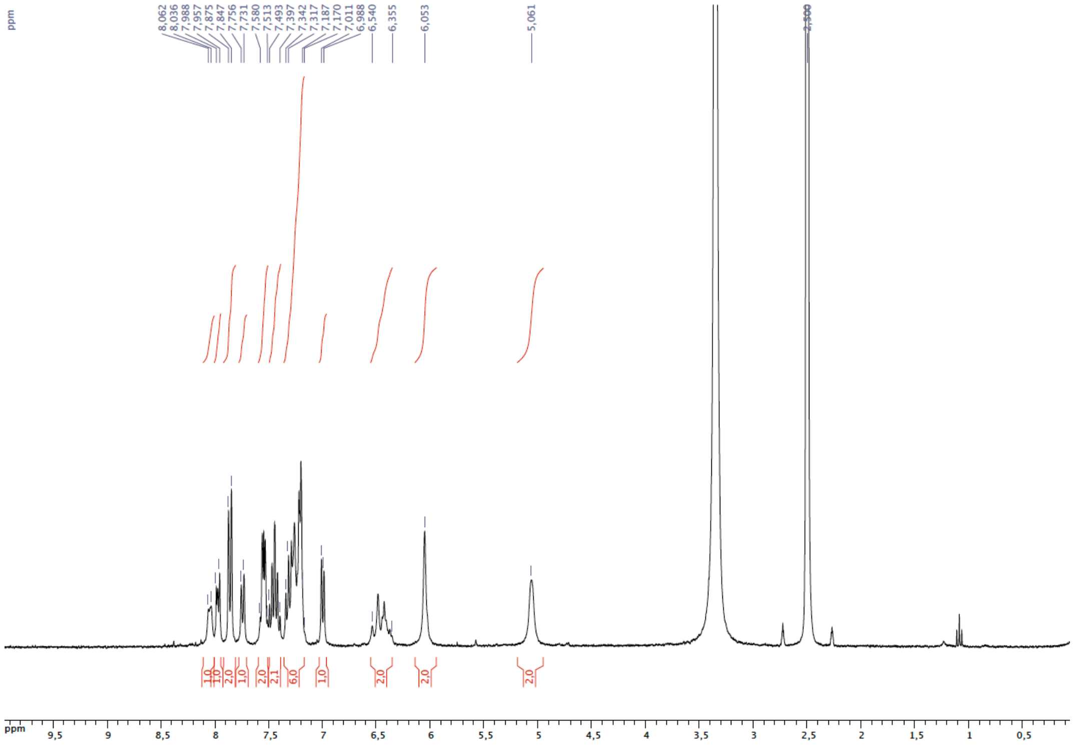

**Figure S46**. $^{1}$H NMR spectrum ((DMSO-$d_6$)

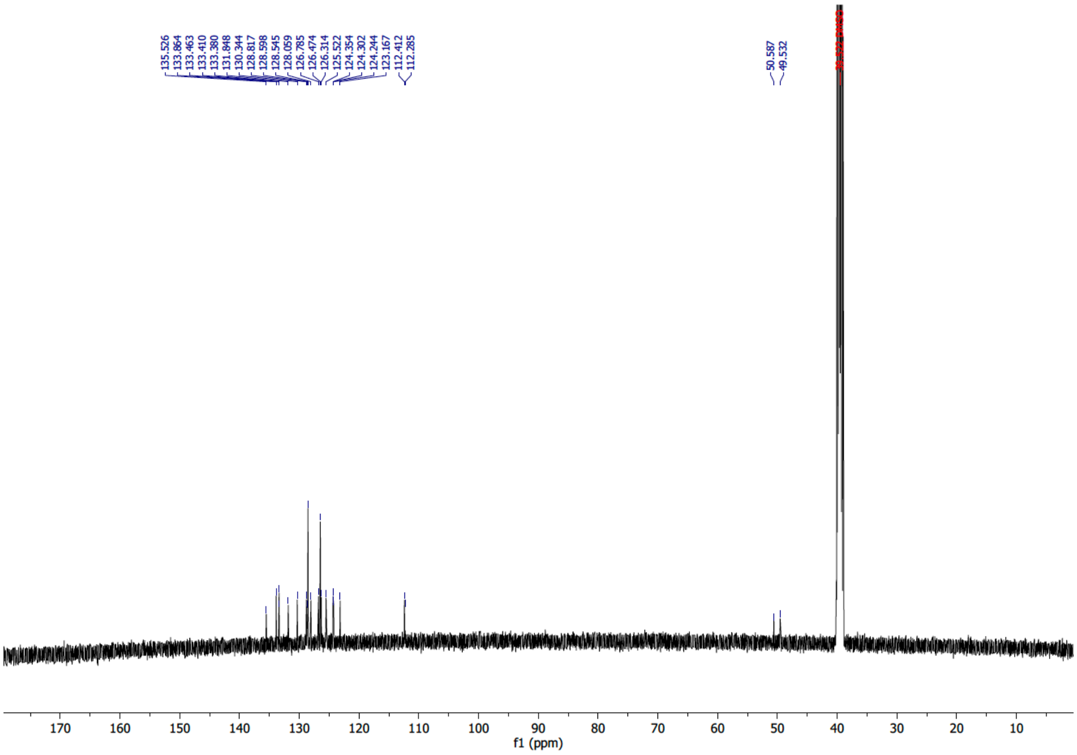

**Figure S47**. $^{13}$C{$^{1}$H} NMR spectrum (DMSO-$d_6$)

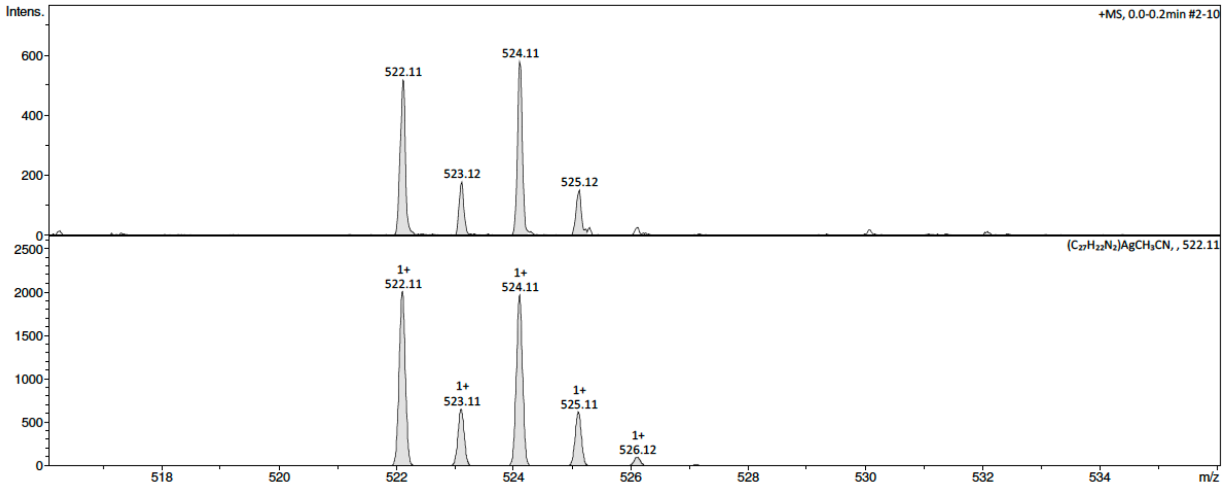


**Figure S48**. Mass spectrum (ESI-TOF): exp. spectrum (top); calc. spectrum (bottom) for $C_{29}H_{25}N_3Ag$ ([M - Br + $CH_3CN$]$^+$)

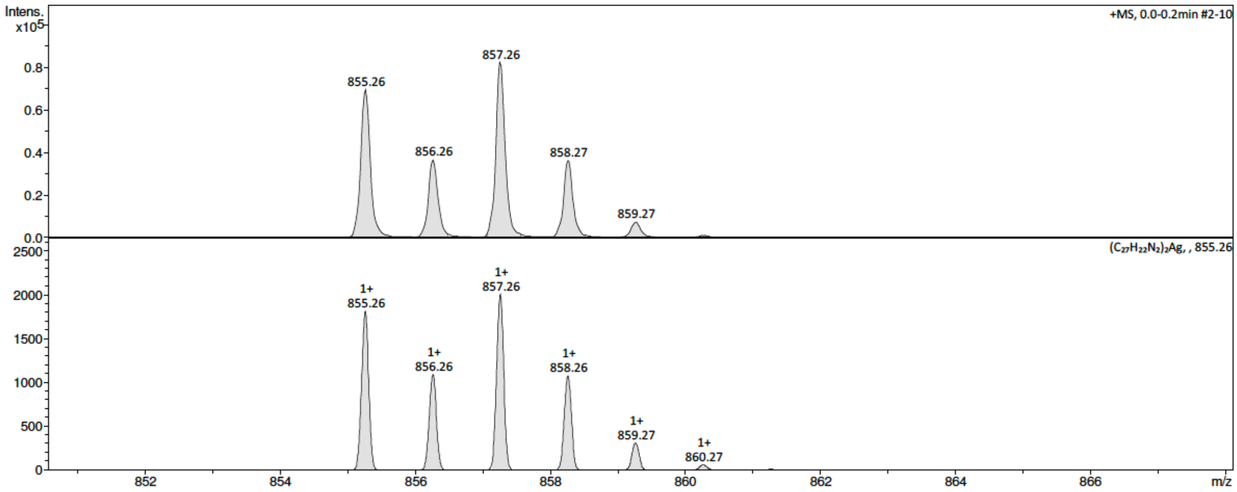


**Figure S49**. Mass spectrum (ESI-TOF): exp. spectrum (top); calc. spectrum (bottom) for $C_{54}H_{44}N_4Ag$ ([M - Br + $C_{27}H_{22}N_2$]$^+$)

**Table S11.** Optimized Cartesian Coordinates of **1e**

| Atom | Cartesian Coordinates | | |
|---|---|---|---|
| | x | y | z |
| Ag | -0,77924 | 0,75509 | -3,25019 |
| Br | -1,98454 | 0,422373 | -5,4685 |
| N | -0,74024 | 1,979758 | -0,33686 |
| N | 1,04614 | 0,765229 | -0,67277 |
| C | 0,012337 | 2,061816 | 0,845173 |
| C | 1,167891 | 1,272916 | 0,628124 |
| C | -0,10423 | 1,20427 | -1,26371 |
| C | -2,83181 | 2,304546 | -1,7184 |
| C | -0,19272 | 2,746287 | 2,055431 |
| C | 2,159375 | 1,134048 | 1,613912 |

| | | | |
|---|---|---|---|
| C | -1,97184 | 2,762578 | -0,55628 |
| C | 1,952566 | 1,816667 | 2,821665 |
| C | 0,798347 | 2,607056 | 3,038831 |
| C | -2,9246 | 3,123208 | -2,85409 |
| C | -3,61587 | 1,092578 | -1,642 |
| C | -4,44934 | 1,558277 | -3,93269 |
| C | 2,030344 | -0,07605 | -1,35159 |
| C | -4,43657 | 0,724579 | -2,77319 |
| C | -3,72315 | 2,758531 | -3,95914 |
| H | 2,706592 | 1,7365 | 3,619187 |
| H | 0,676989 | 3,127317 | 4,000339 |
| C | 2,152057 | -1,48751 | -0,84933 |
| C | 1,340517 | -2,09006 | 0,059772 |
| C | 1,434969 | -3,46662 | 0,550486 |
| C | 0,582953 | -3,87038 | 1,613775 |
| H | -1,081 | 3,369532 | 2,231886 |
| H | 3,054388 | 0,517291 | 1,450911 |
| H | -1,68339 | 3,823108 | -0,71002 |
| H | -2,55415 | 2,723977 | 0,387674 |
| H | -2,35066 | 4,06168 | -2,88946 |
| C | -3,63377 | 0,250253 | -0,4937 |
| H | -5,08146 | 1,275524 | -4,78766 |
| H | 3,020313 | 0,432279 | -1,31087 |
| H | 1,74103 | -0,07646 | -2,43037 |
| C | -5,22206 | -0,46149 | -2,71143 |
| H | -3,76694 | 3,408845 | -4,84477 |
| H | 2,988111 | -2,04642 | -1,3031 |
| H | 0,522427 | -1,4938 | 0,501264 |
| C | 2,342509 | -4,42378 | 0,016799 |
| C | 0,640235 | -5,16935 | 2,131885 |
| H | -0,12506 | -3,14125 | 2,03965 |
| H | 3,006019 | -4,15126 | -0,81801 |
| C | 2,395113 | -5,7219 | 0,531593 |
| C | 1,547408 | -6,10097 | 1,592869 |
| H | 3,099044 | -6,45188 | 0,104105 |
| H | 1,594455 | -7,12342 | 1,997219 |
| H | -0,02189 | -5,46064 | 2,961378 |
| H | -3,02254 | 0,505784 | 0,384202 |
| C | -4,42213 | -0,90171 | -0,45804 |
| H | -5,83704 | -0,7368 | -3,58225 |
| C | -5,21775 | -1,26393 | -1,57109 |
| H | -4,4264 | -1,53137 | 0,444691 |
| H | -5,83184 | -2,17602 | -1,53472 |

**Bromo[1-(pyren-1-ylmethyl)-3-cinnamyl-benzimidazol-2-yliden]silver(I) (1f)**

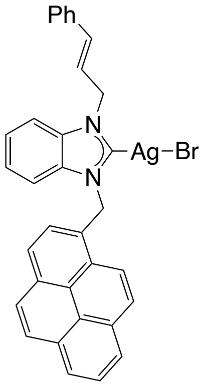


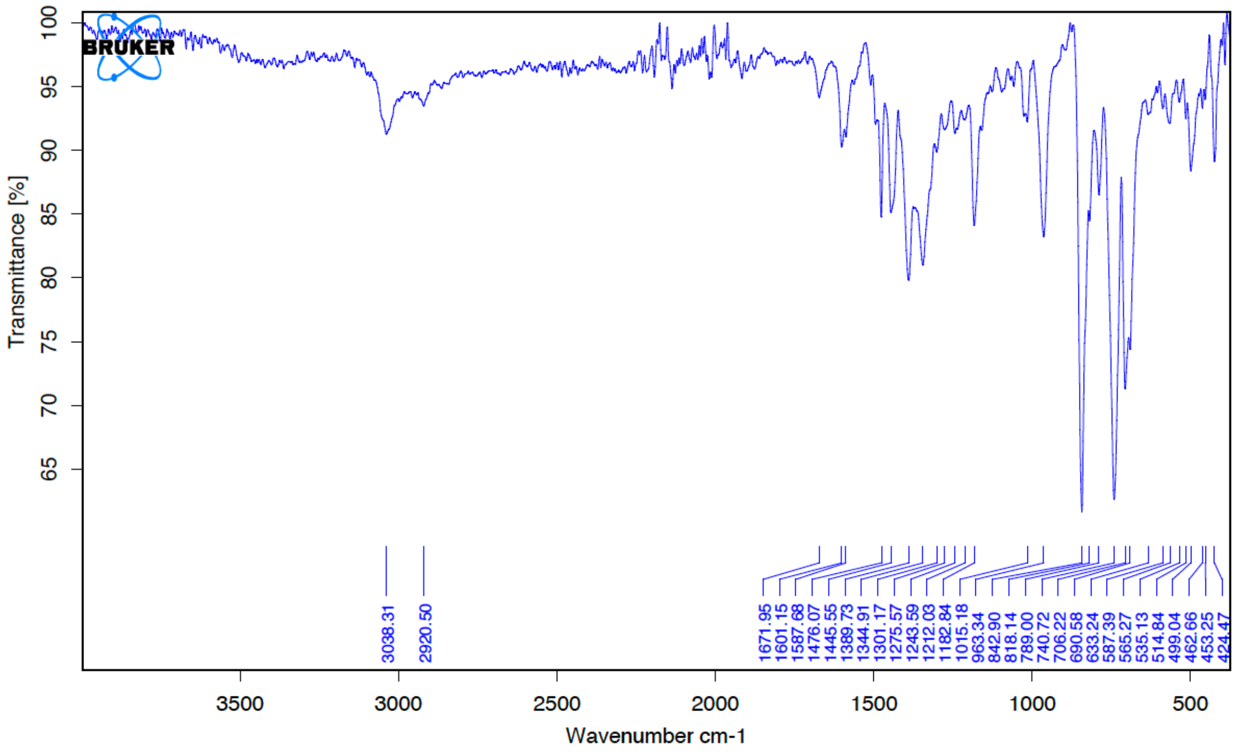


**Figure S50**. FT-IR spectrum

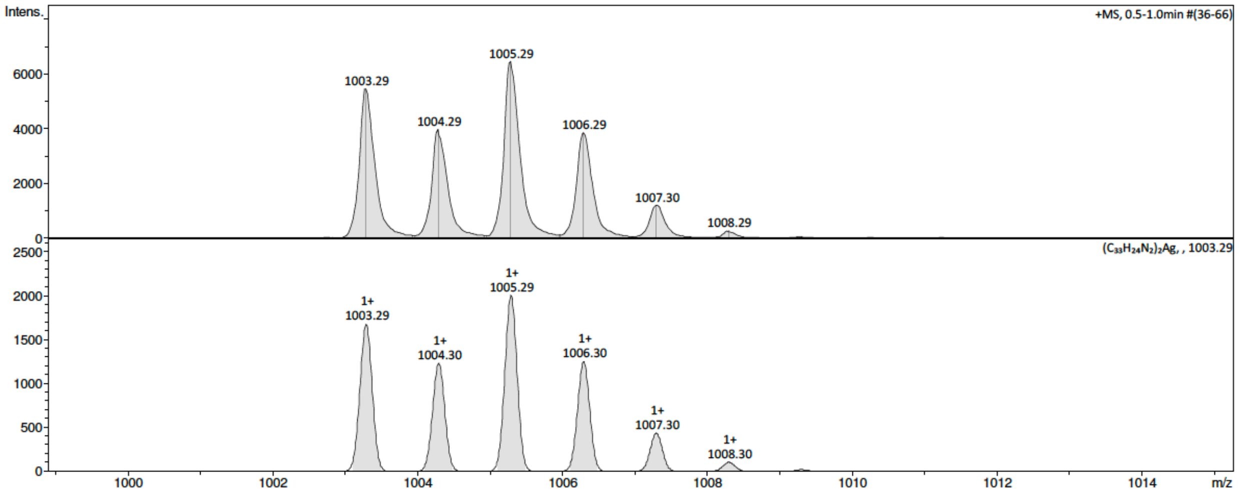


**Figure S51**. Mass spectrum (ESI-TOF): exp. spectrum (top); calc. spectrum (bottom) for $C_{66}H_{48}N_4Ag$ ([M - Br + $C_{33}H_{24}N_2$]$^+$)

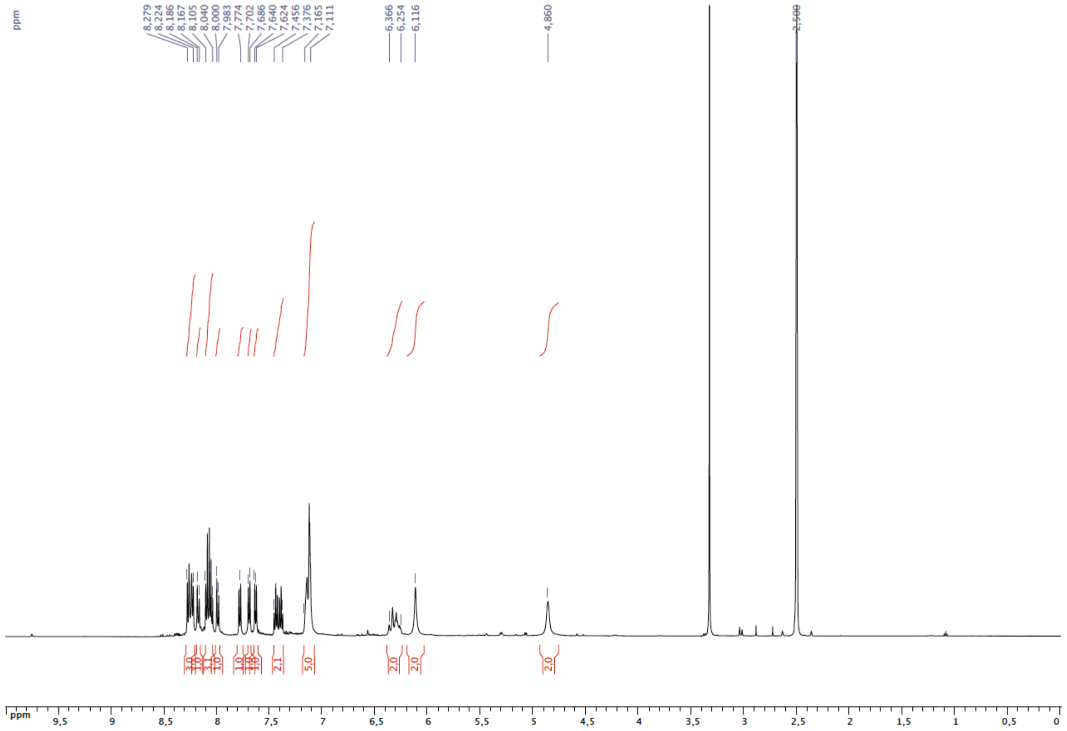


**Figure S52**. $^{1}H$ NMR spectrum ((DMSO-$d_6$)

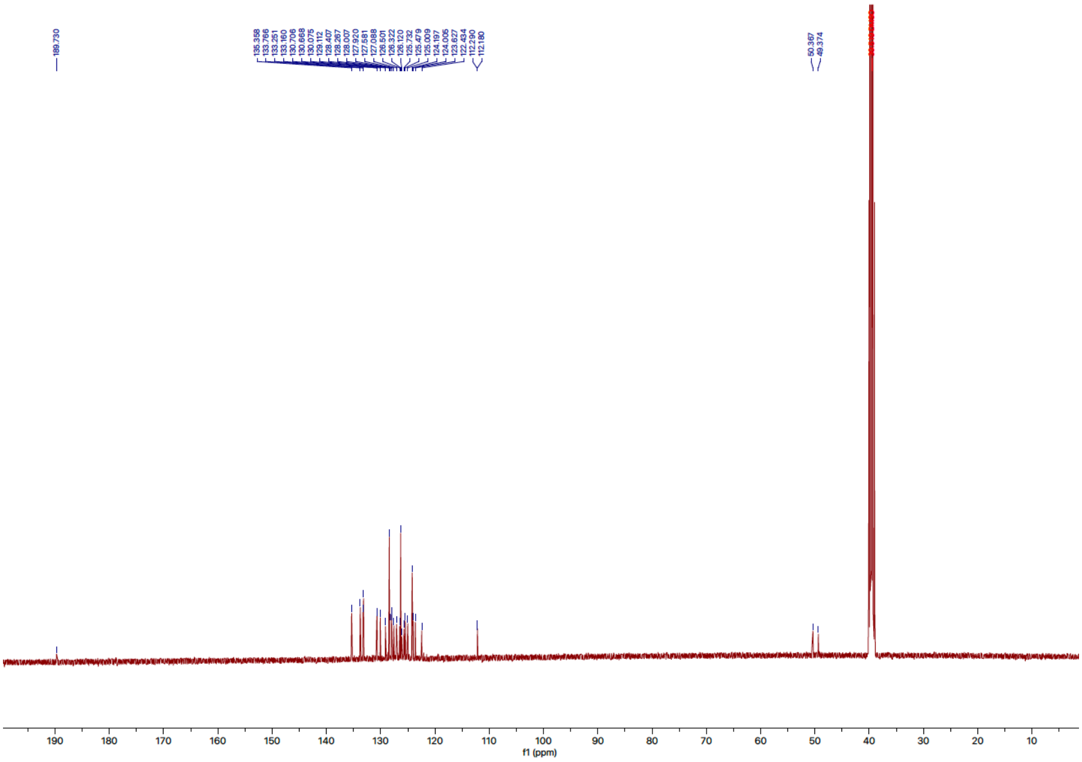


**Figure S53**. $^{13}C\{^{1}H\}$ NMR spectrum (DMSO-$d_6$)

**Table S12.** Optimized Cartesian Coordinates of **1f**

| Atom | Cartesian Coordinates | | |
|---|---|---|---|
| | x | y | z |
| Ag | -1,24754 | -0,42767 | -2,92709 |
| Br | -2,79259 | -1,0125 | -4,8509 |
| N | -0,9343 | 1,216369 | -0,27289 |
| N | 0,945164 | 0,192169 | -0,71403 |
| C | -0,04777 | 1,578853 | 0,753258 |
| C | 1,171644 | 0,919852 | 0,462101 |
| C | -0,33185 | 0,367289 | -1,16088 |
| C | -2,90044 | 1,855671 | -1,72291 |
| C | -0,18178 | 2,403652 | 1,883757 |
| C | 2,303114 | 1,06811 | 1,281355 |
| C | -2,34708 | 1,636241 | -0,32723 |
| C | 2,170049 | 1,899714 | 2,402673 |
| C | 0,949917 | 2,553396 | 2,699594 |
| C | -2,42559 | 2,92688 | -2,56275 |
| C | -3,94187 | 1,034103 | -2,17494 |
| C | -4,08319 | 2,256372 | -4,30499 |
| C | 1,944963 | -0,62452 | -1,40223 |
| C | -4,51269 | 1,200542 | -3,45124 |
| C | -3,02895 | 3,122986 | -3,85497 |
| H | 3,031971 | 2,041613 | 3,071642 |
| H | 0,884266 | 3,18816 | 3,595765 |
| C | 2,325523 | -1,90655 | -0,71089 |
| C | 1,686091 | -2,4632 | 0,348951 |
| C | 2,022788 | -3,71679 | 1,032324 |
| C | 1,23299 | -4,12004 | 2,140311 |
| H | -1,12695 | 2,906113 | 2,133907 |
| H | 3,245579 | 0,547546 | 1,062943 |
| H | -2,4226 | 2,573388 | 0,260555 |
| H | -2,96507 | 0,878923 | 0,198208 |
| C | -1,38841 | 3,826766 | -2,15539 |
| H | -4,31296 | 0,223596 | -1,52846 |
| C | -4,67924 | 2,479538 | -5,58587 |
| H | 2,849171 | 0,001085 | -1,57517 |
| H | 1,526226 | -0,83517 | -2,41382 |
| H | -5,33885 | 0,548835 | -3,7696 |
| C | -2,5928 | 4,193453 | -4,69754 |
| H | 3,197949 | -2,40902 | -1,16223 |
| H | 0,81309 | -1,93133 | 0,766913 |
| C | 3,104513 | -4,55094 | 0,642345 |
| C | 1,509211 | -5,30579 | 2,832839 |
| H | 0,389303 | -3,48543 | 2,457012 |
| H | 3,737672 | -4,2715 | -0,21341 |
| C | 3,37927 | -5,73497 | 1,333298 |
| C | 2,584121 | -6,11863 | 2,431706 |
| H | 4,220911 | -6,36993 | 1,016987 |

| | | | |
|---|---|---|---|
| H | 2,803449 | -7,05168 | 2,972402 |
| H | 0,884761 | -5,59857 | 3,690506 |
| C | -0,9649 | 4,860952 | -2,97461 |
| C | -1,54781 | 5,075843 | -4,26317 |
| C | -3,20409 | 4,396887 | -5,98222 |
| H | -0,1665 | 5,538461 | -2,6332 |
| C | -4,25455 | 3,515815 | -6,39861 |
| H | -5,48148 | 1,804882 | -5,9214 |
| C | -2,75194 | 5,46755 | -6,80065 |
| H | -0,90946 | 3,70225 | -1,17427 |
| H | -4,7225 | 3,673259 | -7,3823 |
| C | -1,72587 | 6,322158 | -6,37158 |
| C | -1,12895 | 6,132701 | -5,11775 |
| H | -0,32585 | 6,807175 | -4,7817 |
| H | -3,22036 | 5,620449 | -7,78518 |
| H | -1,391 | 7,144739 | -7,02079 |

## **Interaction detail and interaction residue of tested compounds against *Acanthamoeba castellanii* CYP51**

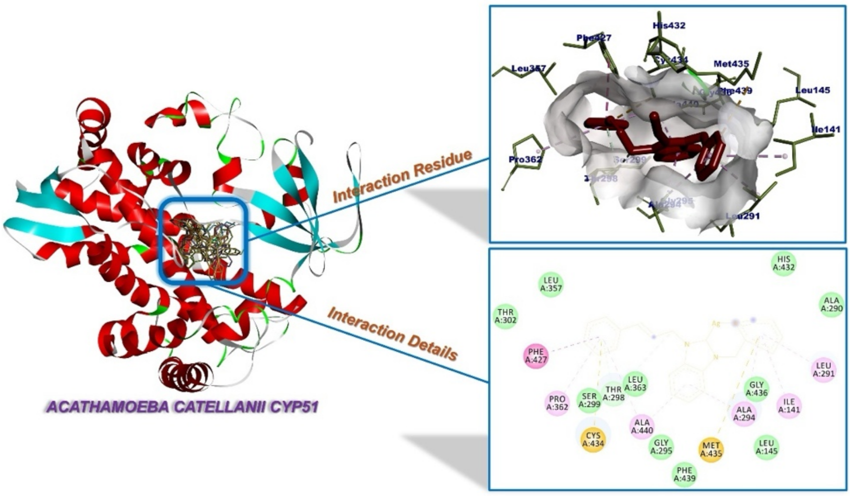


**Figure S54.** Interaction detail and interaction residue of **1a** against *Acanthamoeba castellanii* CYP51

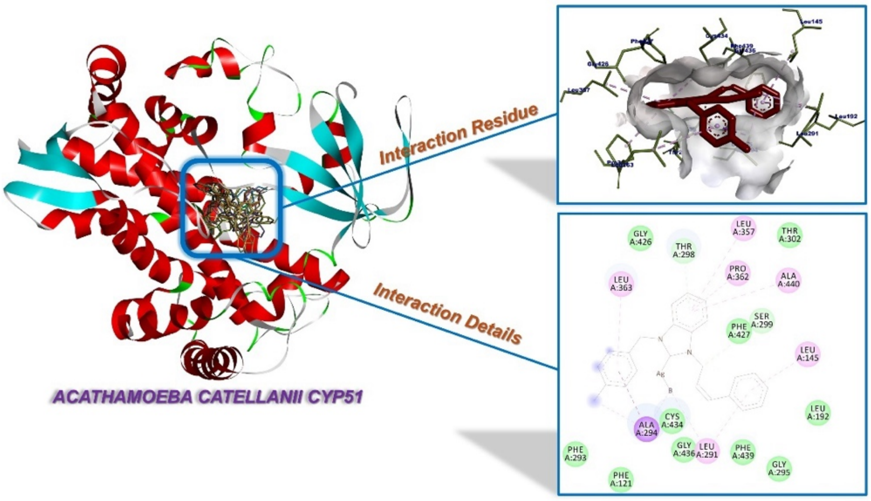


**Figure S55.** Interaction detail and interaction residue of **1b** against *Acanthamoeba castellanii* CYP51

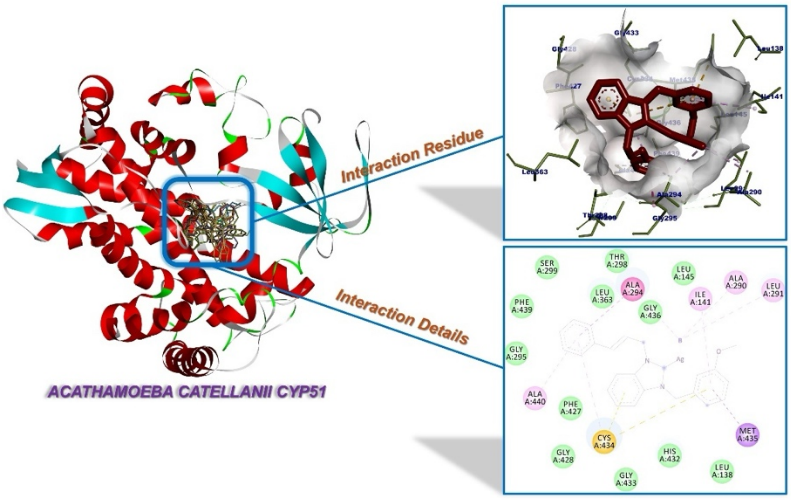


**Figure S56.** Interaction detail and interaction residue of **1c** against Acanthamoeba castellanii CYP51

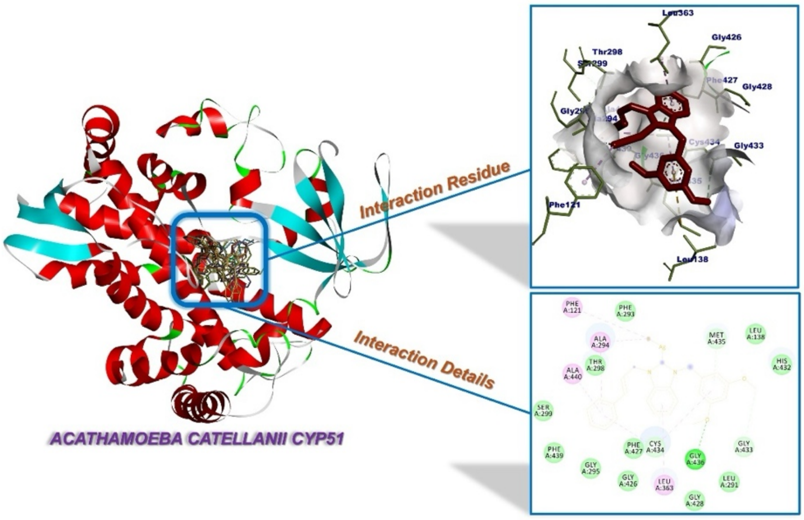


**Figure S57.** Interaction detail and interaction residue of **1d** against *Acanthamoeba castellanii* CYP51

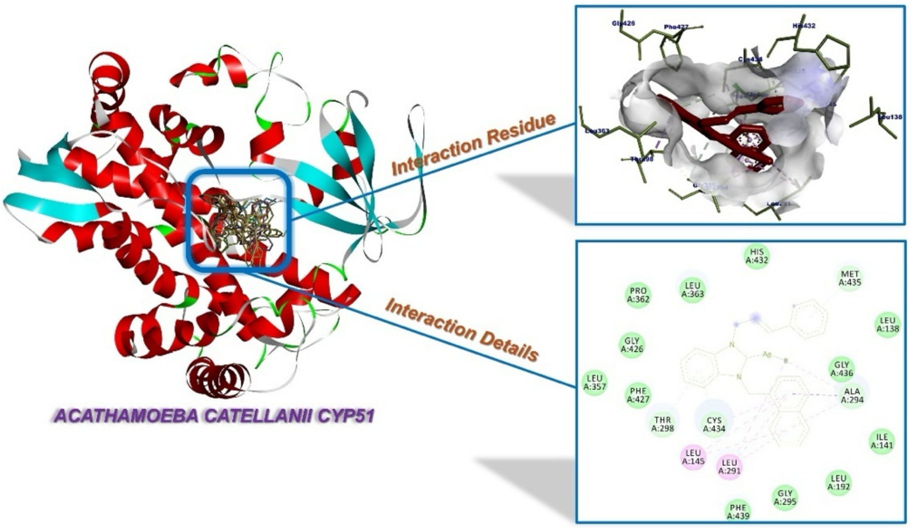


**Figure S58.** Interaction detail and interaction residue of **1e** against *Acanthamoeba castellanii* CYP51

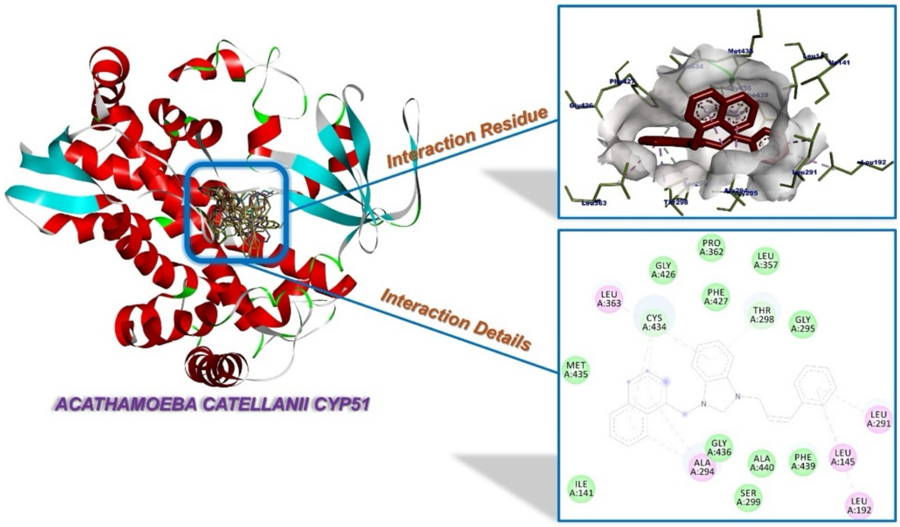


**Figure S59.** Interaction detail and interaction residue of **1f** against *Acanthamoeba castellanii* CYP51

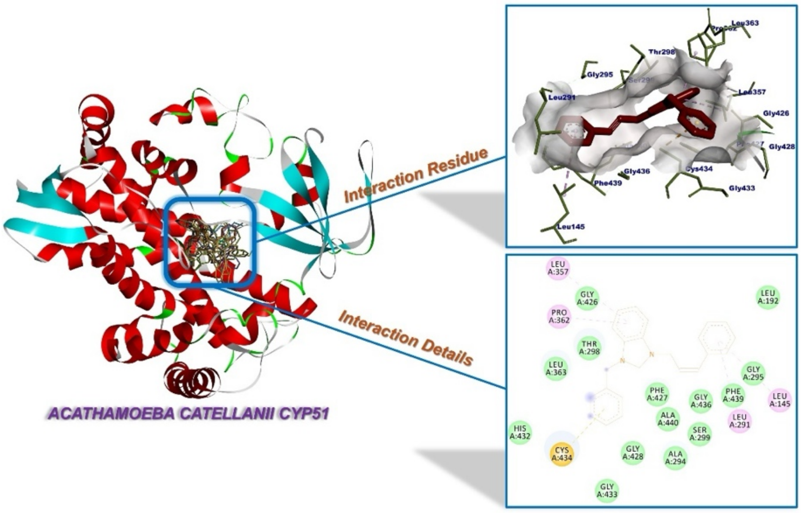


**Figure S60.** Interaction detail and interaction residue of **3a** against *Acanthamoeba castellanii* CYP51

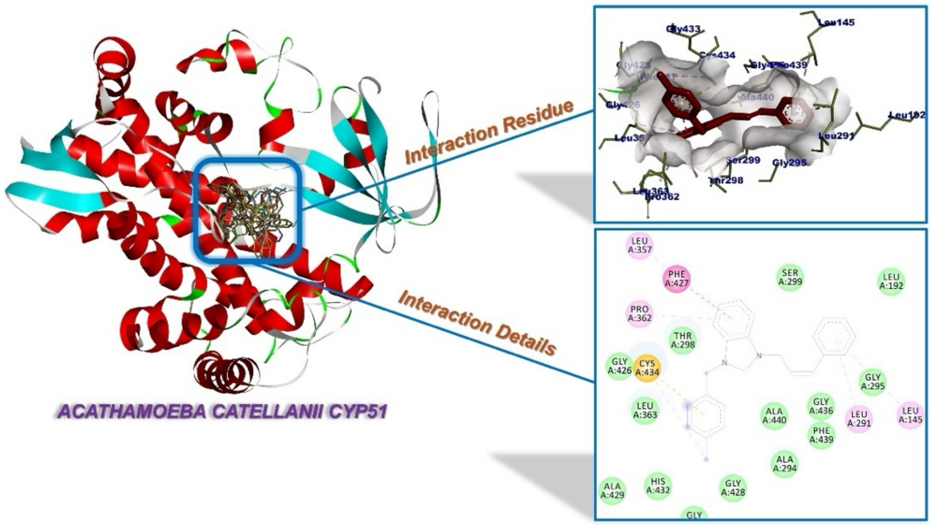


**Figure S61.** Interaction detail and interaction residue of **3b** against *Acanthamoeba castellanii* CYP51

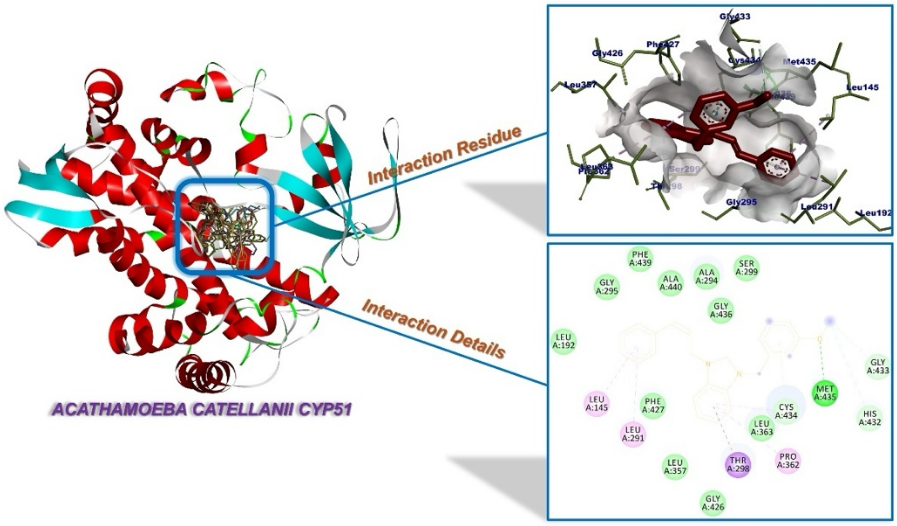


**Figure S62.** Interaction detail and interaction residue of **3c** against *Acanthamoeba castellanii* CYP51

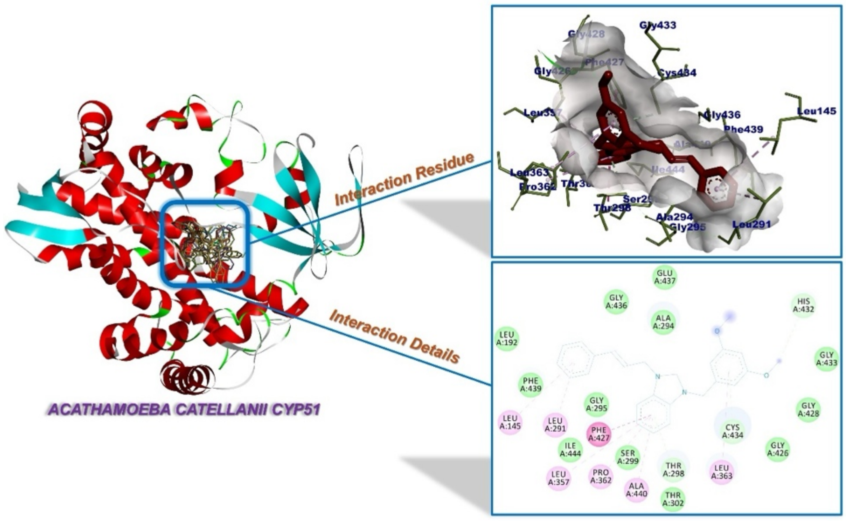


**Figure S63.** Interaction detail and interaction residue of **3d** against *Acanthamoeba castellanii* CYP51

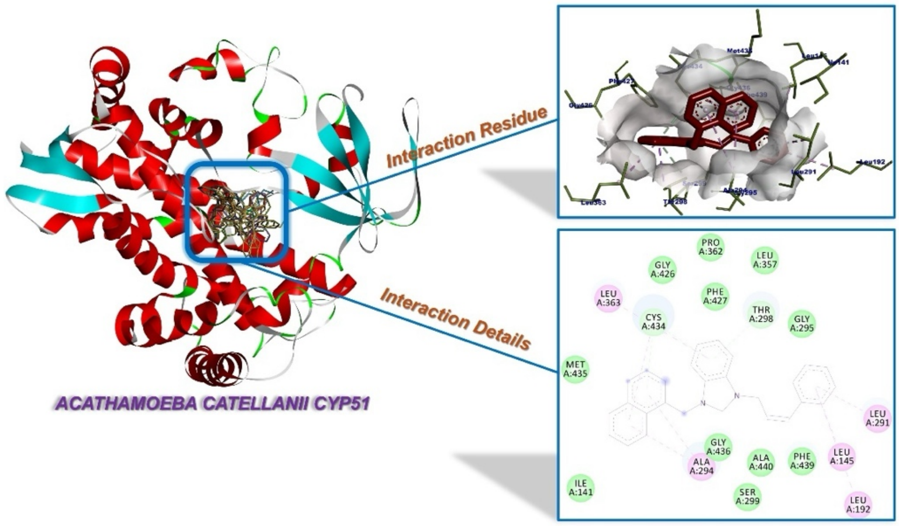


**Figure S64.** Interaction detail and interaction residue of **3e** against *Acanthamoeba castellanii* CYP51

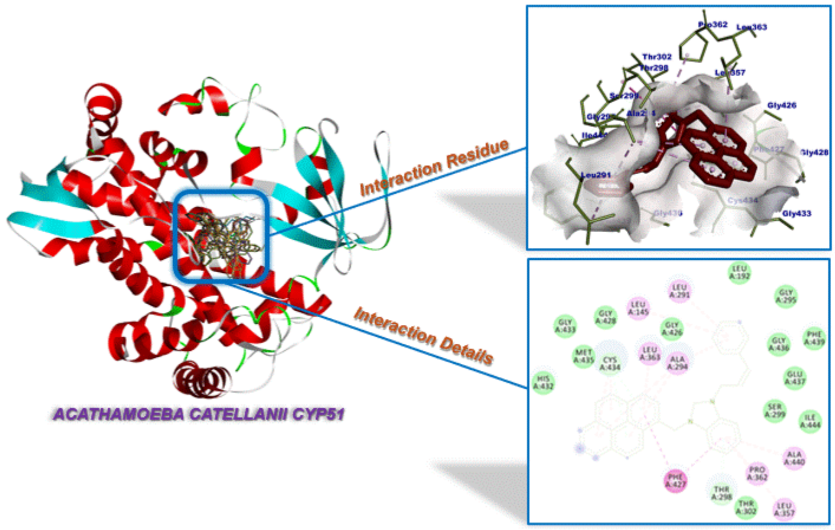


**Figure S65.** Interaction detail and interaction residue of **3f** against *Acanthamoeba castellanii* CYP51

**Interaction detail and interaction residue of tested compounds against *Acanthamoeba castellanii* profilin IA**

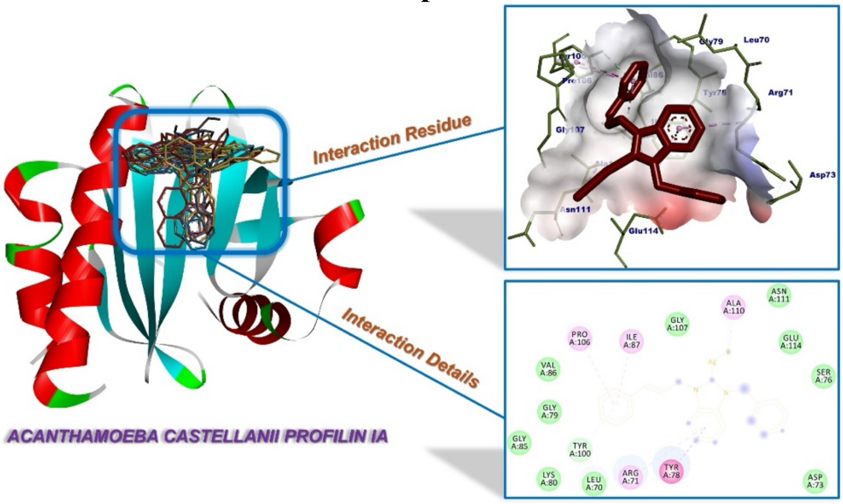


**Figure S66.** Interaction detail and interaction residue of **1a** against *Acanthamoeba castellanii* profilin IA

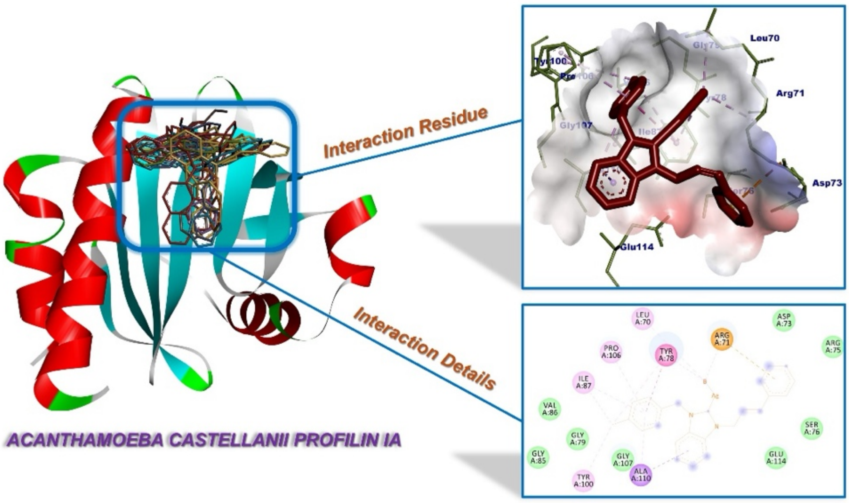


**Figure S67.** Interaction detail and interaction residue of **1b** against *Acanthamoeba castellanii* profilin IA

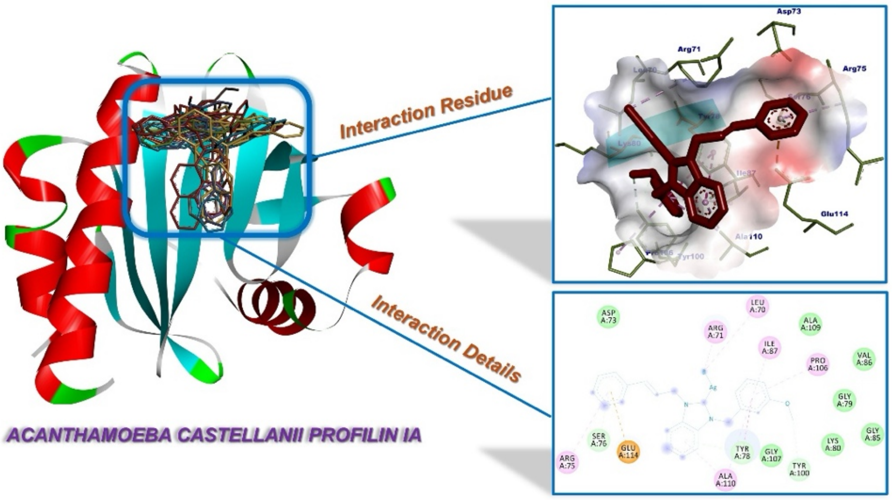


**Figure S68.** Interaction detail and interaction residue of **1c** against *Acanthamoeba castellanii* profilin IA

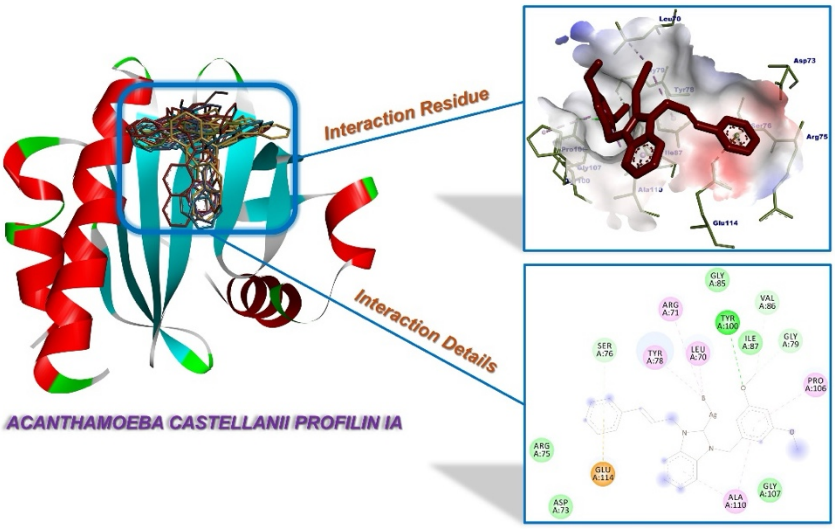


**Figure S69.** Interaction detail and interaction residue of **1d** against *Acanthamoeba castellanii* profilin IA

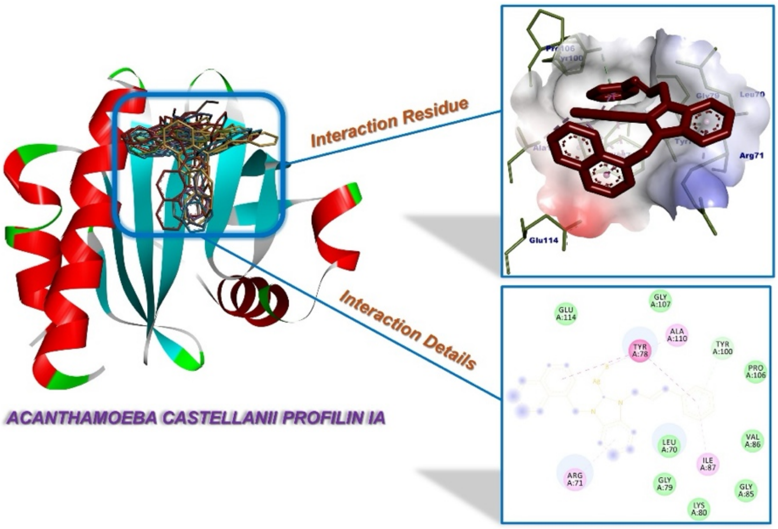


**Figure S70.** Interaction detail and interaction residue of **1e** against *Acanthamoeba castellanii* profilin IA

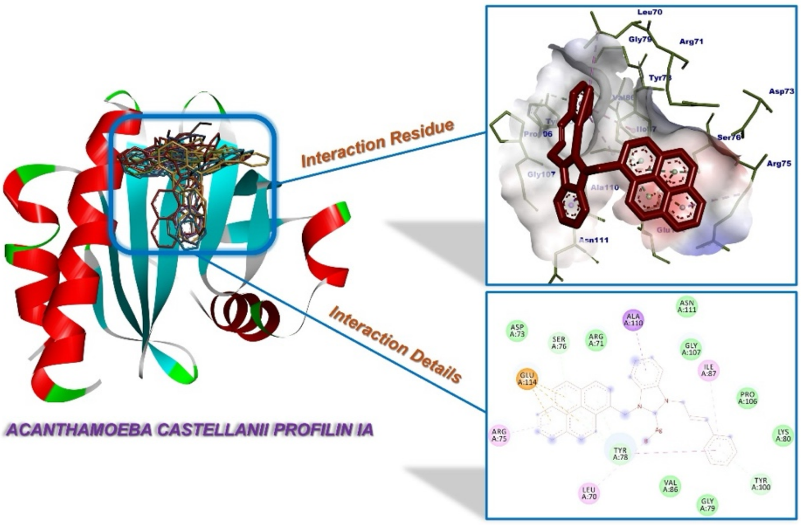


**Figure S71.** Interaction detail and interaction residue of **1f** against *Acanthamoeba castellanii* profilin IA

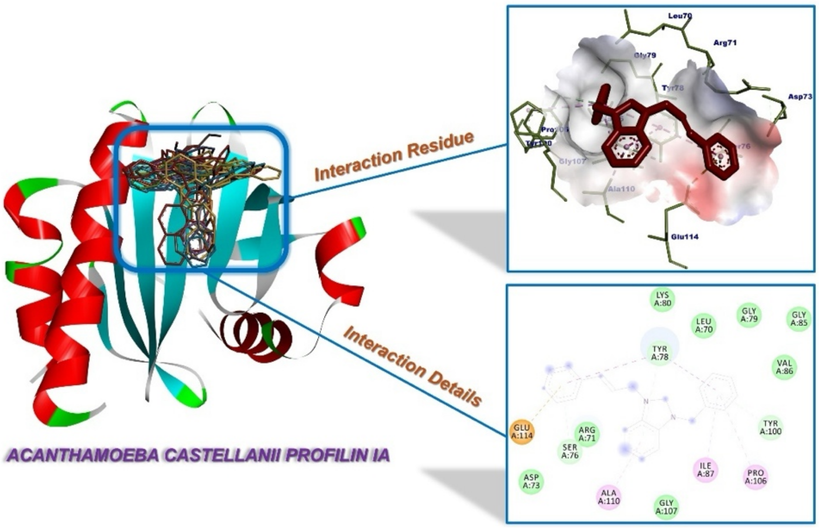


**Figure S72.** Interaction detail and interaction residue of **3a** against *Acanthamoeba castellanii* profilin IA

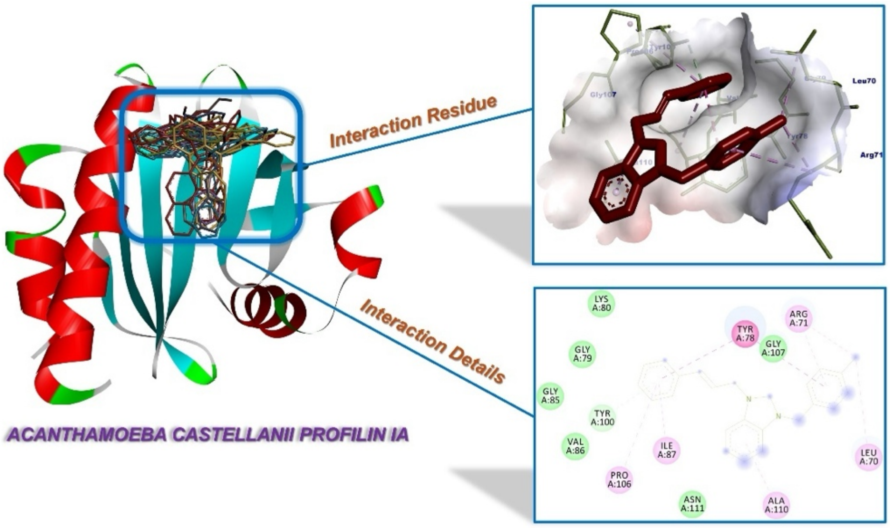


**Figure S73.** Interaction detail and interaction residue of **3b** against *Acanthamoeba castellanii* profilin IA

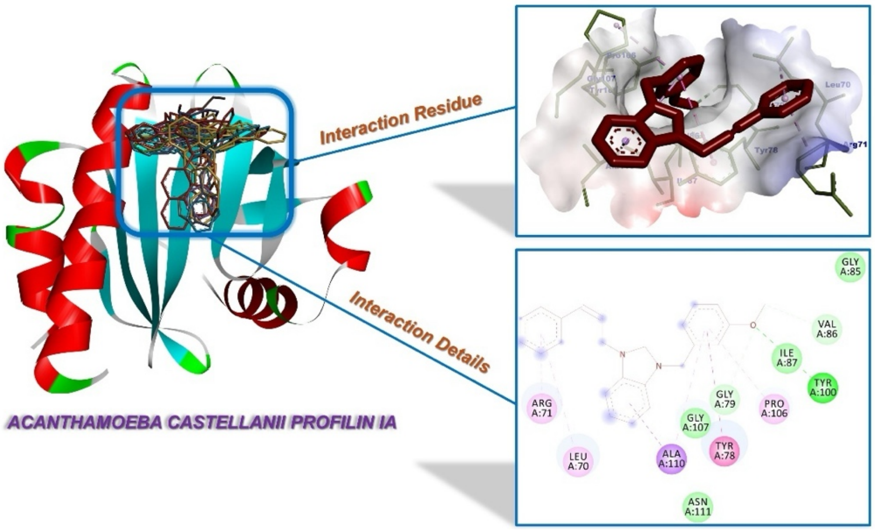


**Figure S74.** Interaction detail and interaction residue of **3c** against *Acanthamoeba castellanii* profilin IA

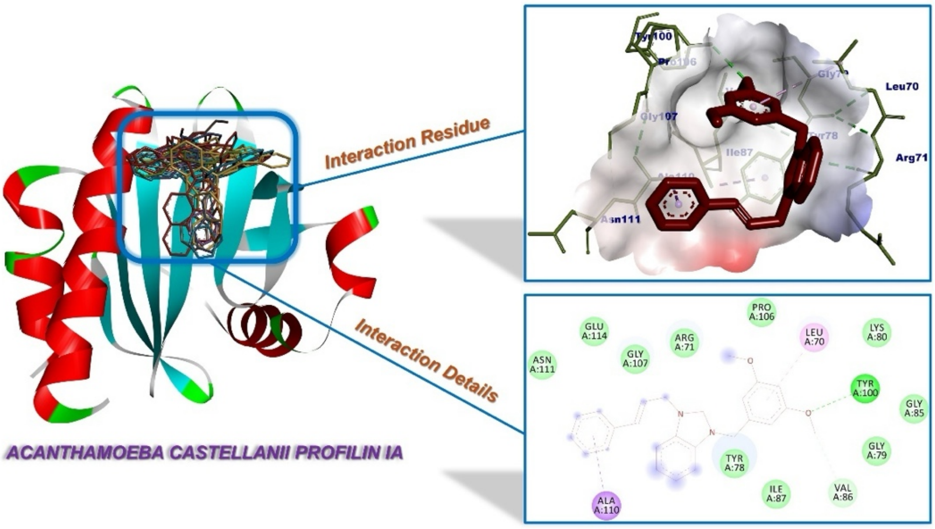


**Figure S75.** Interaction detail and interaction residue of **3d** against *Acanthamoeba castellanii* profilin IA

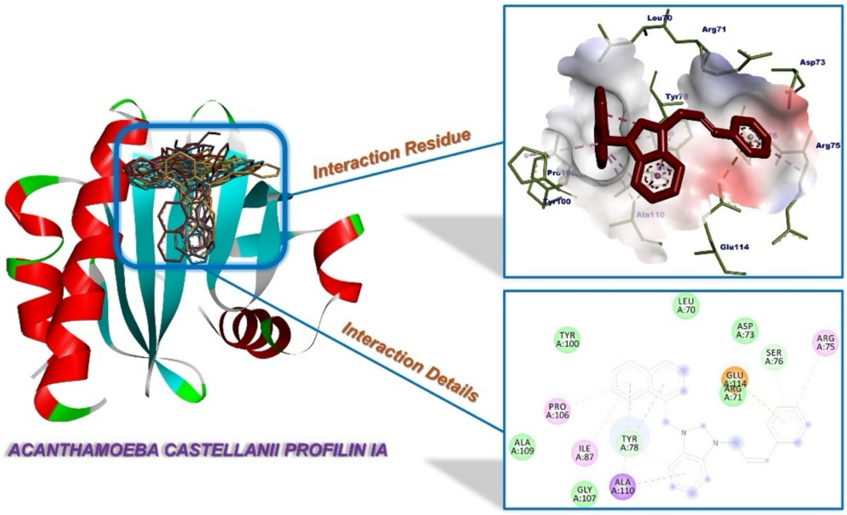


**Figure S76.** Interaction detail and interaction residue of **3e** against *Acanthamoeba castellanii* profilin IA

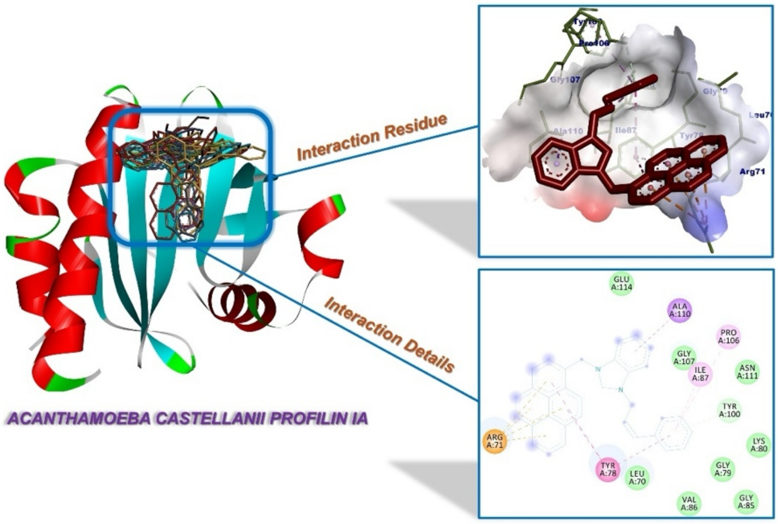


**Figure S77.** Interaction detail and interaction residue of **3f** against *Acanthamoeba castellanii* profilin IA

**Interaction detail and interaction residue of tested compounds against *Acanthamoeba castellanii* profilin IB**

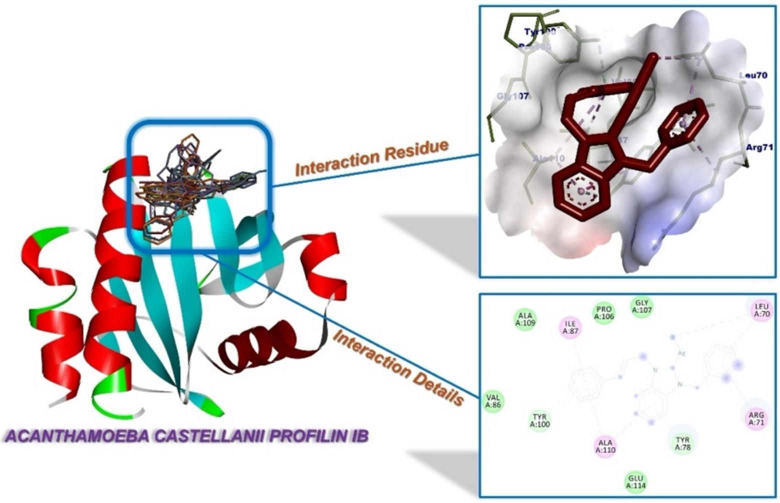


**Figure S78.** Interaction detail and interaction residue of **1a** against *Acanthamoeba castellanii* profilin IB

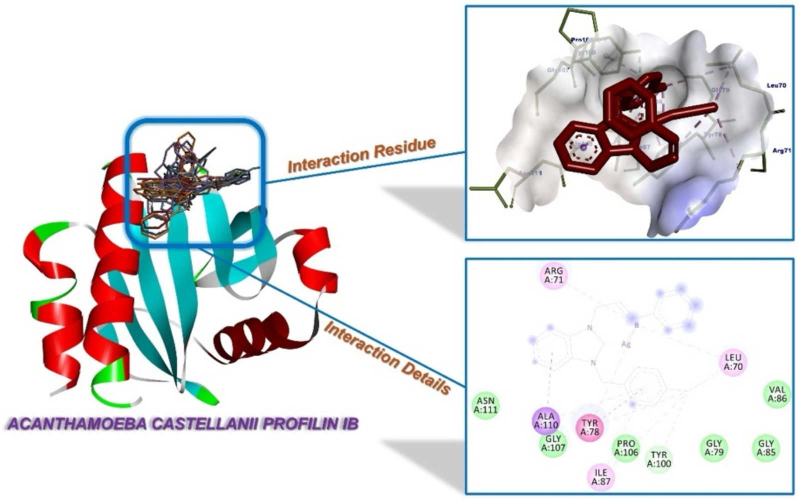


**Figure S79.** Interaction detail and interaction residue of **1b** against *Acanthamoeba castellanii* profilin IB

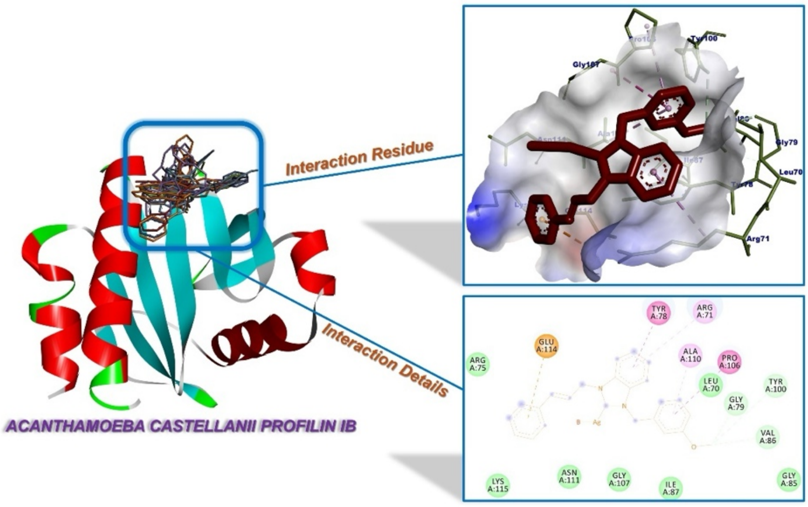


**Figure S80.** Interaction detail and interaction residue of **1c** against *Acanthamoeba castellanii* profilin IB

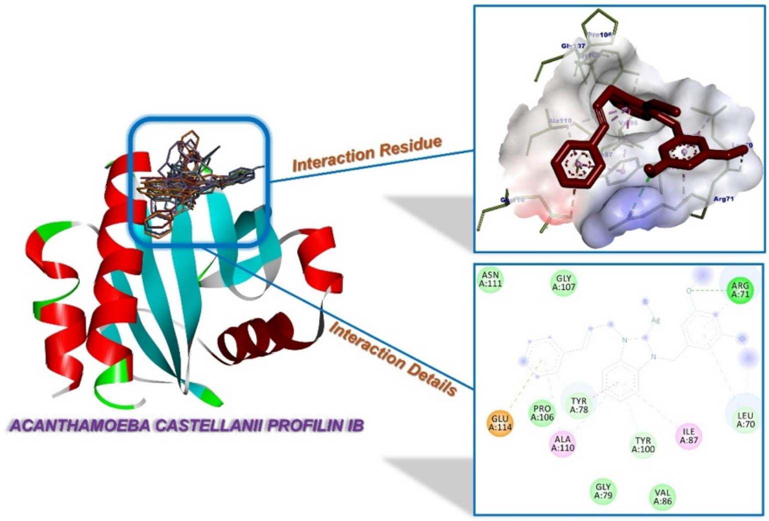


**Figure S81.** Interaction detail and interaction residue of **1d** against *Acanthamoeba castellanii* profilin IB

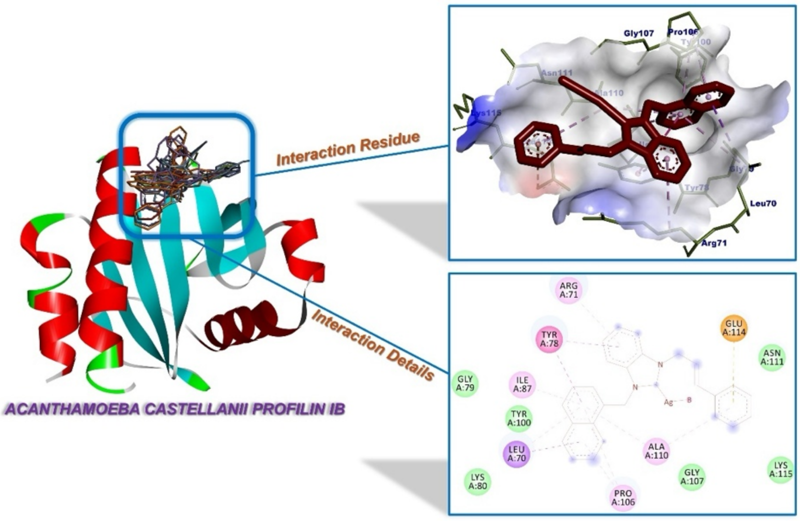


**Figure S82.** Interaction detail and interaction residue of **1e** against *Acanthamoeba castellanii* profilin IB

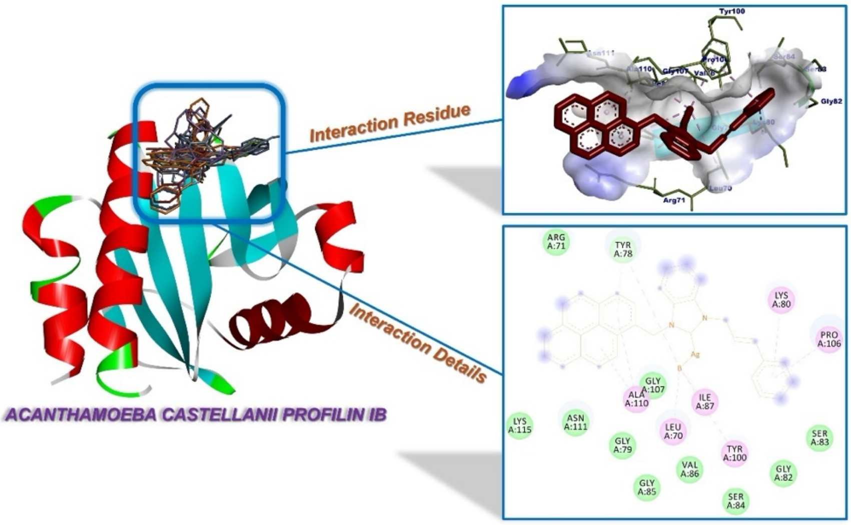


**Figure S83.** Interaction detail and interaction residue of **1f** against *Acanthamoeba castellanii* profilin IB

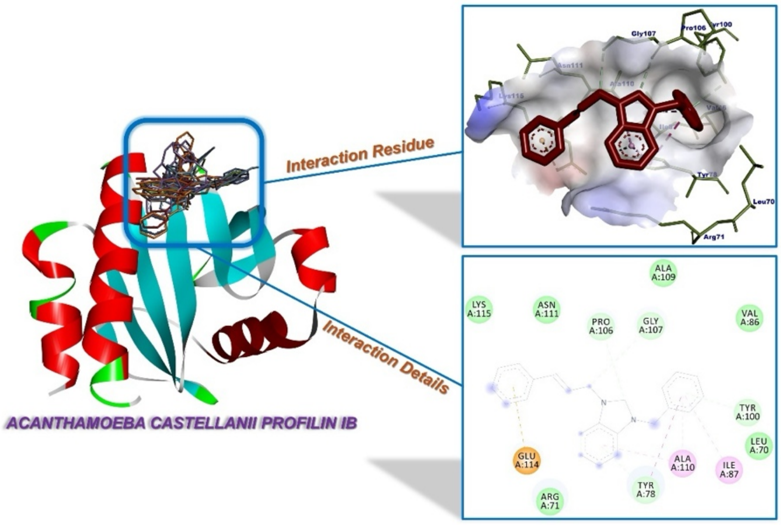


**Figure S84.** Interaction detail and interaction residue of **3a** against *Acanthamoeba castellanii* profilin IB

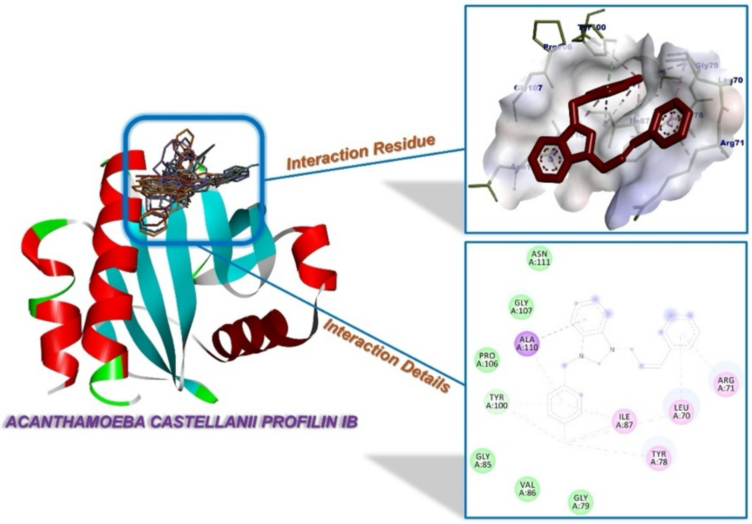


**Figure S85.** Interaction detail and interaction residue of **3b** against *Acanthamoeba castellanii* profilin IB

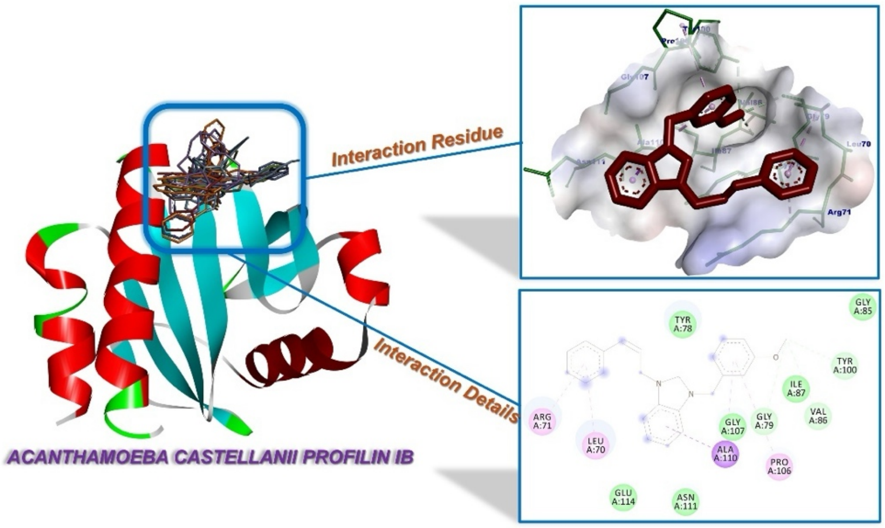


**Figure S86.** Interaction detail and interaction residue of **3c** against *Acanthamoeba castellanii* profilin IB

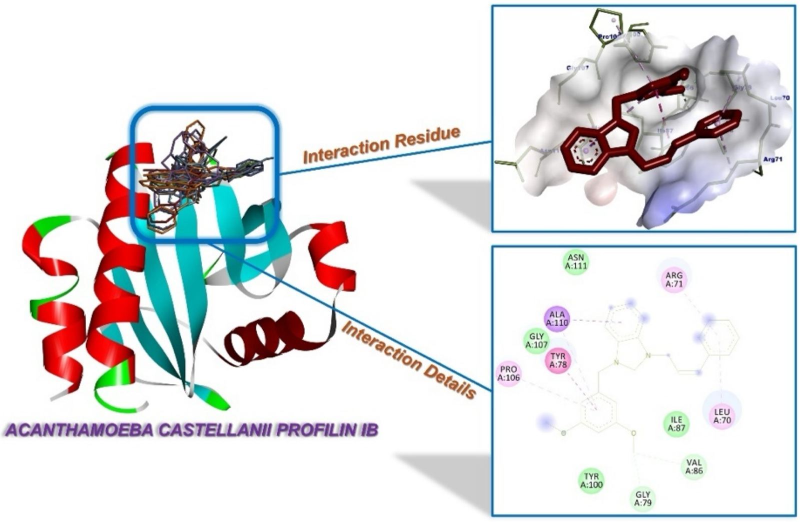


**Figure S87.** Interaction detail and interaction residue of **3d** against *Acanthamoeba castellanii* profilin IB

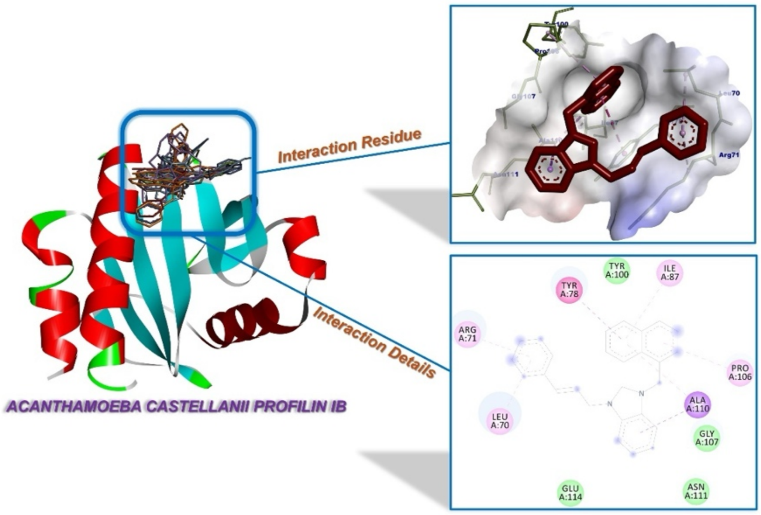


**Figure S88.** Interaction detail and interaction residue of **3e** against *Acanthamoeba castellanii* profilin IB

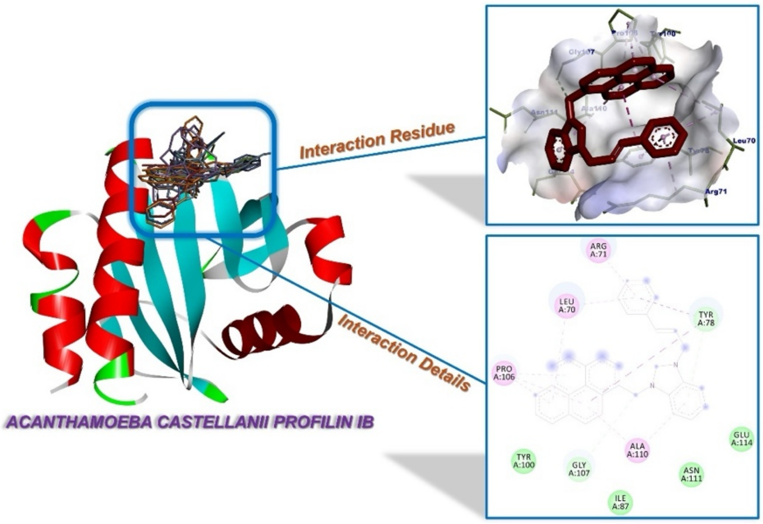


**Figure S89.** Interaction detail and interaction residue of **3f** against *Acanthamoeba castellanii* profilin IB

**Interaction detail and interaction residue of tested compounds against *Acanthamoeba castellanii* profilin II**

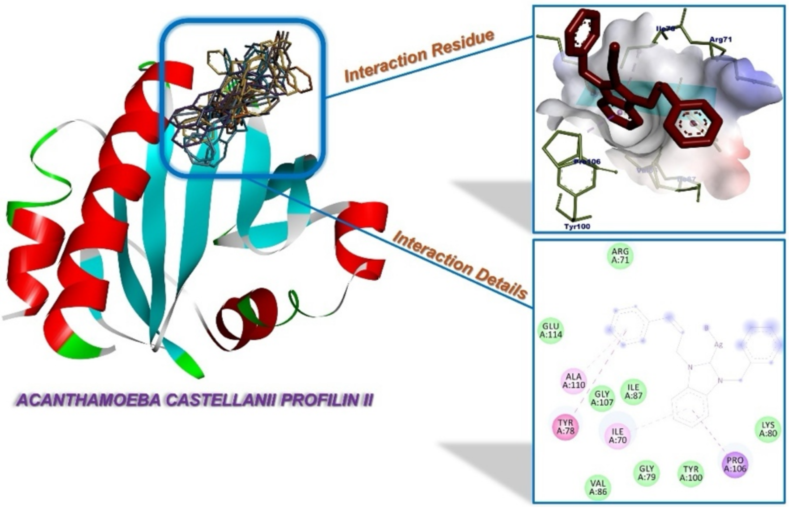


**Figure S90.** Interaction detail and interaction residue of **1a** against *Acanthamoeba castellanii* profilin II

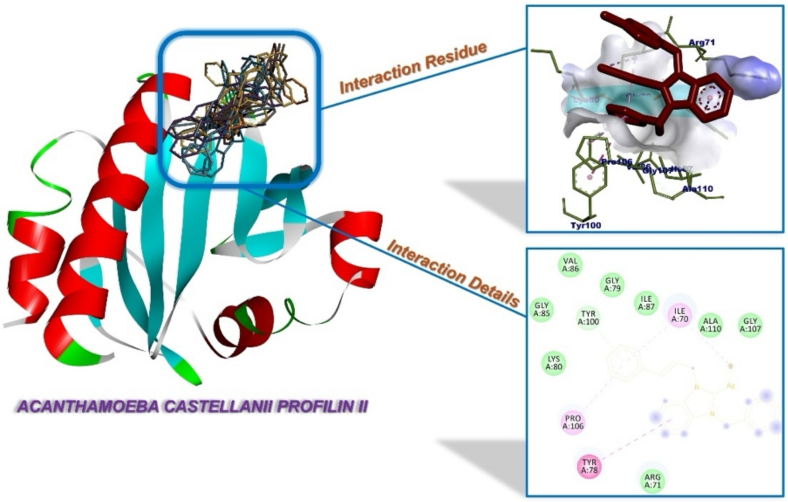


**Figure S91.** Interaction detail and interaction residue of **1b** against *Acanthamoeba castellanii* profilin II

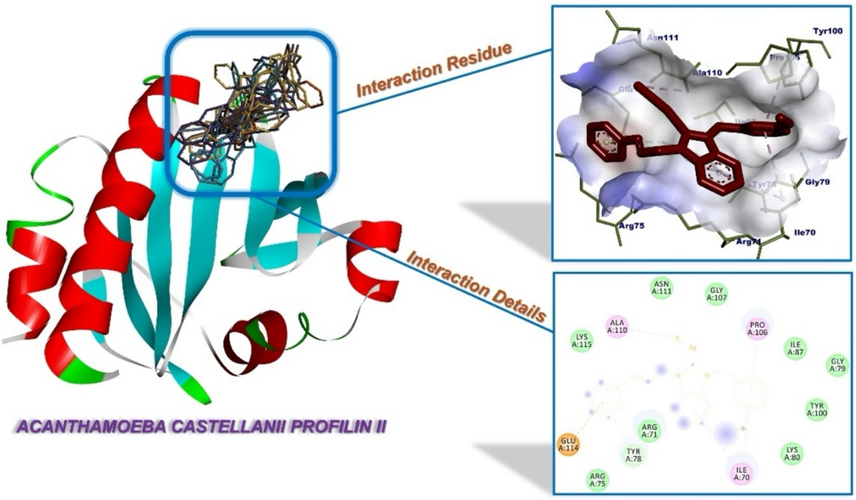


**Figure S92.** Interaction detail and interaction residue of **1c** against *Acanthamoeba castellanii* profilin II

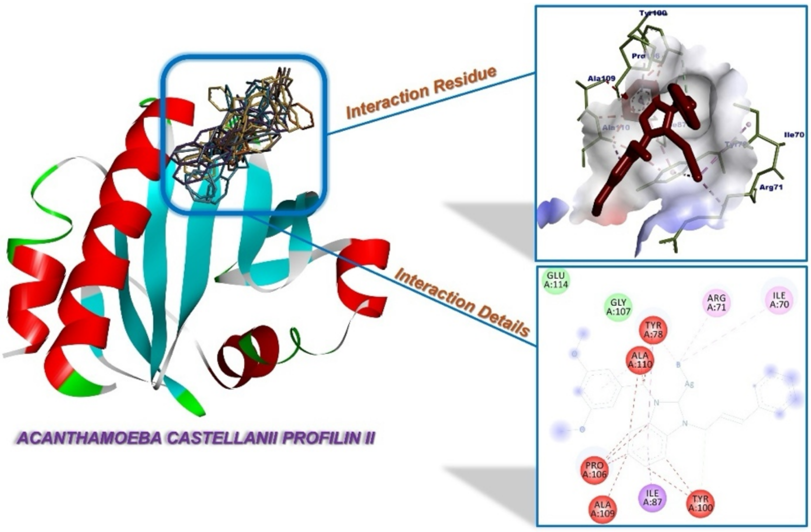


**Figure S93.** Interaction detail and interaction residue of **1d** against *Acanthamoeba castellanii* profilin II

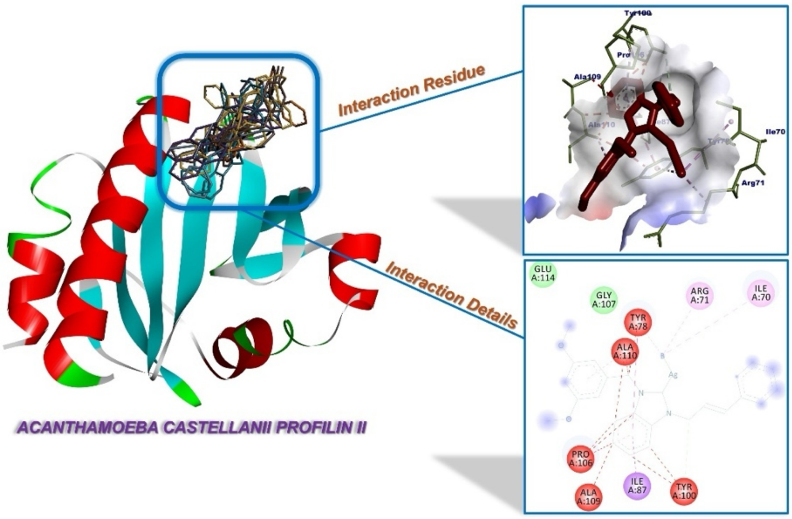


**Figure S94.** Interaction detail and interaction residue of **1e** against *Acanthamoeba castellanii* profilin II

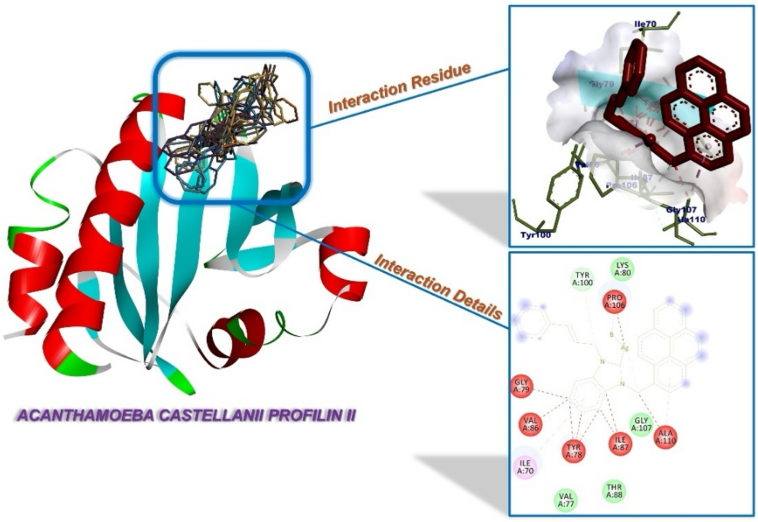


**Figure S95.** Interaction detail and interaction residue of **1f** against *Acanthamoeba castellanii* profilin II

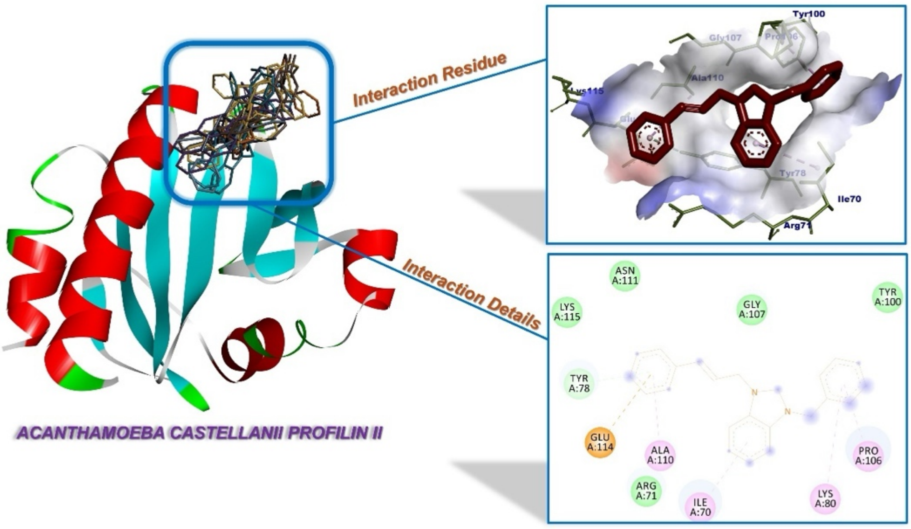


**Figure S96.** Interaction detail and interaction residue of **3a** against *Acanthamoeba castellanii* profilin II

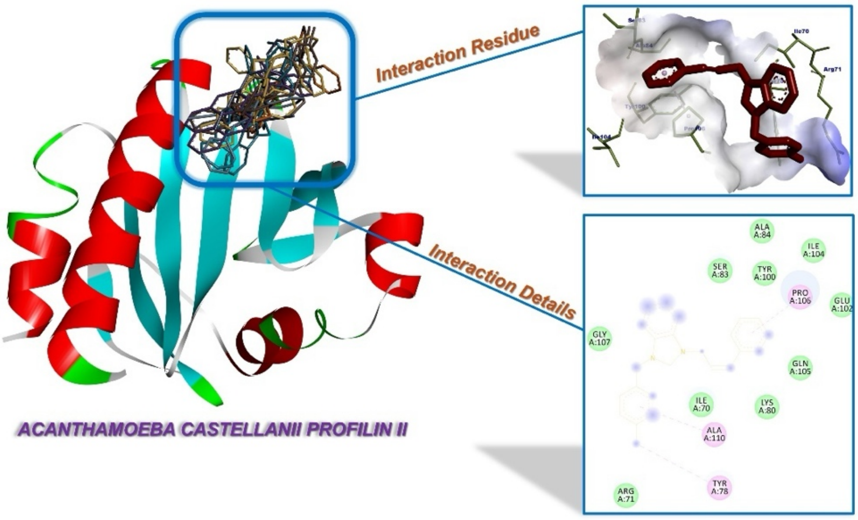


**Figure S97.** Interaction detail and interaction residue of **3b** against *Acanthamoeba castellanii* profilin II

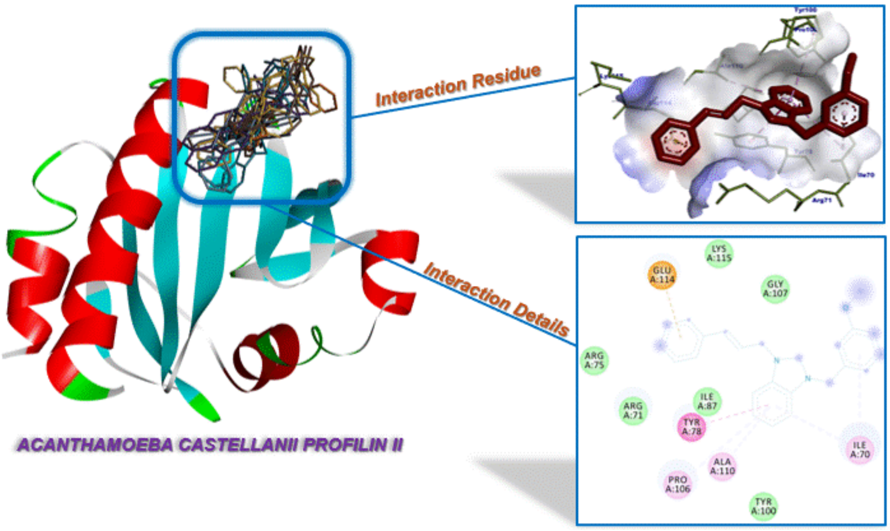


**Figure S98.** Interaction detail and interaction residue of **3c** against *Acanthamoeba castellanii* profilin II

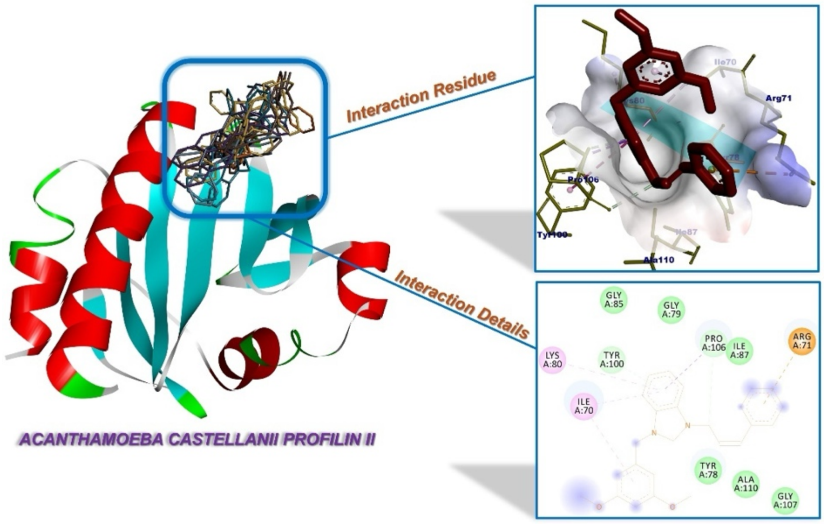


**Figure S99.** Interaction Detail and Interaction Residue of **3d** against *Acanthamoeba castellanii* Profilin II

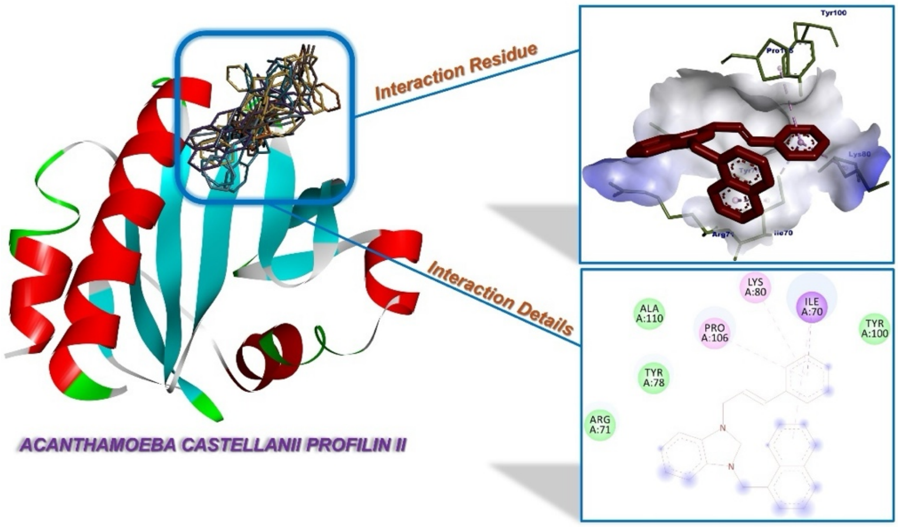


**Figure S100.** Interaction Detail and Interaction Residue of **3e** against *Acanthamoeba castellanii* Profilin II

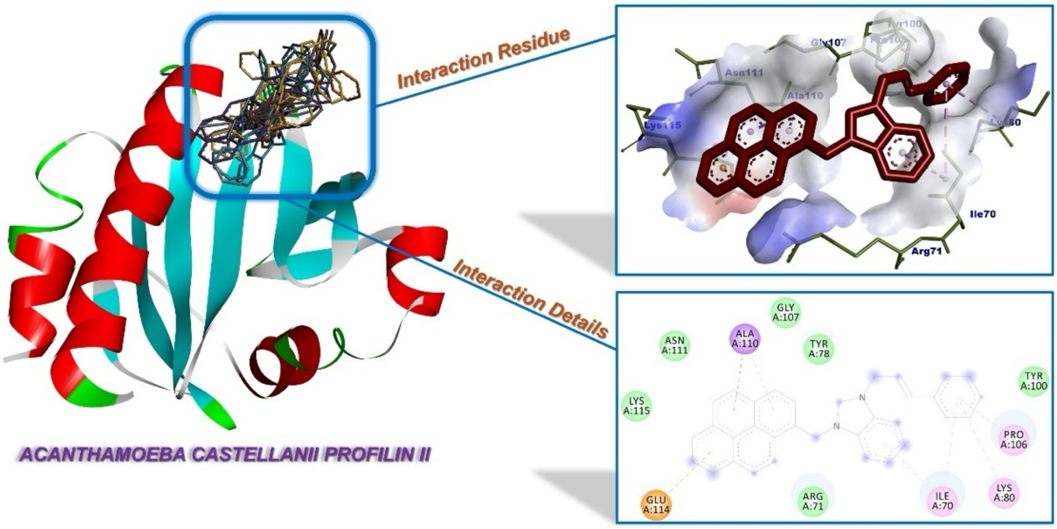


**Figure S101.** Interaction Detail and Interaction Residue of **3f** against *Acanthamoeba castellanii* Profilin II
